# New Material of *Beelzebufo*, a Hyperossified Frog (Amphibia: Anura) from the Late Cretaceous of Madagascar

**DOI:** 10.1371/journal.pone.0087236

**Published:** 2014-01-28

**Authors:** Susan E. Evans, Joseph R. Groenke, Marc E. H. Jones, Alan H. Turner, David W. Krause

**Affiliations:** 1 Department of Cell and Developmental Biology, University College London, London, United Kingdom; 2 Department of Anatomical Sciences, Stony Brook University, Stony Brook, New York, United States of America; 3 School of Earth and Environmental Sciences, The University of Adelaide, Adelaide, South Australia, Australia; College of the Holy Cross, United States of America

## Abstract

The extant anuran fauna of Madagascar is exceptionally rich and almost completely endemic. In recent years, many new species have been described and understanding of the history and relationships of this fauna has been greatly advanced by molecular studies, but very little is known of the fossil history of frogs on the island. *Beelzebufo ampinga*, the first named pre-Holocene frog from Madagascar, was described in 2008 on the basis of numerous disarticulated cranial and postcranial elements from the Upper Cretaceous (Maastrichtian) Maevarano Formation of Madagascar. These specimens documented the presence of a hyperossified taxon that differed strikingly from extant Malagasy frogs in its large size and heavy coarse cranial exostosis. Here we describe and analyse new, articulated, and more complete material of the skull, vertebral column, and hind limb, as well as additional isolated elements discovered since 2008. μCT scans allow a detailed understanding of both internal and external morphology and permit a more accurate reconstruction. The new material shows *Beelzebufo* to have been even more bizarre than originally interpreted, with large posterolateral skull flanges and sculptured vertebral spine tables. The apparent absence of a tympanic membrane, the strong cranial exostosis, and vertebral morphology suggest it may have burrowed during seasonally arid conditions, which have been interpreted for the Maevarano Formation from independent sedimentological and taphonomic evidence. New phylogenetic analyses, incorporating both morphological and molecular data, continue to place *Beelzebufo* with hyloid rather than ranoid frogs. Within Hyloidea, *Beelzebufo* still groups with the South American Ceratophryidae thus continuing to pose difficulties with both biogeographic interpretations and prior molecular divergence dates.

## Introduction

Madagascar is a large island landmass separated from Africa by the wide and deep Mozambique Channel. It has a unique and diverse herpetofauna including around 250 species of anurans [1]–[6], with an estimated 200 or more remaining to be described [7]–[8]. Although a few taxa have close relatives in Africa (e.g., *Ptychadena*), more than 90% of Malagasy anuran genera are endemic (e.g., mantellids, sensu [9]; Malagasy microhylids). Until recently, much of the palaeobiogeographic discussion focused on hypotheses of vicariance in the context of Gondwanan fragmentation (e.g., [10]–[12]), but molecular phylogenetics has provided evidence of multiple dispersal events [5], [13]–[20], and there is a growing consensus that at least some of the extant anuran fauna of Madagascar arrived there after its isolation from the rest of Gondwana [3], [5], [13]–[18], [20]–[21].

Clearly, a good fossil record would contribute to increased understanding of the roles of extinction, vicariance, and dispersal in the history of the extant anuran assemblage of Madagascar. In addition to reports of specimens of microhylids and the introduced ranid *Hoplobatrachus* from the Holocene [22]–[23], the record was, until recently, limited to the Early Triassic stem-anuran *Triadobatrachus* [24] and a small sample of five isolated bones from the Upper Cretaceous Maevarano Formation [25]. Recovery of a much larger sample from the Maevarano Formation, including both cranial and postcranial elements, over the course of several subsequent expeditions permitted the description of a new genus and species, *Beelzebufo ampinga* [26], a large broad-headed, hyperossified, terrestrial anuran, unlike any that exists on Madagascar today. Phylogenetic analysis placed *Beelzebufo* with the specialized extant South American ‘horned frogs’, the Ceratophryidae (sensu [27]: *Ceratophrys*, *Lepidobatrachus*, *Chacophrys*) and the South American fossil taxa *Baurubatrachus* (Maastrichtian) and *Wawelia* (Miocene). This, in turn, was taken to indicate support for the hypothesis of a link between South America and Madagascar via Antarctica and the Kerguelen Plateau until the later stages of the Late Cretaceous [28]. However, both the phylogenetic and palaeogeographical hypotheses of Evans et al. [26] have subsequently been challenged. Ruane et al. [29] reran the phylogenetic analysis using both a morphological data set and a combined molecular + morphological data set. Although they obtained the same tree topology as Evans et al. [26], they did not accept *Beelzebufo* as a crown ceratophryid, based on the weak tree support and on molecular divergence estimates placing the origin and diversification of ceratophryids in the Neogene (see also [30]). Similarly, Ali and Aitchison [31]–[32] (see also [33]) rejected the palaeogeographical scenario of Hay et al. [28] on the basis of more recent geophysical and geological evidence demonstrating that connections between Antarctica and Indo-Madagascar were severed by the Middle Aptian (∼115–120 Ma), and that only a small fraction of the Kerguelen Plateau was emergent in the later stages of the Late Cretaceous.

**Figure 1:**
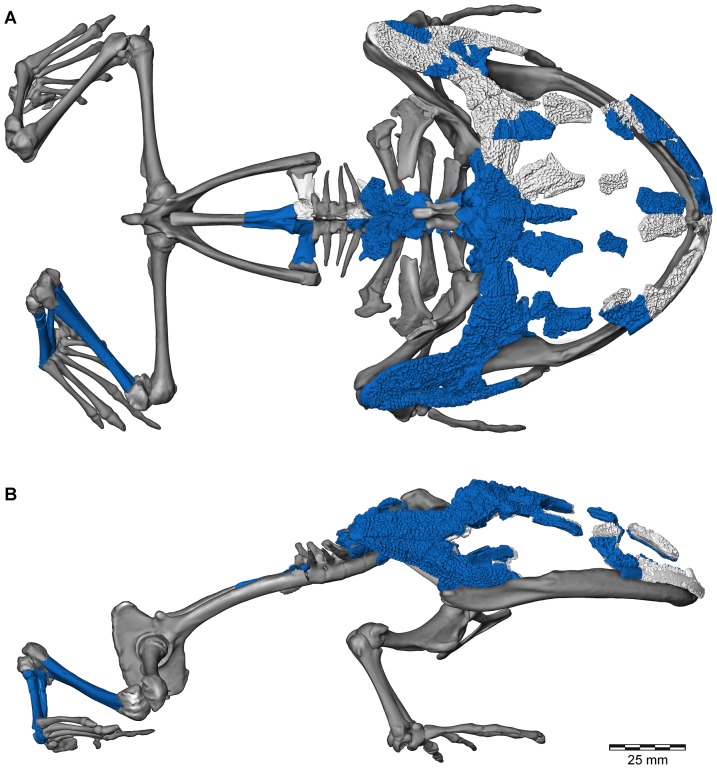
Three-dimensional digital reconstruction of skeleton of *Beelzebufo ampinga* highlighting sources of material for reconstruction. **A**, dorsal view; and **B**, right lateral view (with left limbs removed for visual clarity). *Beelzebufo* specimens used in model in dark blue. Light grey cranial and vertebral materials inferred from known morphology of *Beelzebufo* specimens, primarily through mirror-imaging. Dark grey jaws and postcranial elements modelled on large female specimen of *Ceratophrys aurita* (LACM 163430). See Supporting Information S1 for detailed description of model.

**Figure 2:**
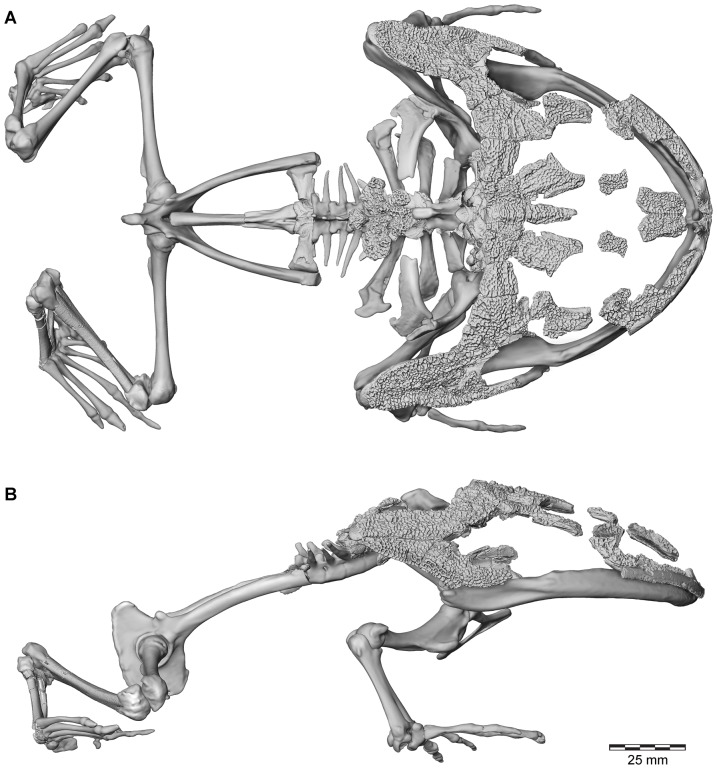
Three-dimensional digital reconstruction of skeleton of *Beelzebufo ampinga*. **A**, dorsal view; and **B**, right lateral view (with left limbs removed for visual clarity). See Figure 1 for sources of material for reconstruction, and Supporting Information S1 for detailed description of model.

**Figure 3:**
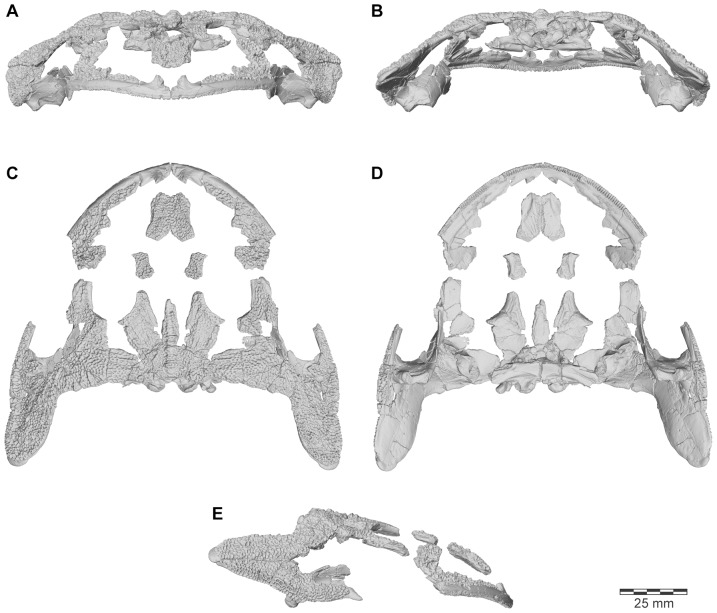
Three-dimensional digital reconstruction of skull of *Beelzebufo ampinga*. **A**, anterior; **B**, posterior; **C**, dorsal; **D**, ventral; and **E**, right lateral views. Parts of posterior region of skull lack complete symmetry because respective sides use different combinations of specimens. See Supporting Information S1 for detailed description of model.

**Figure 4:**
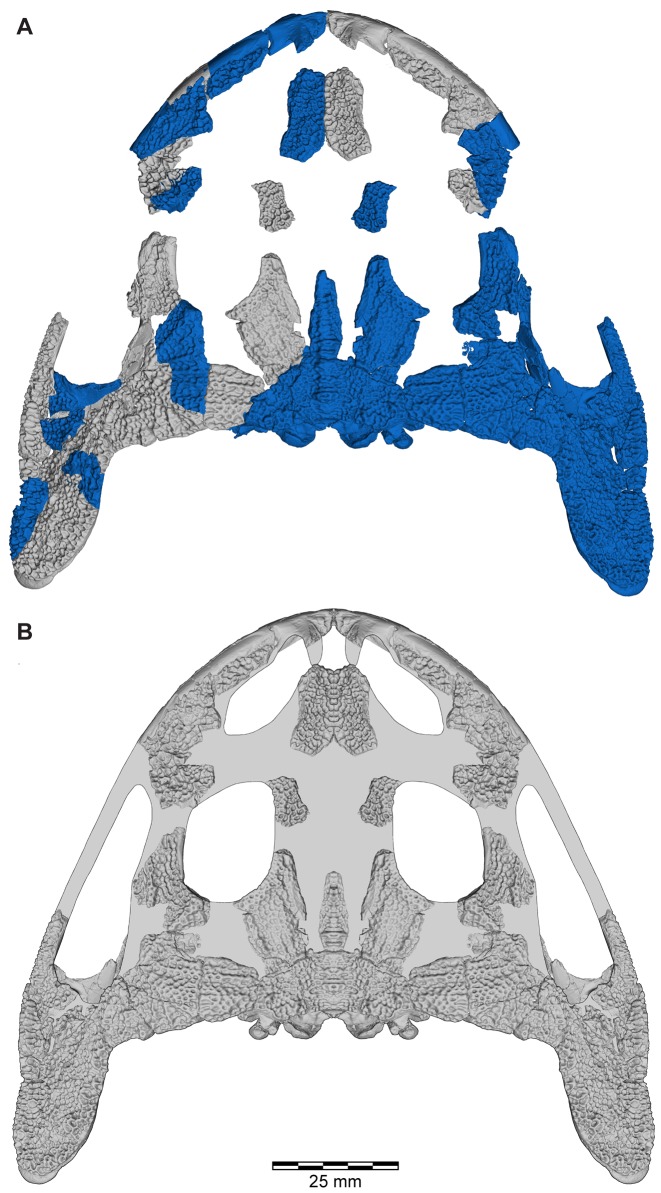
Skull reconstructions of *Beelzebufo ampinga*. **A**, dorsal view, as in Fig. 3C, with areas of digital model representing actual (non-mirrored) specimens in dark blue; **B**, dorsal view, illustrated reconstruction based on Fig. 3C, but with right side of 3C mirrored for symmetry and with missing regions silhouetted in grey. Shape of orbital, narial, and temporal fenestrae based on bone extrapolation from edges, facets, and other anatomical features. See Supporting Information S1 for detailed description of model.

**Figure 5:**
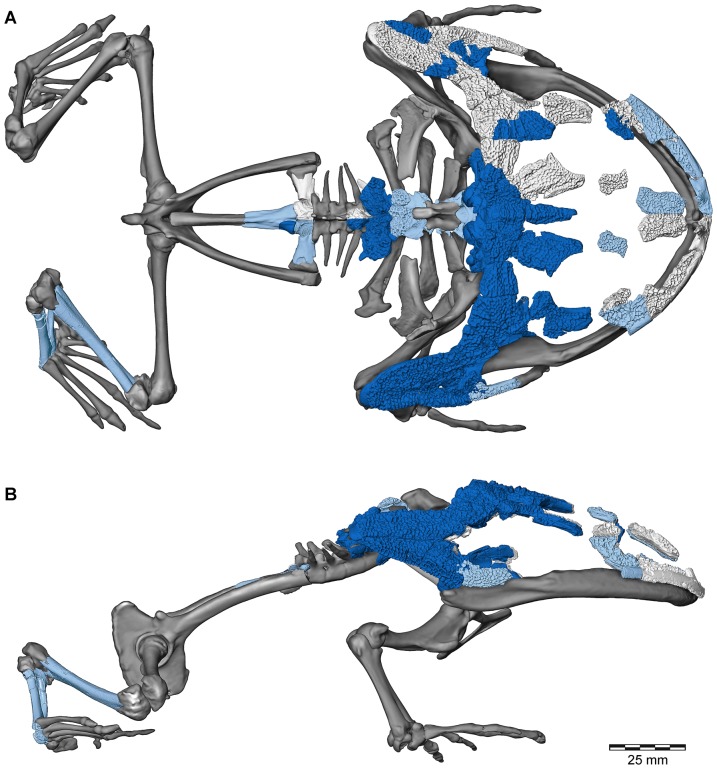
Three-dimensional digital reconstruction of skeleton of *Beelzebufo ampinga* highlighting specimen FMNH PR 2512. Elements of skeleton of FMNH PR 2512, the most complete specimen discovered to date, highlighted in dark blue. Other *Beelzebufo* specimens in light blue. Light grey cranial and vertebral materials inferred from known morphology of *Beelzebufo*, created primarily through mirror-imaging. Dark grey postcranial elements and jaws modelled on large female specimen of *Ceratophrys aurita* (LACM 163430). See Supporting Information S1 for detailed description of model.

Since the original description of *Beelzebufo ampinga* [26], numerous additional isolated cranial elements of this species have been discovered, as well as several presacral vertebrae and a tarsal bone. Of most significance, however, was the recovery, during the field season of 2010, of an articulated partial cranium of *B. ampinga*, in association with several presacral vertebrae (FMNH PR 2512). This new material, particularly the cranium, confirms some aspects of the original interpretation but necessitates a reconsideration of others (Table S1 in File S1). It also adds important new data that permits a reconstruction of the skull and skeleton (Figs 1–5: see Supporting Information for 3-D animations, Videos S1, S2 and S3), showing *Beelzebufo* to have been even more bizarre and heavily armoured than earlier reconstructions depicted ([26]:fig. 2), and forms the basis of new phylogenetic analyses, using both morphological and combined datasets.

### Geological context and fossil materials

**Figure 6:**
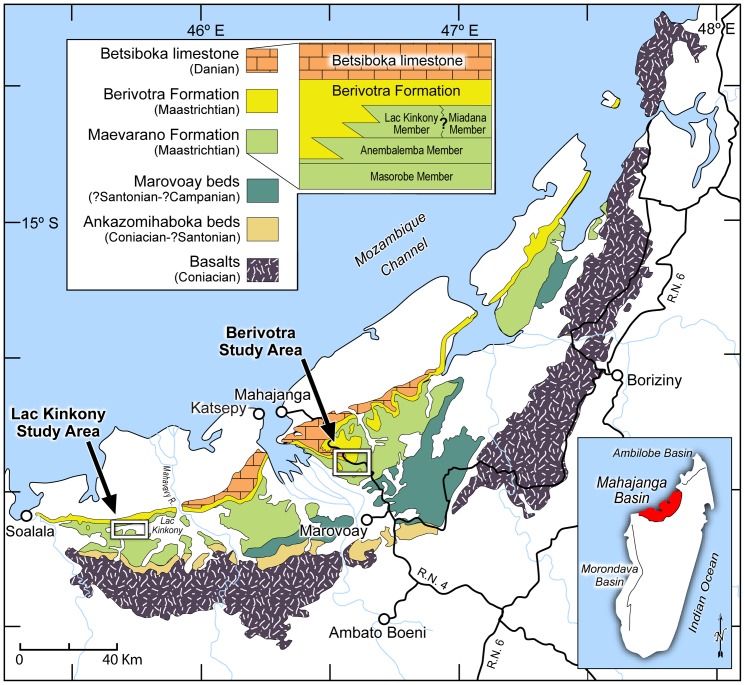
Map of Mahajanga Basin study areas and stratigraphy (modified from Rogers et al., 2013: fig. 1). The majority of specimens of *Beelzebufo* have been discovered in the Anembalemba Member of the Maevarano Formation in the Berivotra Study Area, but the taxon has also been recovered from the Masorobe Member in the Berivotra Study Area and the Lac Kinkony Member in the Lac Kinkony Study Area.

**Figure 7:**
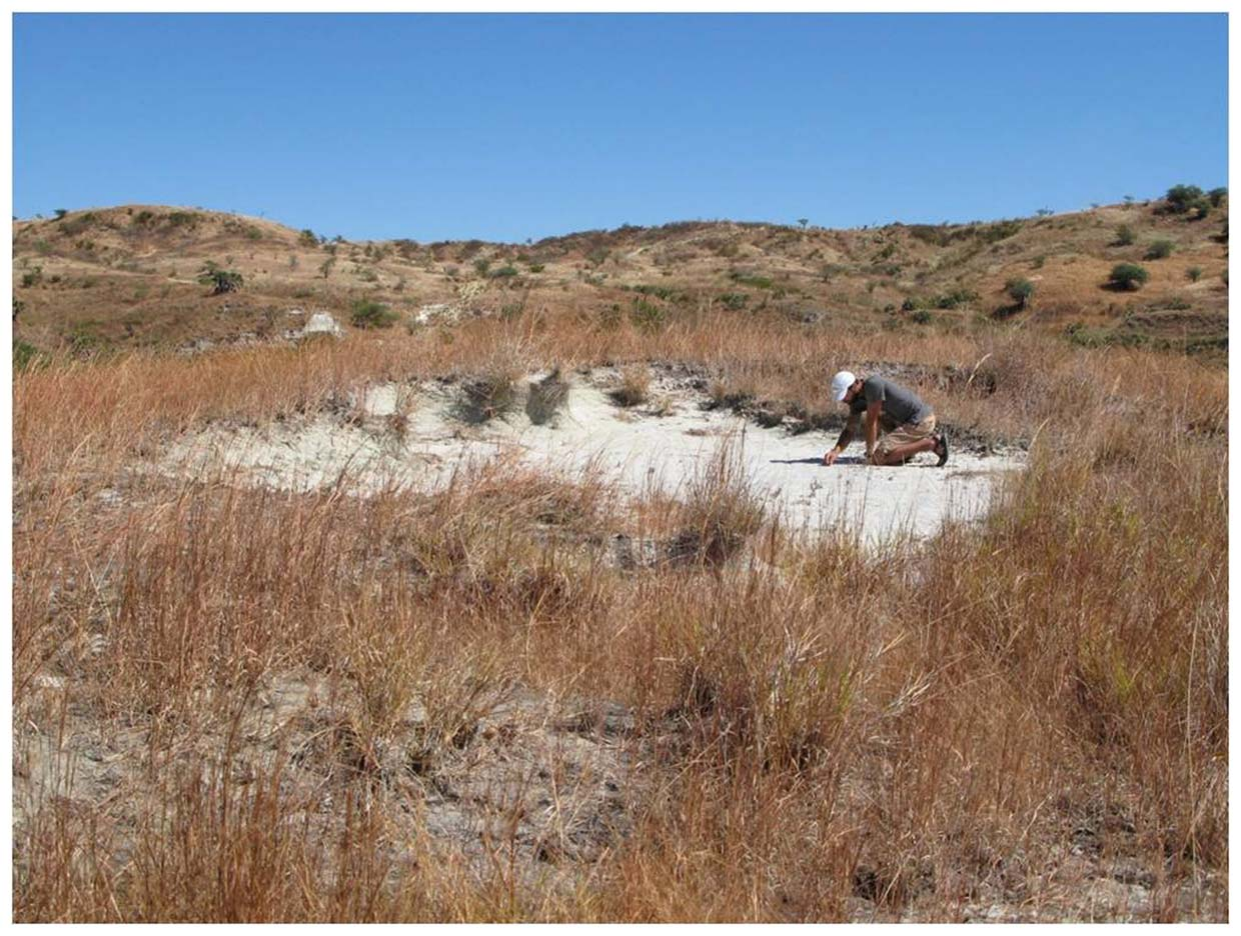
Mahajanga Basin Project locality MAD98-25. The image, taken in July 2007, shows a member of the field crew surface collecting at the locality from which the most complete specimen of *Beelzebufo ampinga*, FMNH PR 2512, was recovered during 11 expeditions between 1998 and 2011.

*Beelzebufo* is now represented by 64 specimens (mostly partial skull elements) from 27 localities within the non-marine Maevarano Formation in the Berivotra Study Area of the Mahajanga Basin, northwestern Madagascar (Figs 6–7). Most of the specimens described herein were collected from the richly fossiliferous Anembalemba Member, but a few are from the underlying Masorobe Member and a small subset was recovered from the overlying Lac Kinkony Member in the Lac Kinkony Study Area [34]. The latter is situated in the same basin as the Berivotra Study Area, but lies west, not east, of the Betsiboka River. The Anembalemba and Masorobe members crop out in both the Berivotra and Lac Kinkony study areas, whereas the Lac Kinkony Member is only known from the latter.

The Mahajanga Basin Project, conducted jointly by Stony Brook University and the University of Antananarivo, was initiated in 1993; the anuran specimens described herein were collected during the course of 11 expeditions between 1993 and 2011. Though the Maevarano Formation was previously ascribed ages ranging from Turonian to Campanian (e.g., [35]–[40]), there is no litho-, bio-, or magnetostratigraphic evidence to indicate it is anything other than Maastrichtian in age [34], [41].

In the Berivotra Study Area, the Anembalemba Member consists of approximately 10–15 metres of sandstone-dominated lithologies that overlie the much thicker (>80 m) Masorobe Member. The latter is dominated by well-developed palaeosols and reveals multiple features consistent with the inference that it was deposited under semi-arid conditions on a well-drained floodplain spanning the crystalline highlands to the east and the Mozambique Channel to the west [41], [42]. Vertebrate fossils in the Masorobe Member are much less common and less well preserved than in the overlying Anembalemba Member, which contains two discrete sandstone facies, designated Facies 1 and Facies 2 [42]. Facies 1 is comprised of light-coloured (light grey to white), moderately sorted (fine- to medium-grained) sandstones with prevalent tabular and cross-stratification representing normal streamflow. Facies 2 lithologies, by contrast, are darker (light olive green), more clay-rich, more poorly sorted (fine- to coarse-grained), and massive in structure. Rogers [43] interpreted Facies 2 as representing massive debris flows that presumably occurred during exceptional deluges in the rainy season and resulted in intense erosion and flooding. Most of the well-preserved vertebrate material, including that of the anuran, was found weathering out of Facies 2 sandstones. Isolated elements of *Beelzebufo* have more recently also been recovered from the Lac Kinkony Member, which overlies the Anembalemba Member and is capped by marine claystones and marlstones of the Berivotra Formation [34]. The Lac Kinkony Member, <20 m thick, consists of lithologies (siltstones, sandstones with dolomitic mud matrix, dolostones) interpreted to represent a previously unsampled nearshore, peritidal environment that was dissected by tidally influenced rivers. It is the only member of the Maevarano Formation that exhibits a strong marine influence.

**Figure 8:**
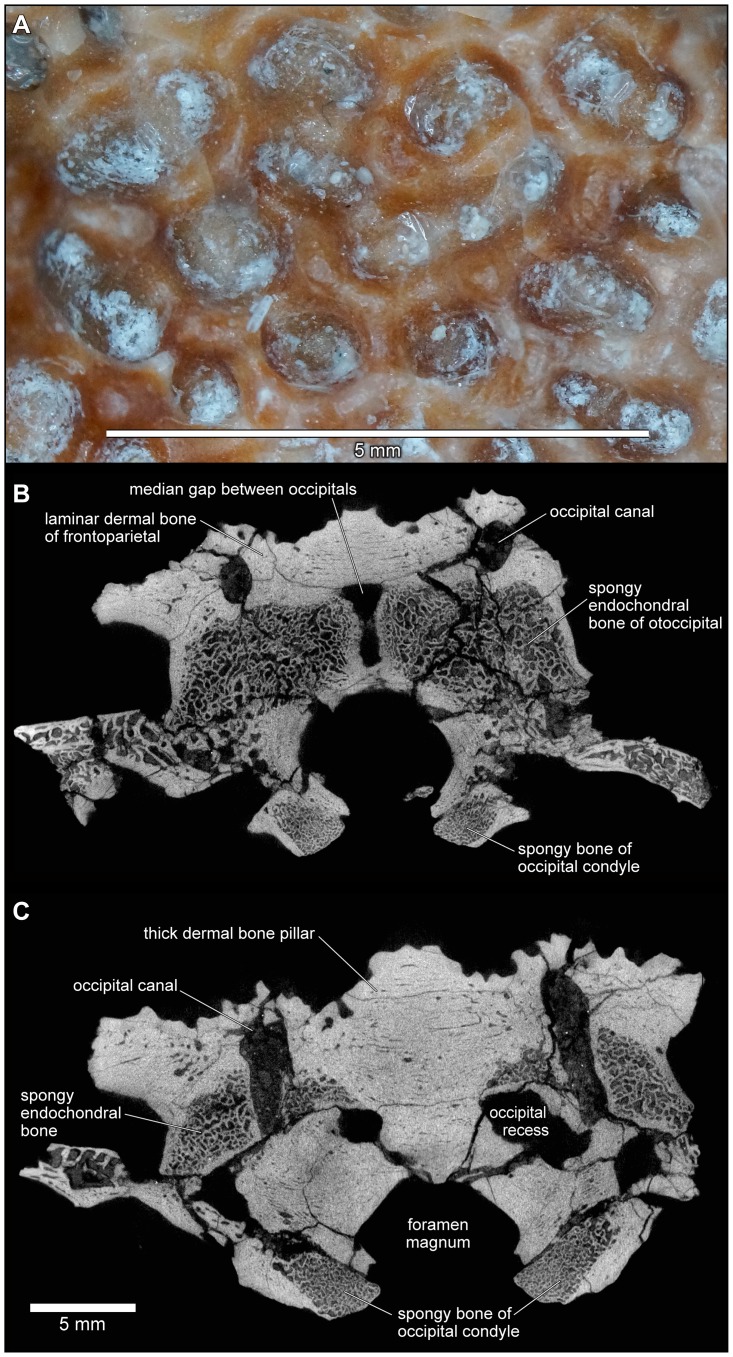
FMNH PR 2512 cranial bone morphology. **A**, detail of external exostosis on right squamosal; **B**, slices through posterior part of frontoparietal-braincase region showing details of thick laminar dermal bone overlying spongy endochondral bone; **C**, as B, but through occipital pillar.

**Figure 9:**
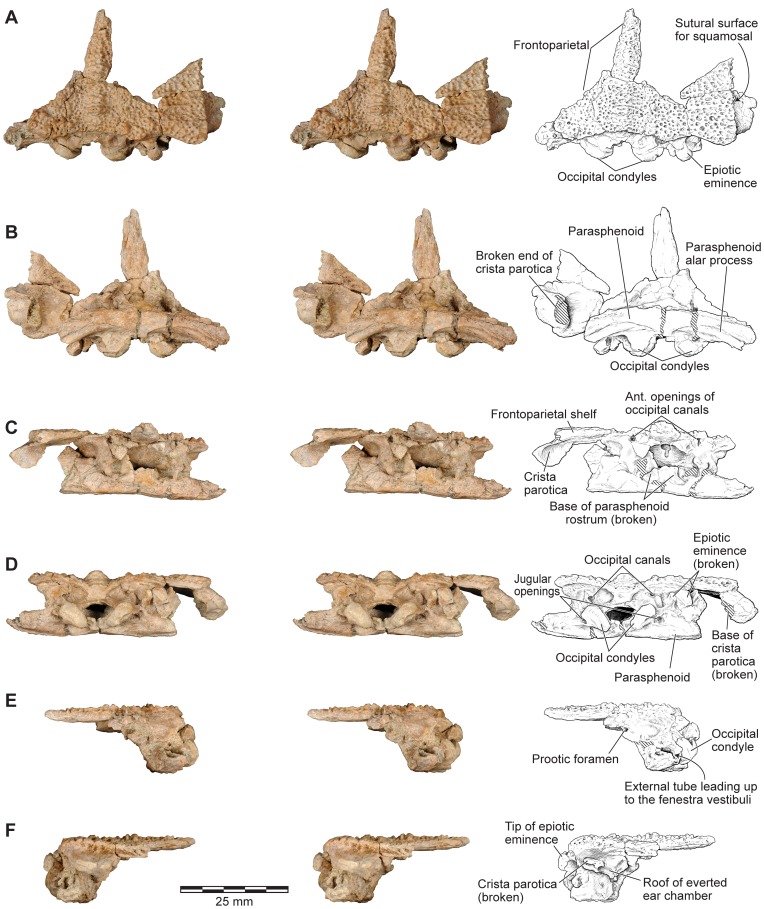
Stereophotographs of braincase and frontoparietal region of FMNH PR 2512 with labeled line drawings. **A**, dorsal; **B**, ventral; **C**, anterior; **D**, posterior; **E**, left lateral; and **F**, right lateral views.

**Figure 10:**
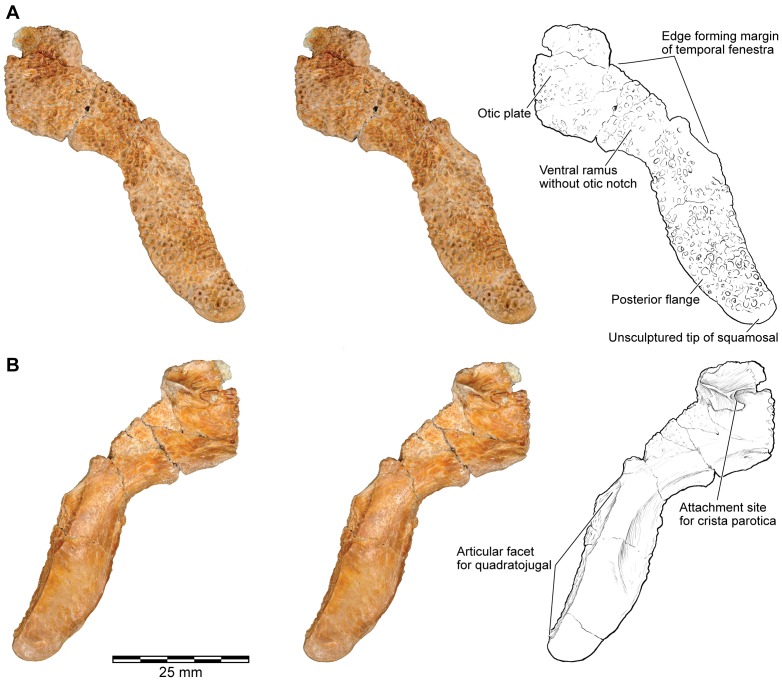
Stereophotographs of right squamosal of FMNH PR 2512 with labeled line drawings. **A**, dorsolateral; and **B**, ventromedial views.

**Figure 11:**
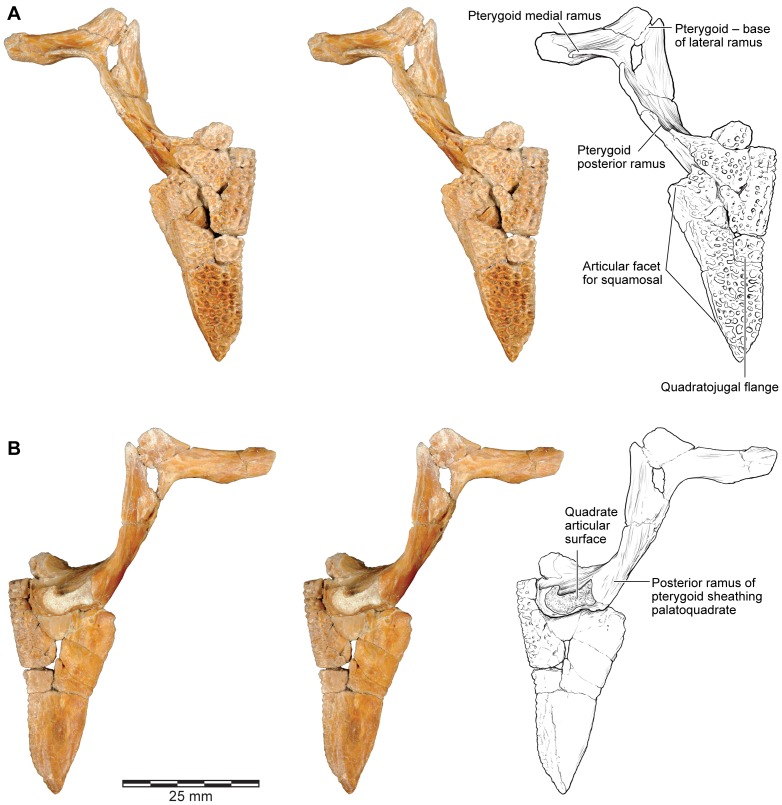
Stereophotographs of right quadratojugal-quadrate and pterygoid of FMNH PR 2512 with labeled line drawings. **A**, dorsolateral; and **B**, ventromedial views.

Until 2010, the material of *Beelzebufo* consisted entirely of disarticulated skull and postcranial elements, and fragments thereof, obtained primarily by surface collection but also by both dry and wet screening [25]–[26]; none were discovered during quarrying operations in the Berivotra Study Area that have yielded a plethora of partial and nearly complete skulls and/or skeletons of turtles [44], a lizard [45], snakes [46], crocodyliforms [47]–[50], avian and non-avian dinosaurs [51]–[56], and mammals [57]–[58]. The fragmentary skull elements of *Beelzebufo* were associated on the basis of their robusticity and the distinctive pattern of dermal sculpture (Fig. 8A) as well as consistent morphology and large size. Many of these identifications have been confirmed by the discovery in 2010 of a partial skull in association with several vertebrae, and with additional fragments of the same individual (FMNH PR 2512) recovered in 2011 (Figs 9–11). Isolated postcranial remains are attributed on the basis of large size, strong ossification, and, in the case of vertebrae, overlap with FMNH PR 2512. They are comparatively rare and comprise a tibiofibula, a tibiale-fibulare (astragalocalcaneum), and vertebrae including an atlas (fused with the second presacral), several presacrals, a partial sacral, and two partial urostyles. Although there are some additional small anuran remains (which will be described separately when more diagnostic material is recovered), there is no evidence that more than one taxon of large strongly ossified anuran is represented in the assemblage.

To date, 27 Mahajanga Basin Project localities have yielded specimens of *Beelzebufo*. Locality MAD93-35 (a rich microvertebrate site subjected to intensive wet and dry screening) is of particular note because it has yielded a large number of isolated specimens (19) of *Beelzebufo*, collected during eight of the 11 expeditions. Similarly, locality MAD98-25 (Fig. 7), discovered five years after locality MAD93-35, yielded only isolated elements of *Beelzebufo*, or fragments thereof, most of which were collected in 1998. Although we considered it likely that most of the elements recovered from MAD98-25 were derived from the same individual [26], these, and a few additional fragments collected in 1999 and 2007, were conservatively catalogued as isolated specimens. However, the partial associated cranium and vertebrae recovered in 2010 and 2011 came from the same locality. Several factors now allow us to conclude more definitively that all of the material recovered from MAD98-25 belongs to the same individual of *B. ampinga*:

1. Anuran fossils are comparatively rare in the Maevarano Formation.
2. The area of MAD98-25 excavated, as well as the deflation pocket stratigraphically below it and from which material was collected as float, measures only ∼25 m^2^.
3. The specimens were all found within or weathered from a single Facies 2 bed.
4. The in situ material discovered in 2010 consisted of two main clusters of articulated elements and several intervening fragments along a linear trajectory trending from southeast to northwest (∼318°). The two clusters were separated by 2.8 m. The close association and linear arrangement of partially disarticulated skulls and skeletons, whether large or small, are typical of the massive (i.e., non-stratified) Facies 2 deposits of the Anembalemba Member, and are thought to be the result of the limited transport potential of debris flows [43]. The SE–NW trend of the elements is consistent with directional trends measured in the stratified Facies 1 units of the Anembalemba Member (vector mean = 337° derived from 51 measurements in the Berivotra Study Area; [42]).
5. The various elements and fragments are all of a size consistent with being derived from the same individual.
6. The colour and quality of preservation of the various elements and fragments are similar (except for those that had obviously lain exposed at the surface for some time).
7. Most significantly, there is no duplication of elements. The elements recovered in situ at MAD98-25 in 2010 are from the median and right portions of the cranium whereas the isolated elements recovered as float prior to 2010 are mainly from the left side (Figs 4–5).

All of the anuran material from MAD98-25 is now therefore catalogued within a single museum number, FMNH PR 2512 (Table S1 in File S1). Unfortunately, complete excavation of the site in 2011 and careful dry and wet screening of the quarried matrix yielded only a few more cranial and vertebral fragments.

### Permits

All collecting and exportation permits were issued to the Research Foundation of the State University of New York and the Département de Paléontologie et Anthropologie Biologique, Faculté des Sciences, Université d'Antananarivo and provided by the Ministère des Mines et des Hydrocarbure and the Ministére de l'Enseignement Supérieur et de la Recherche Scientifique of the Republic of Madagascar. All necessary permits were obtained for the described study, which complied with all relevant regulations.

### Institutional Abbreviations

FMNH, The Field Museum, Chicago, Illinois, U. S. A.; LACM, Natural History Museum of Los Angeles County, U.S.A.; UA, Université d'Antananarivo, Antananarivo, Madagascar.

## Methods

### CT scanning

Most specimens of *Beelzebufo* were batch-scanned on a vivaCT 75 scanner (Scanco Medical AG, Brüttisellen, Switzerland); the braincases of FMNH PR 2512 and UA 9675 and several smaller specimens were scanned on a μCT 40 scanner (Scanco Medical AG, Brüttisellen, Switzerland). Both machines are managed by the Stony Brook University Department of Biomedical Engineering.

Sub-volumes of individual specimens were extracted as tiff or dicom files using Avizo 7.0–7.1 (Visualization Sciences Group) and ImageJ (U.S. National Institutes of Health). These data volumes were employed to generate surfaces used as digital models, both for this study and for general documentation and curation of data in ongoing efforts undertaken by the Mahajanga Basin Project. μCT datasets range in voxel size from 40–16 µm^3^, and were typically scanned at 70 kV and 114 µA (details of scan parameters for particular specimens are available upon request). Scans of the skeleton of a large female *Ceratophrys aurita* (LACM 163430 [catalogued as *C. varia*]) used to construct the three-dimensional digital model of the postcranium, articulated polyester casts of the posterior region of the skull of FMNH PR 2512, and casts and specimens of larger comparative materials not used for figures, were conducted on a GE Lightspeed 64-source medical CT scanner at 140 kVp and 250 µA, 0.0625 mm z-slice spacing (interpolated from an effective z-slice reconstruction of 0.625 mm^3^). The machine is managed by the Stony Brook University Department of Radiology. Field blocks containing associated materials of FMNH PR 2512 were also scanned on this machine prior to preparation to document completeness and associations, in general keeping with specimen preparation and curation protocols of the Mahajanga Basin Project. Table S2 in File S1 lists the specimens of *Beelzebufo* used in the digital reconstructions.

### Specimen digital model surfaces and figure images

Avizo surface files were used both for the three-dimensional skeletal reconstruction and figure images. Surface files were extracted from isosurface renderings of μCT datasets in Avizo (6.3.1–7.1), and their ultimate surface view draw styles visualized with shaded, opaque, vertex normal, non-specular, constant-colour neutral gray attributes, except in cases where surface triangles were additionally coloured dark grey, dark blue, or light blue to highlight relationships between surfaces.

Polygon mesh editing associated with the skeletal reconstruction, including transformations, translations, scaling, mirror-imaging, and compositing, was performed in Avizo (6.3.1–7.1) and is described in detail in Section A of File S1; those surfaces imaged for descriptive figuring underwent no mesh editing. Although a skeleton of *Ceratophrys aurita* (LACM 163430) was used to provide a template for the reconstruction of the postcranium and jaws of *Beelzebufo*, the reconstruction of the cranium was based on the fossil materials alone, using FMNH PR 2512 supplemented by specimens from other localities (Fig. 1). The fit of the occipital condyles to the cranial surface of the atlas ensured that the proportions of the head relative to the body were correct.

The majority of images of morphology are screen-captures of the high-resolution polygon meshes generated from μCT datasets. This allowed for standardization of surface appearances and comparative ease of positioning for morphological documentation. All imaging work was conducted using Avizo 7.1. Images were captured in orthographic view and with default headlight, using the snapshot function, and 5×5 tiles exported as tiff files. The braincase specimen UA 9675 was also imaged in Avizo 7.1 but the surface file visualization was set to specular (inset) and transparent (main image) in order to visualize the labelled voxels within the specimen model. μCT slice images of the braincase were generated by taking screen shots of thresholded images visualized in ImageJ. Images of the sculpture, cranial bones, and possible osteoderms were created using traditional digital photography.

### Digital segmentation

Digital segmentation was performed on the μCT dataset of UA 9675 in order to label voxels corresponding to volumes within the occipital canal and inner ear. Additional, unpublished segmentations of internal structures within maxillary specimens of *Beelzebufo* (particularly neurovascular canals) were also performed to provide corroboration with external landmarks in constructing a composite maxilla. All segmentation was done using Avizo 7.1 (Visualization Sciences Group) except for that for the occipital canal and inner ear of UA 9675, which was accomplished with Avizo 6.3.1 (Visualization Sciences Group).

### Terminology

The anatomical terminology used in the descriptions of individual elements primarily follows that of Lynch, Trueb and Wild [59]–[61]. The phylogenetic terminology is mainly that of Pyron and Wiens [27]. Note that the clade name Ceratophryidae has been used with variable levels of inclusiveness by recent authors. The extant genera *Ceratophrys*, *Lepidobatrachus*, and *Chacophrys* always form the core group; we follow [27] in restricting Ceratophryidae to the clade encompassing the last common ancestor of *Ceratophrys*, *Lepidobatrachus*, and *Chacophrys*, and all (but only) taxa descended from that ancestor. It is thus directly equivalent to Ceratophryinae as used by Ruane et al. [29]. Frost et al. [9] included *Telmatobius* and the batrachylids *Atelognathus* and *Batrachyla* within Ceratophryidae. Roelants et al. [62] followed them but excluded batrachylids. Irisarri et al. [63] used *Telmatobius* to represent ceratophryids in their analysis and estimation of divergence dates, but did not test this by including members of the core group. Given that the placement of *Telmatobius* has not been consistent in recent phylogenetic analyses, we consider it preferable to treat it as a separate taxonomic unit.

### Systematic palaeontology

Anura Fischer von Waldheim, 1813 [64]

Neobatrachia Reig 1958 [65]

Hyloidea sensu Pyron and Wiens 2011 [27]

*Beelzebufo ampinga* Evans et al. 2008 [26]

### Type specimen

UA 9600, atlas vertebra ( = cervical [59]) fused to second presacral vertebra.

### Type locality and horizon

Locality MAD93-25, Berivotra Study Area, Anembalemba Member, Maevarano Formation, Mahajanga Basin, northwestern Madagascar (Figs. 6–7); locality coordinates on file at Stony Brook University, The Field Museum, and the University of Antananarivo.

### Age and distribution

Known only from the Late Cretaceous (Maastrichtian) of northwestern Madagascar, in the Berivotra and Lac Kinkony field areas (Fig. 6), Maevarano Formation, Mahajanga Basin, northwestern Madagascar. Most specimens are from the Anembalemba Member, but a few are from the underlying Masorobe Member and a small subset was recovered from the overlying Lac Kinkony Member in the Lac Kinkony Study Area. Locality coordinates on file at Stony Brook University, The Field Museum, and the University of Antananarivo.

### Referred specimens and localities

See Table S1 in File S1 for changes from [26]—Locality MAD93-01: UA 9614 – posteroventral process of right squamosal; UA 9615 – cranial fragment from antorbital margin, either nasal or frontoparietal; FMNH PR 2003 – right half of sacral vertebra. Locality MAD93-06: UA 9618 – fragment of left quadratojugal; UA 9619 – vertebral spine table. Locality MAD93-14: UA 9620 – fragment of?dorsal bony plate. Locality MAD93-17: FMNH PR 2498 – cranial fragment; FMNH PR 2536 – right fused squamosal-quadratojugal flange; FMNH PR 2537 – squamosal fragment. Locality MAD93-25: UA 9945 – maxilla fragment; UA 9946 – fragment of right squamosal, including suture with frontoparietal. Locality MAD93-33: FMNH PR 2497 – cranial or vertebral spine table fragment; UA 8677 – partial right angulosplenial. Locality MAD93-34: UA 9631 – fragment of right squamosal, including suture with frontoparietal; UA 9632 – cranial or vertebral spine table fragment; FMNH PR 2499 – partial right maxilla; FMNH PR 2500 – partial posterior process of right quadratojugal; FMNH PR 2501 – partial right quadratojugal. Locality MAD93-35: UA 9623 – fragment of otic and ventral processes of squamosal; UA 9624 – posterior process of left quadratojugal; UA 9625 – posteroventral process of right squamosal; UA 9626 – cranial fragment; UA 9627 – partial vertebral spine table; UA 9635 – anterior fragment of right maxilla; UA 9676 – fragment of right maxilla bearing maxillary nerve canal; UA 9677 – fragment of frontoparietal or squamosal; UA 9679 – cranial fragment; UA 9947 – presacral vertebra, interpreted as PS3; UA 9948 – posterior presacral vertebra; FMNH PR 1960 – partial right premaxilla; FMNH PR 2504 – vertebral centrum and partial neural arch; FMNH PR 2505 – facial process of right maxilla; FMNH PR 2506 – partial right maxilla; FMNH PR 2507 – facial process and pars dentalis of right maxilla; FMNH PR 2508 – cranial fragment from antorbital margin, frontoparietal or nasal; FMNH PR 2509 – conjoined midline frontoparietal fragment; FMNH PR 2510 – partial left maxilla. Locality MAD93-36: UA 9678 – vertebral spine table; UA 9949 – fragment of otic plate of right squamosal. Locality MAD93-37: FMNH PR 1959 – partial right quadratojugal; UA 9621 – anterior fragment of right quadratojugal. Locality MAD93-52: UA 9950 – fragment of otic and ventral processes of squamosal; UA 9951 – left nasal fragment. Locality MAD93-73: UA 9622 – partial left premaxilla. Locality MAD96-21: UA 9628 – right tibiofibula. Locality MAD96-24: UA 9629 – four fragments of large left squamosal. Locality MAD98-25: FMNH PR 2512 – partial skull and axial column, including braincase, partial frontoparietal, and right posterior skull, portions of the left posterior skull and rostrum, left pars facialis of maxilla, partial stapes, atlas and second presacral vertebra, presacral vertebra interpreted as PS4, presacral spinous process interpreted as PS5, partial presacral vertebral centrum, partial anterior urostyle. Locality MAD99-14: UA 9633 – right frontoparietal or nasal. Locality MAD99-29: UA 9634 – partial right maxilla. Locality MAD01-15: UA 9952 – cranial or vertebral spine table fragment. Locality MAD03-05: UA 9636 – partial urostyle; UA 9637 – cranial or vertebral spine table fragment. Locality MAD03-10: UA 9638 – otic plate of right squamosal. Locality MAD03-18: UA 9617 – posteriormost tip of left quadratojugal; UA 9640 – fragment of left frontoparietal and otoccipital. Locality MAD05-28: UA 9639 – midportion of left quadratojugal. Locality MAD05-64: UA 9675 – partial left frontoparietal and otoccipital. Locality MAD07-15: UA 9674 – posterior process of left quadratojugal. Locality MAD07-20: UA 9953 – partial maxilla. Locality MAD10-13: UA 9954 – presacral vertebral centrum; UA 9955 - two cranial fragments. Locality MAD10-24: UA 9957 – right tibiale-fibulare; UA 9958 – left nasal fragment.

### Diagnosis

Revised from [26]—Large (adult posterior skull width ∼129–154 mm), hyperossified anuran with external skull roofing bones having coarse pit-and-ridge sculpture; differs from all known anurans, living and extinct, in the possession of long squamosal-quadratojugal flanges that extend posterolateral to the jaw joints (Fig. 3) combined with procoelous anterior vertebrae having tall neural spines with bilaterally expanded spine tables bearing sculpture matching that of the skull (Fig. 1).

### Description - Skull

#### General features of the skull

As reconstructed (Figs. 3–4), the skull of the *Beelzebufo* individual represented by FMNH PR 2512 was strongly built, posteriorly deep, short (83.3 mm), and wide at the level of the jaw joints (106.3 mm bi-quadrate width; 128.7 mm greatest width). The quadrates lay at or just anterior to the level of the occipital condyles, but the skull was extended bilaterally by large squamosal-quadratojugal flanges that rendered the posterior margin of the skull distinctly U-shaped. The dermal roofing bones are heavily exostosed (sensu [60]) with a coarse pit-and-ridge sculpture pattern (Fig. 8A), and are thick (Fig. 8B–C). The large squamosal met the maxilla, quadratojugal, crista parotica, and frontoparietal, and there was a complete maxillary-quadratojugal arcade (as reconstructed in Fig. 4B). A large temporal fenestra was bordered by the maxilla, quadratojugal, and squamosal.

The following description and the reconstructions in Figs 3–4 are based mainly on FMNH PR 2512 (Figs 9–11), which comprises a dorsoventrally compressed braincase with associated frontoparietal dorsally and parasphenoid ventrally; a nearly complete right squamosal, quadratojugal, and quadrate; and most of the right pterygoid (also rather crushed). Part of a right frontoparietal was collected in association with FMNH PR 2512 and can be fitted against it. Its correct placement is confirmed by a partial matrix impression of the skull roof recovered with the specimens. The description is supplemented with information from several partial maxillae and premaxillae, an angulosplenial, and useful portions of other bones representing parts that are missing or damaged in FMNH PR 2512.

#### Premaxilla

**Figure 12:**
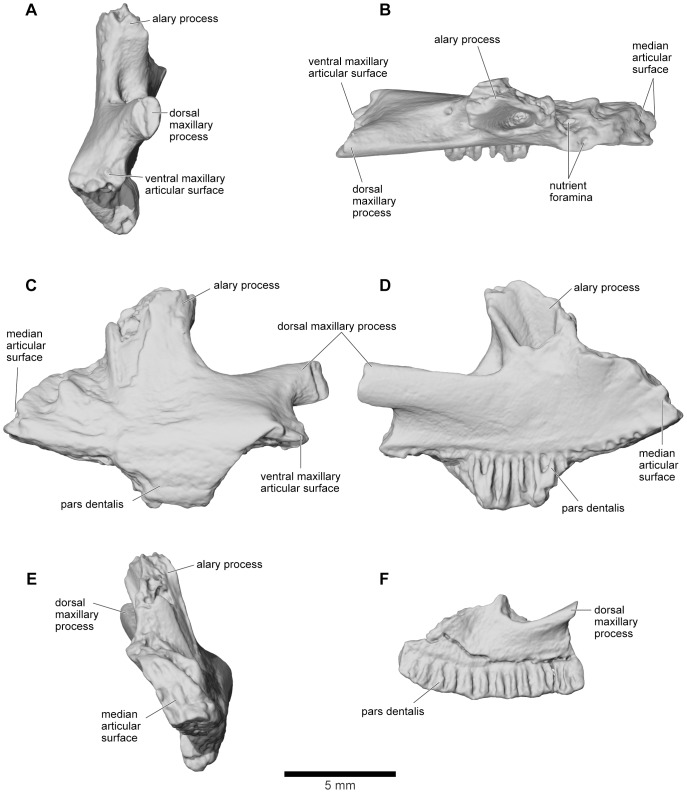
Premaxilla. **A**, distal (lateral); **B**, dorsal; **C**, labial; **D**, lingual; and **E**, mesial (medial) views of left premaxilla, UA 9622. **F**, lingual view of right premaxilla, FMNH PR 1960.

The paired premaxillae are represented by two incomplete specimens: UA 9622, from the left side (Fig. 12 A–E), and a much smaller right bone, FMNH PR1960 (Fig. 12F), described and figured by Asher and Krause ([25]: fig. 1L, M). Neither is exostosed. The medial edge bears a rugose articular surface for the contralateral premaxilla, whereas the more complex shape of the posterolateral border corresponds to that of the anteromedial end of the maxilla. Posterodorsally, UA 9622 extends into a stout, hemicylindrical alary process (pars alaris) that is broken distally. Around its base are numerous nutrient foramina. The pars dentalis (alveolar margin) was slightly overlapped by the pars dentalis of the maxilla (as shown by reciprocal facets), but also abutted it ventrally (as shown by a small thickened articular surface on the posterolateral edge of UA 9622: Fig. 12A). Farther dorsally, the premaxilla is drawn into a process (the base of which is preserved in UA 9622) that extended posteriorly and lay in a groove along the anterior process of the maxilla so that the two bones had a strong, interlocking articulation. FMNH PR 1960 does not preserve the alary process but bears an almost complete pars dentalis, with 13–14 tooth positions. The premaxilla of *Beelzebufo* is distinctive in lacking any development of a palatine shelf (pars palatina) (Fig. 12D,F).

#### Maxilla

**Figure 13:**
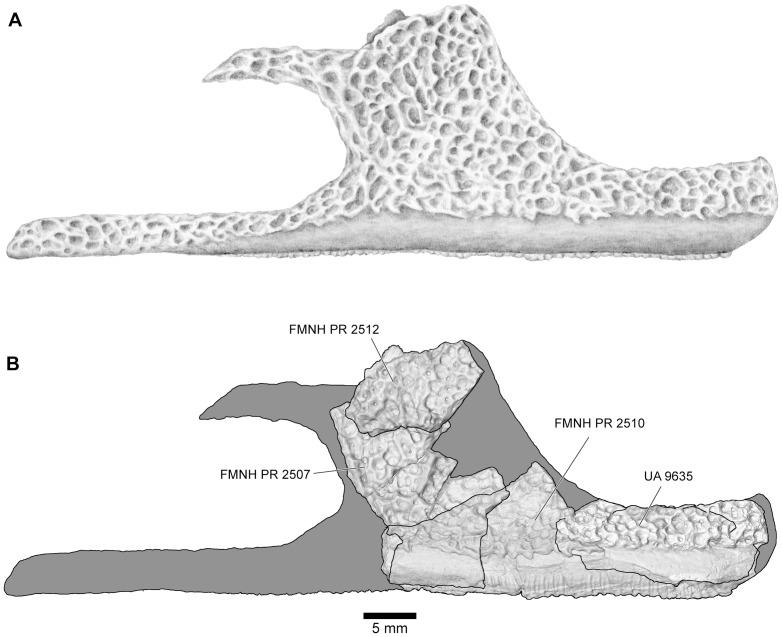
Reconstructions of right maxilla in labial view. **A**, illustrated reconstruction based on composite digital model; **B**, outline reconstruction showing main specimens (FMNH PR 2507, FMNH PR 2510 [reversed], FMNH PR 2512 [reversed], UA 9635) in combined digital model. Additional data taken from neighbouring elements and positional information in skull reconstruction. See Figs 14, 15 for detailed views of individual specimens.

**Figure 14:**
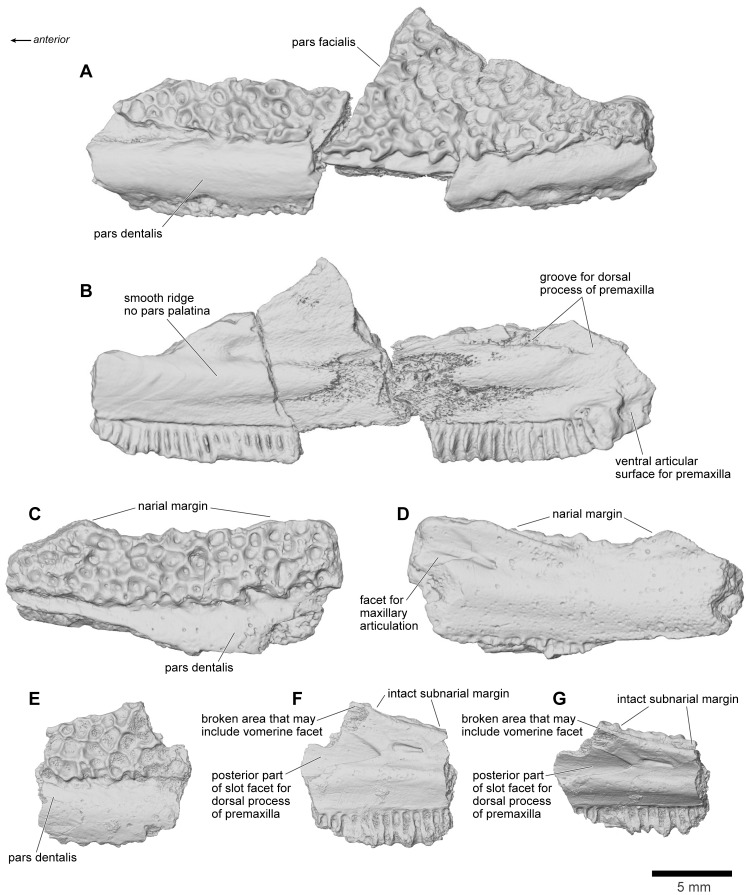
Maxilla. **A**, labial; and **B**, lingual views of left maxilla, FMNH PR 2510. **C**, labial; and **D**, lingual views of right maxilla, UA 9635. **E**, labial; **F**, lingual; and **G**, dorsolingual views of right maxilla, FMNH PR 2499.

**Figure 15:**
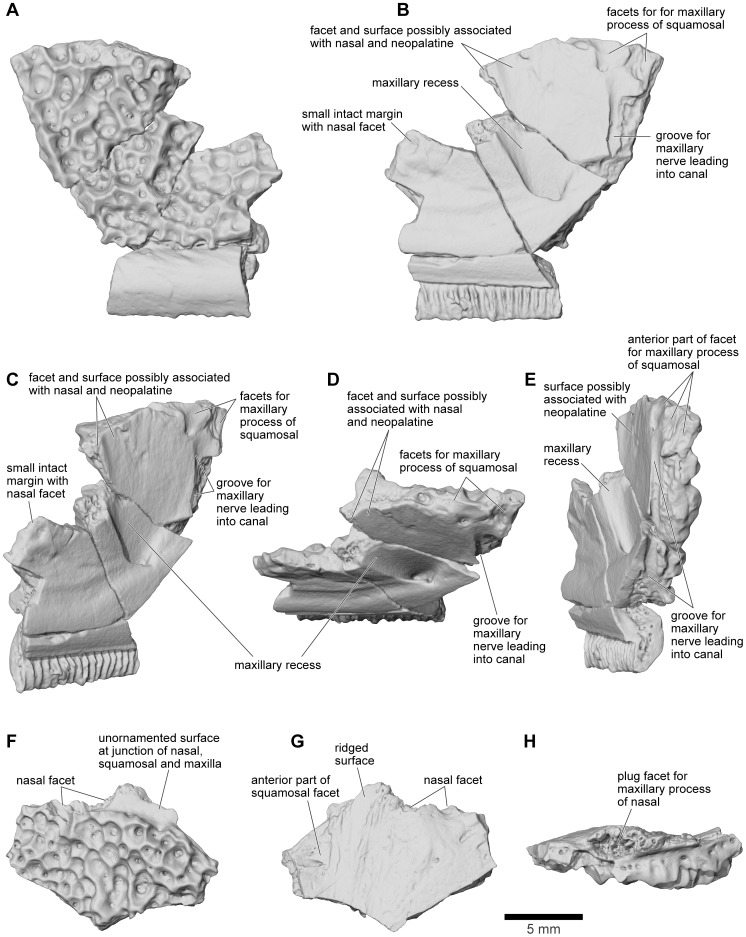
Maxilla. **A**, labial; **B**, lingual; **C**, oblique lingual; **D**, dorsolingual; and **E**, posterolingual views of partial right maxilla that includes part of the pars facialis, FMNH PR 2507. **F**, labial; **G**, lingual; and **H**, dorsal views of fragmentary pars facialis of left maxilla, FMNH PR 2512.

The maxilla is well represented by several isolated fragmentary specimens preserving parts of the pars dentalis and/or the pars facialis (FMNH PR nos. 2499, 2505–2507, 2510, 2512; UA nos. 9634, 9635, 9676, 9945, 9953: Figs 13–15), but the most representative of the former is FMNH PR 2510 and, of the latter, FMNH PR 2507. None, however, is complete and none preserves the articular surfaces with the nasal or quadratojugal. The most complete maxillary specimen overall is FMNH PR 2510 (Fig. 14A–B), the anteroventral part of a left bone preserving much of the pars dentalis, a small portion of the pars facialis, and the articulation with the premaxilla. The external surface of the pars facialis (Fig. 14A) is exostosed dorsally but that of the pars dentalis is smooth. However, as shown by FMNH PR 2510, the exostosis extends farther ventrally at the posterior end than it does rostrally. Medially (Fig. 14B), the anterior tip bears a pocket-like facet for the reception of the pars dentalis of the premaxilla (shown well by FMNH PR 2499, Fig. 14F–G) and a slot facet for the prong-like dorsal process of that bone (also preserved in UA 9634 and UA 9635 [Fig. 14D]). Above the pars dentalis, the bone is smooth and lacks any medial development of the pars palatina. In FMNH PR 2510, most of the pars facialis is broken away with the exception of a small part of the ventral narial margin. Other specimens (e.g., UA 9635, FMNH PR 2505) supplement it. UA 9635 (Fig. 14C–D) is particularly useful in that it preserves the long narial margin and shows that the maxilla was shallow ventral to the nasal (unlike the deep flange present here in extant ceratophryids). Anterior to the naris, the maxilla bears a slight dorsal expansion (as shown by FMNH PR 2499, Fig. 14E–F) that may have met the vomer medially (Fig. 14F). Judging from FMNH PR 2507 (Fig. 15A–E), the postnarial pars facialis was erect and quite deep. The pars facialis was apparently drawn into an anterodorsal nasal process (part of which is preserved on FMNH PR 2512 and includes a plug facet that would have strengthened the joint: Fig. 15H). More posteriorly, the pars facialis also met the squamosal (FMNH PR 2507) and, from the reciprocal facets on that bone, would have tapered posterodorsally. Only the anterior tips of that articular surface are preserved on FMNH PR 2507 (Fig. 15B–D). Ventral to the squamosal facet, FMNH PR 2507 bears a distinct medial groove that runs downward and forward from the posterodorsal edge. The groove then canalizes the pars facialis and emerges onto the ventrolateral surface below, at the junction of the pars facialis and pars dentalis (best preserved on FMNH PR 2506), roughly in line with the medial maxillary recess. By comparison with living taxa, this groove marks the position of a canal carrying sensory branches of the maxillary nerve forward (through the layer of exostosis) onto the external surface of the pars dentalis and probably also dorsally into the tissues lining the orbit. This canal would have had its entrance in the anteroventral border of the temporal fossa, but the posterior edge of the bone (including that part meeting the pterygoid) is broken off. The preserved posterodorsal edge of the maxillary recess bears a small facet flanked by a low straight ridge; behind this is a distinct surface, slightly concave and weakly ridged. These features may be associated with the attachments of the nasal and neopalatine, the latter sheathing the planum antorbitale of the chondrocranium medially. FMNH PR 2512 (Fig. 15F–H) includes a fragment of the pars facialis bearing an interlocking facet that may have contacted the nasal. In this slightly larger specimen, the possible neopalatine surface noted above is more strongly ridged. Externally, the junctions of the maxilla with the squamosal and nasal are marked by a smooth area of pars facialis lacking exostosis. Given the proximity of the nasal and squamosal facets on the FMNH PR 2512 fragment, we infer that the squamosal and nasal approached one another in the ventral orbital margin to exclude, or nearly exclude, the maxilla. In FMNH PR 2510 (Fig. 14B), the recesses for the teeth decrease in height, as well as mesiodistal length, toward the posterior end, but no specimen preserves the posterior end of the tooth row.

Together the available specimens show that the maxilla was large and formed much of the anterolateral wall of the skull. It had a strong interlocking anterior suture with the premaxilla, a pars facialis that was long and low below the nasal aperture but taller posteriorly, and sutural contacts anterodorsally with the nasal and posterodorsally with the squamosal. These contacts excluded, or nearly excluded, the maxilla from the orbital margin. The maxilla also contacted the quadratojugal (see below).

#### Nasal

**Figure 16:**
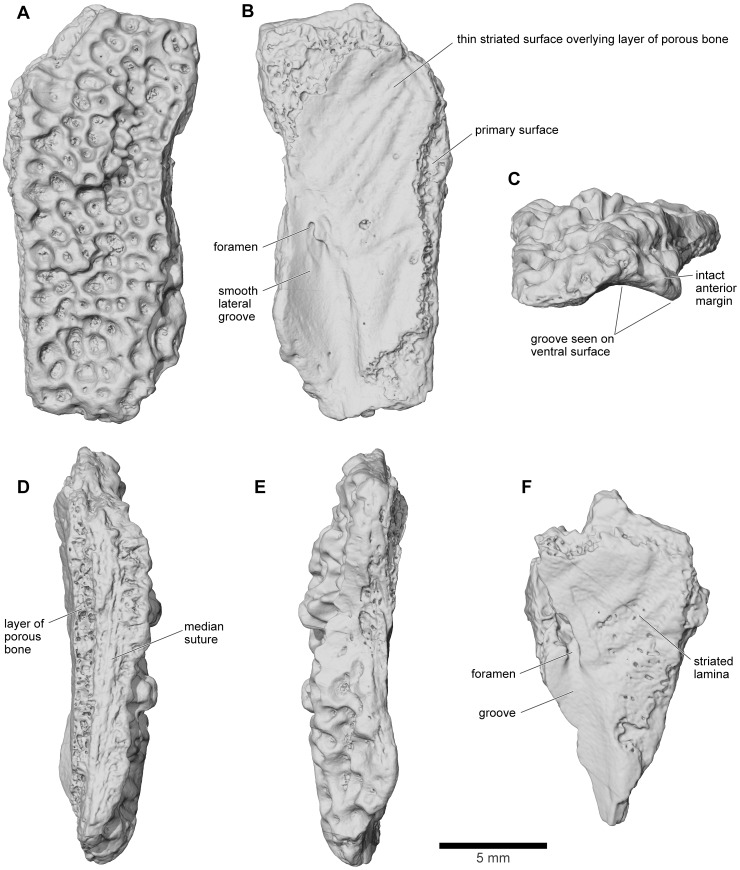
Anterior portion of left nasal. **A**, dorsal; **B**, ventral; **C**, anterior; **D**, medial; and **E**, lateral views, UA 9951; **F**, ventral view, UA 9958.

In the original description [26], the peculiar posterolateral flanges of the quadratojugal were interpreted as nasals, which have a similar shape in other anurans. Our reanalysis has identified two specimens that are probably nasals, UA 9951 and UA 9958. Both specimens are from the left side of the skull and represent the anteromedial roofing part of the bone; no specimen can be attributed with confidence to the ventrolateral maxillary process. The more complete specimen is UA 9951 (Fig. 16A–E), which preserves part of the median suture and a straight anterior margin. The lateral edge is broken but preserves the dorsal part of a curving anteroventral flange that is separated from the dorsal body by a ridge-like anteroposterior thickening of the exostosis. The ventral surface (Fig. 16B) is divided into two distinct parts. Anterolaterally, the bone is smooth and forms a concave channel that is pierced posteriorly by a small neurovascular foramen. However, medially and posteriorly, the originally smooth surface is covered by a thin layer of more porous bone that may represent ossification into tissues lining the nasal cavity. This is supported by the presence of what appear to be blood vessel grooves across its surface. UA 9958 is less complete, but shows the same features of the ventral surface as UA 9951 (Fig. 16F).

**Figure 17:**
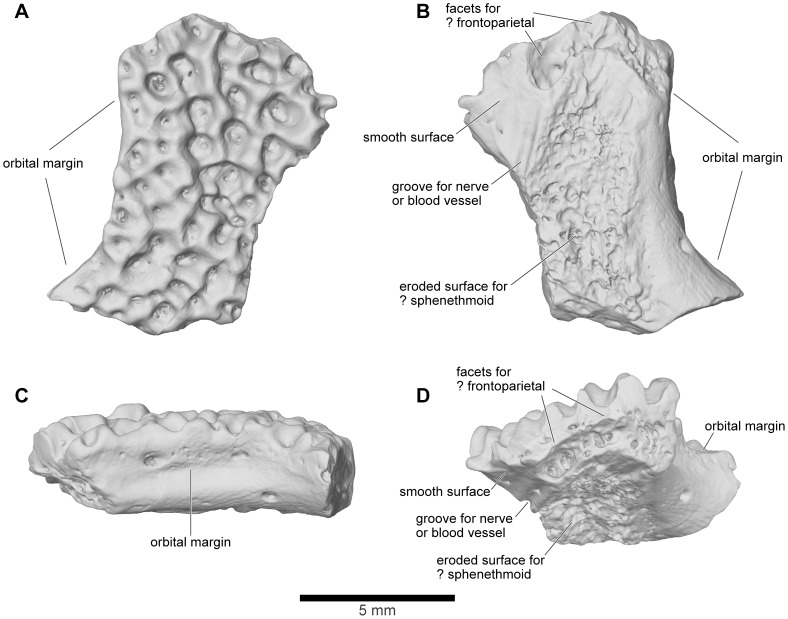
Posterior portion of right nasal (or anterior portion of left frontoparietal), UA 9615. **A**, dorsal; **B**, ventral; **C**, lateral; and **D**, oblique ventral views. Specimen positioned as posterior portion of right nasal for cranial reconstruction in Figs. 1–5.

Neither UA 9951 nor UA 9958 preserves any trace of facets for either the maxilla or frontoparietal. However, UA 9615 is a small cranial fragment (Fig. 17) with a thick outer edge that formed part of the antorbital rim. It could be a posterior fragment of the nasal or an anterior fragment of the frontoparietal. In Figs. 1–5, it has been positioned, without contacts, as a posterior part of the right nasal, with a facet on its posteroventral edge for the frontoparietal. The dorsal surface is exostosed but the ventrolateral surface is eroded and gives the impression that another bone has been stripped from its surface, possibly the sphenethmoid. This rough surface is flanked medially by a smooth surface, separated from the roughened region by a narrow groove for a nerve, blood vessel, or both.

#### Frontoparietal

FMNH PR 2512 preserves the posterior parts of the fused frontoparietals in articulation with the braincase, the right squamosal, and a small section around the midline (suture closed; Fig. 9). In addition, two parts of the orbital margin, from both the left and right sides, are associated with this main piece. An impression preserved with the braincase region of FMNH PR 2512 shows the original position of the right orbital portion and this can be attached to the main piece between the anterior edge of the braincase section and the parietal shelf. A further impression completes the frontoparietal component of the orbital rim. Additional isolated fragments of the frontoparietal from other localities include UA 9640 and UA 9675, each of which comprises parts of a left frontoparietal and otoccipital.

The relative completeness of FMNH PR 2512 allows us to restore the shape of the frontoparietal, at least in its posterior and central sections, with confidence (Figs. 1–5). The combined frontoparietals formed a parallel-sided plate between the orbits, expanding posteriorly into a shelf that met the squamosal in a partly scarfed, partly interdigitated joint. Together, the frontoparietal and squamosal formed the posterior margins of the dorsally positioned orbit. The ventromedial surface of the parietosquamosal shelf is smooth and roofed a small sub-temporal fossa (sensu [59]), but this was limited laterally by a contact between the crista parotica of the otic capsule and the shelf, immediately below the squamosal-parietal suture (FMNH PR 2512). However, in contrast to the original reconstruction ([26]: fig. 2A), the posterior margin of the parietosquamosal shelf was not embayed. Posteromedially, the frontoparietal overlay the otoccipitals (sensu [59], fused prootic+exoccipital) and was fused on either side to them, partially roofing the braincase in the anterior midline where the prootics fail to meet (or remained cartilaginous judging from the pitted medial edges). Bilateral occipital canals are fully roofed by the frontoparietal, opening anteriorly in the posterior walls of the orbits and posteriorly onto the occipital surface. By comparison with living genera, these canals carried occipital arteries (branches of the occipito-vertebralis artery, not the carotid artery as suggested by some authors, e.g., [59]). Traces of the ventral suture between the frontoparietal and braincase are preserved in UA 9675 and show that the frontoparietal was extended ventrally by a lamina perpendicularis that contributed to the lateral wall of the braincase. Posterolaterally, a small unornamented flange extended toward the epiotic process of the braincase on each side; in the midline, a thick unornamented column extends ventrally to meet the roof of the foramen magnum, and contributes to the formation of deep recesses on the occipital surface.

#### Squamosal

**Figure 18:**
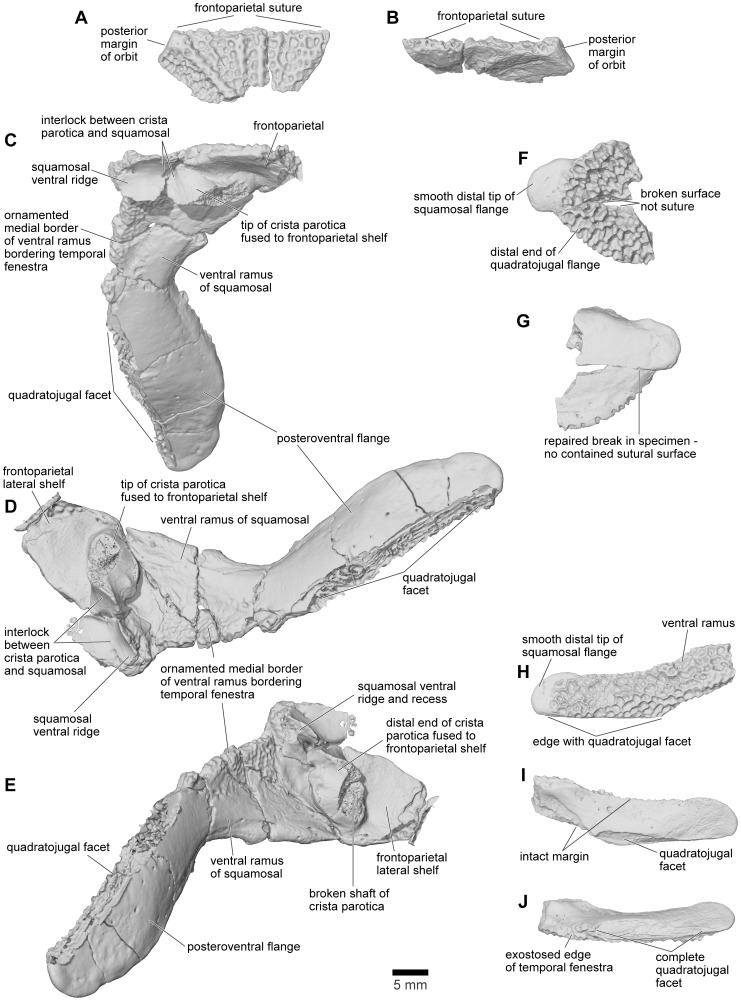
Squamosal. **A**, dorsal; and **B**, ventromedial views of otic plate of left squamosal, FMNH PR 2512. **C**, ventromedial; **D**, ventral; and **E**, anteroventral views of right squamosal in articulation with lateral shelf of frontoparietal, FMNH PR 2512. **F**, dorsolateral; and **G**, ventromedial views of postquadrate flange formed from fused right squamosal and quadratojugal, FMNH PR 2536. **H**, lateral; **I**, ventromedial; and **J**, ventral views of part of small right squamosal, UA 9625.

Both the left and right squamosal bones are preserved in FMNH PR 2512. The left (Fig. 18A–B) preserves the facet for the frontoparietal in the parieto-squamosal bridge, whereas the right (Figs. 10, 18C–E) is virtually complete and preserves the otic plate, the anterior (zygomatic) process, the ventral ramus, and the posteroventral flange. A number of isolated fragmentary specimens can also be identified as parts of the squamosal, including: FMNH PR 2512 (additional fragments not listed above), FMNH PR 2536 (Fig. 18F–G), FMNH PR 2537, UA 9614, UA 9623, UA 9625 (Fig. 18H–J), UA 9629, UA 9631, UA 9638, UA 9946, UA 9949, and UA 9950. Several of these specimens were previously identified as belonging to the nasal ([26]:fig. 3F,G) because the long, flat suture with the quadratojugal resembles a midline internasal suture.

The squamosal of *Beelzebufo* has a unique and complex shape. In most anurans, the squamosal has only three rami: an anterior (or zygomatic) process that is usually short but may meet the maxilla in well ossified taxa, a posterior otic process that overhangs the ear region and supports the tympanic membrane, and a ventral process that meets the pterygoid, sheathes the palatoquadrate, and may also contact the quadratojugal. Medially, the anuran squamosal is usually separated from the frontoparietal but some extant anurans (e.g., some *Pelobates*, Bufonidae, Ceratophryidae, *Calyptocephalella*, *Triprion*) develop a parietosquamosal contact. This can be broad (*Calyptocephalella*, *Lepidobatrachus*, *Triprion*, some bufonids and pelobatids), narrow and anterior (e.g., *Ceratophrys*), or narrow and posterior (e.g., some bufonids). The squamosal of *Beelzebufo* resembles that of other heavily ossified anurans in having had accessory contacts (maxilla, quadratojugal, frontoparietal), but there is no posterodorsal otic process nor any embayment of the posterior margin that could have held a tympanic membrane. There is, however, an additional posteroventral flange.

The dorsomedial edge of the squamosal met the frontoparietal shelf in an interdigitated joint (Fig. 18B–C), braced from below by the crista parotica (Fig. 18C–E). On the right squamosal, the wide distal end of the crista parotica is partially fused to the edge of the frontoparietal but, just lateral to this articulation, the otic plate of the squamosal bears a ventral ridge and recess arrangement that creates an interlocking joint for the lateral tip of the crista parotica (Fig. 18C–E). Farther anterodorsally, the squamosal extends into a curved, but mainly horizontal, zygomatic process that formed the posterolateral margin of the orbit (Figs 3–4). Along its anteroventral margin, and wrapping around on to the medial surface, this process bears an articular surface for the pars facialis of the maxilla. A deep recess at the posterior end of the facet would have received a reciprocal process from the maxilla, helping to lock the joint, but neither the anterior tip of the squamosal nor the posterior tip of the maxillary pars facialis are preserved.

**Figure 19:**
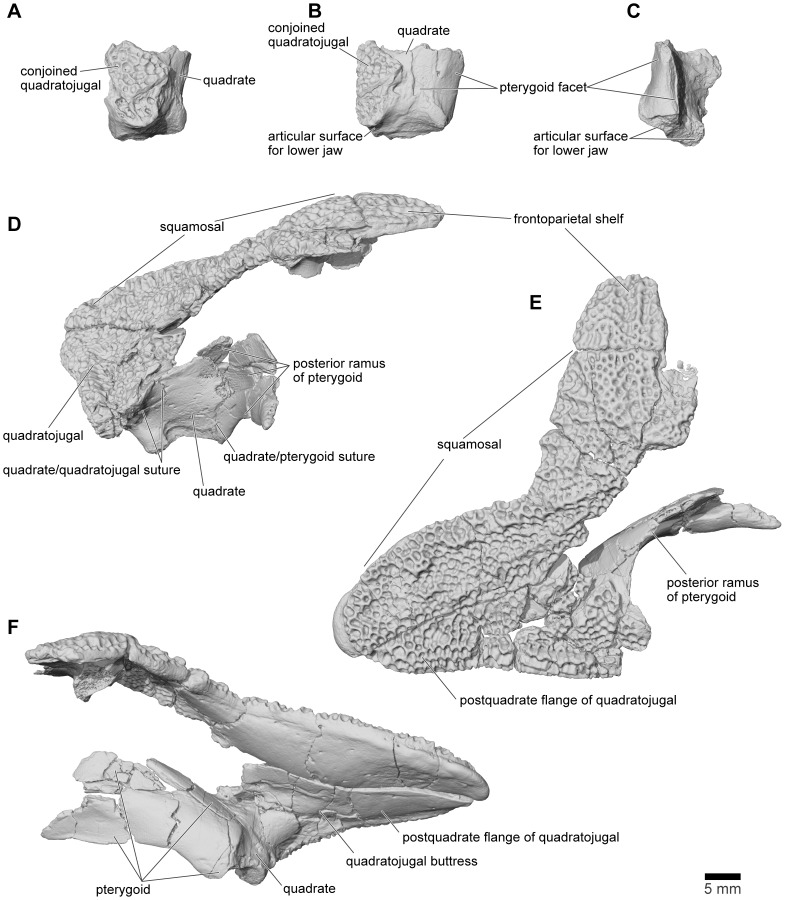
Quadratojugal/quadrate, FMNH PR 2512. **A**, lateral; **B**, posterior; and **C**, medial views of left quadrate with part of conjoined quadratojugal. **D**, anterior; **E**, dorsolateral; and **F**, posteroventromedial views of right quadrate, quadratojugal, and pterygoid.

Posteroventrally, the squamosal is drawn out into a broad, flat, fully exostosed ventral process descending at about 58° to the horizontal. The process is well preserved in FMNH PR 2512 (Figs 10, 18) and in UA 9625 (Fig. 18H–J), a much smaller squamosal. The right quadrate in FMNH PR 2512 is articulated with the pterygoid and quadratojugal, the latter extending a thin smooth, anterolateral lamina that has a long articulation with the pterygoid medially (Fig. 10). The left quadrate of FMNH PR 2512 has a distinct facet in this position (Fig. 19B–C). By comparison with modern anurans, this region should then be invested by the ventral ramus of the squamosal. However, although the anterior margin of the squamosal ventral ramus is slightly broken in FMNH PR 2512, the intact margins of UA 9625 bear no traces of a sutural contact for the quadrate. There is only the long ventral articular surface for the posterior flange of the quadratojugal (Fig. 18H–J). Thus, relative to other anurans, the hypertrophied quadratojugal seems to have provided the sole support for the quadrate. The squamosal of *Beelzebufo*, despite is large size, appears neither to meet the quadrate nor the pterygoid (Fig. 19D–F). The posteromedial surface of the ventral ramus is smooth (Fig. 18C–D) but the anteromedial surface is weakly sculptured (Fig. 18C,J), possibly reflecting adductor muscle origin. The anterior edge of the ventral ramus entered the margin of the large temporal fenestra, which, given the angulation of the ventral ramus of the squamosal, would have been subtriangular in shape. In living hyperossified anurans like *Pyxicephalus, Ceratophrys*, and *Osteopilus* (SEE, pers. obs), this fenestra is covered by fascia and the chamber beneath it is filled by adductor muscles. In *Pyxicephalus* and *Osteopilus*, part of the adductor mandibulae longus [66] originates from the occipital surface and curves over the surface of the crista parotica before passing ventrally to the jaw (SEE pers. obs.). This would increase the fibre length and potential extension of the muscles. In *Ceratophrys*, the muscle does not occupy any space outside the adductor chamber itself (SEE pers. obs), and the parietosquamosal shelf extends into the space above the crista parotica. Nevertheless, the total muscle volume is comparable and associated with an increased depth of the skull. *Beelzebufo* probably had a similar arrangement.

By far the most unusual feature of the *Beelzebufo* squamosal revealed by FMNH PR 2512 is the presence of the large posteriorly directed flange. The tip of this flange is rounded and unsculptured (Figs. 10, 18F,H, 19E), suggesting it was not in direct contact with the skin, but that the rest of the external surface of the flange clearly was. The ventral margin of the flange bears a large multiple-laminated quadratojugal facet that is oriented vertically at its anteromedial end but becomes more horizontal posteroventrally (Fig. 18D,I). Due to the position of the flange, and the sculpture over most of its external surface, the depressor mandibulae muscle must have run deep to it, originating on the posterodorsal edge of the squamosal (and possibly dorsal fascia).

FMNH PR 2536 (Fig. 18F–G) was originally interpreted as the tip of a squamosal otic process ([26]: fig. 3K), but is now re-identified as the posterolateral tip of a fused squamosal-quadratojugal flange. The rounded, unsculptured tip resembles the end of the squamosal flange in FMNH PR 2512 and UA 9625, but there is no ventral quadratojugal facet and the fragment has intact dorsal, ventral, and posterior margins. It therefore seems to represent an individual in which the squamosal and quadratojugal have fused without trace of the suture, although this element is slightly smaller overall than some other specimens (e.g., UA 9674) in which the sutures remained fully open. As previously suggested [26], adults of *Beelzebufo* may have reached skeletal maturity at different sizes, possibly associated with sexual dimorphism.

#### Quadrate and Quadratojugal

**Figure 20:**
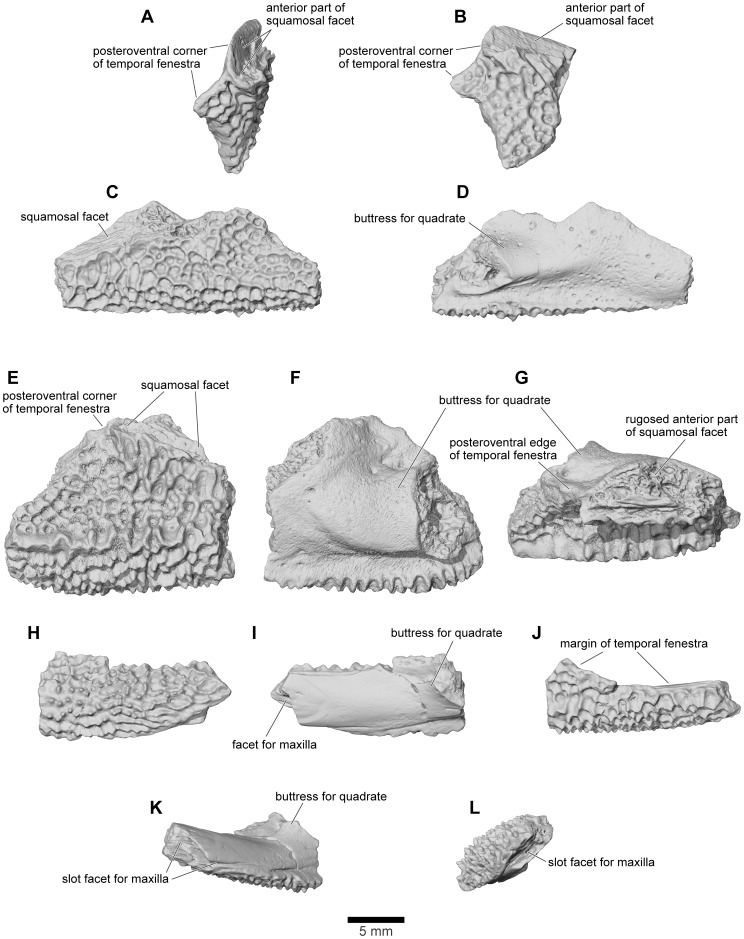
Quadratojugal. **A**, dorsolateral; and **B**, lateral views of left quadratojugal, FMNH PR 2512. **C**, lateral; and **D**, medial views of right quadratojugal, FMNH PR 1959. **E**, lateral; **F**, medial; and **G**, dorsal views of left quadratojugal, UA 9639. **H**, lateral; **I**, medial; **J**, dorsolateral; **K**, anteroventromedial; and **L**, anteromedial views of right quadratojugal, UA 9621.

FMNH PR 2512 preserves the intact posterior part of the right quadratojugal and quadrate (Figs 11, 19D–F), the left quadrate (Fig. 19A–C: this piece also retains parts of the left quadratojugal), and two separate portions of the postquadrate flange of the left quadratojugal (anterior portion with suture for squamosal, Fig. 20A–B). FMNH PR 2512 is supplemented by several isolated specimens, including FMNH PR 1959 (Fig. 20C–D), FMNH PR 2500, FMNH PR 2501, FMNH PR 2536, UA 9618, UA 9621 (Fig. 20H–L), UA 9624, UA 9639 (Fig. 20E–G), UA 9674, and UA 9956. Prior to the discovery of the articulated portions of FMNH PR 2512 and the realisation that the quadratojugal was uniquely and enormously expanded posteriorly, several of the isolated specimens were interpreted as portions of the squamosal ([25], fig. 1N, O; [26], fig. 3H, I, K) or nasal ([26], fig. 3E).

Like the squamosal, the quadratojugal of *Beelzebufo* is unique in being drawn into an extraordinarily long tapering postquadrate flange (Figs. 11, 19D–F). Unlike the squamosal flange, that of the quadratojugal is not rounded at its posterior end; instead, its terminus is pointed. In isolation, this flange looks like the anterior process of the nasal in other anurans, the straight suture for the squamosal resembling the straight internasal suture. Consequently some of the fragmentary specimens originally attributed to the nasal [26] belong instead to this tapering process. The straight dorsal edge of the process bears a strongly laminated facet for articulation with the corresponding flange of the squamosal (Fig. 20C,E,G). This facet begins anterodorsolaterally, where it is wide and partly scarfed, and then tapers along the posterodorsal margin, tightly matching and interdigitating with the facet on the squamosal. The ventral surface also narrows posteriorly, being thick and ridged near the jaw joint and thinner posteriorly. In the midsection of the bone, the medial surface is drawn into a strong buttress that supports the lateral aspect of the quadrate and a thinner anteromedial lamina (Figs. 19F, 20D,F). FMNH PR 1959 shows how this buttress narrows anteriorly and gradually levels out (Fig. 20D).

None of these specimens, except perhaps UA 9621, preserves the anterior end of the quadratojugal as it ran under the temporal fenestra to meet the maxilla. UA 9621 (Fig. 20H–L) is clearly part of a larger bone, with the process tapering either anteriorly or posteriorly. The lateral surface is covered with sculpture (unlike the pars dentalis of the maxilla, which is smooth externally). The medial surface bears a flattened but dorsoventrally deep ridge that expands medially at one end and has a slot facet at the other (Fig. 20K,L), where it articulated with a similarly shaped process from another bone. If correctly identified as an anterior process of the quadratojugal, this specimen indicates that there was a relatively narrow bar below the middle part of the temporal fenestra, thickening both anteriorly and posteriorly.

#### Pterygoid

The posterior part of the triradiate right pterygoid is associated with the quadratojugal in FMNH PR 2512. No other specimens of the pterygoid have been identified, probably because the bone is thin, easily fragmented, and also unsculptured.

The pterygoid of FMNH PR PR 2512 preserves its posterior and medial processes (Fig. 11). The latter is narrow and rather crushed so that its original height and orientation are difficult to gauge. The medial end bears a facet on its posterodorsal surface for articulation with the right alar process of the parasphenoid. As preserved, the facet is somewhat V-shaped in section but begins to flatten out distally although we cannot be certain of the length of that contact. The two bones cannot be brought into articulation in the specimen due mainly to the dorsoventral compression of the braincase, although it is possible that the tips of the pterygoid and parasphenoid are also missing. The almost complete posterior process lies at an angle of roughly 100° to the medial one. It is deep and forms a concavo- (laterally) convex (medially) blade that is slightly twisted around its long axis from posteromedial to anterolateral (Fig. 19D–F). Posteriorly, the process strongly overlapped the quadrate (Fig. 19B–C) and met the thin medial lamina of the quadratojugal along at least part of its dorsal edge. The base of the anterolateral pterygoid process lies lateral to the junction of the medial and posterior processes but the remainder is broken away.

#### Braincase

**Figure 21:**
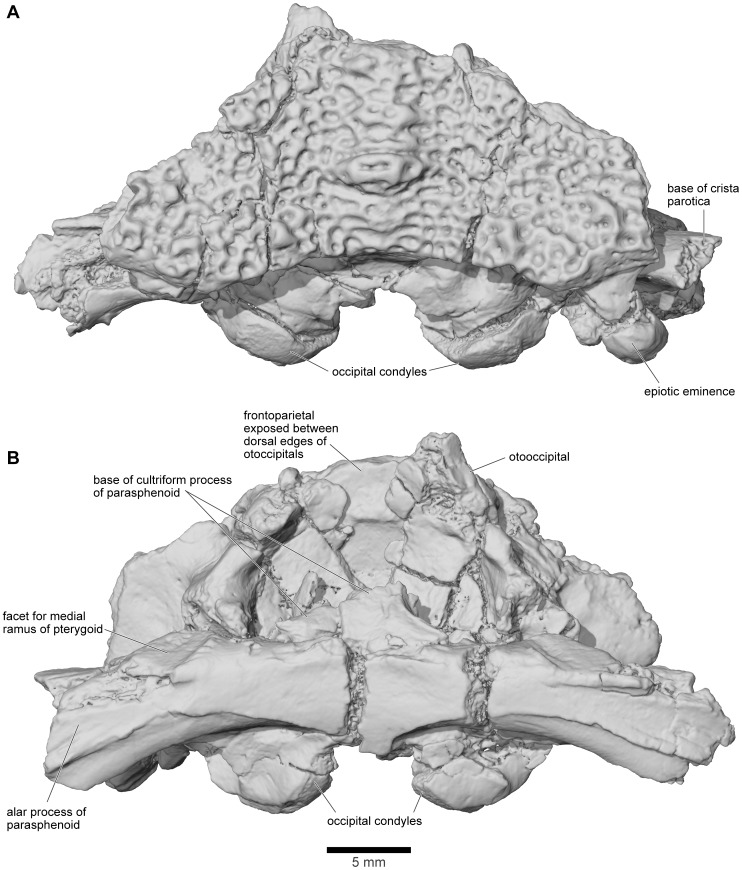
Frontoparietal and braincase, FMNH PR 2512. **A**, dorsal; and **B**, ventral views.

**Figure 22:**
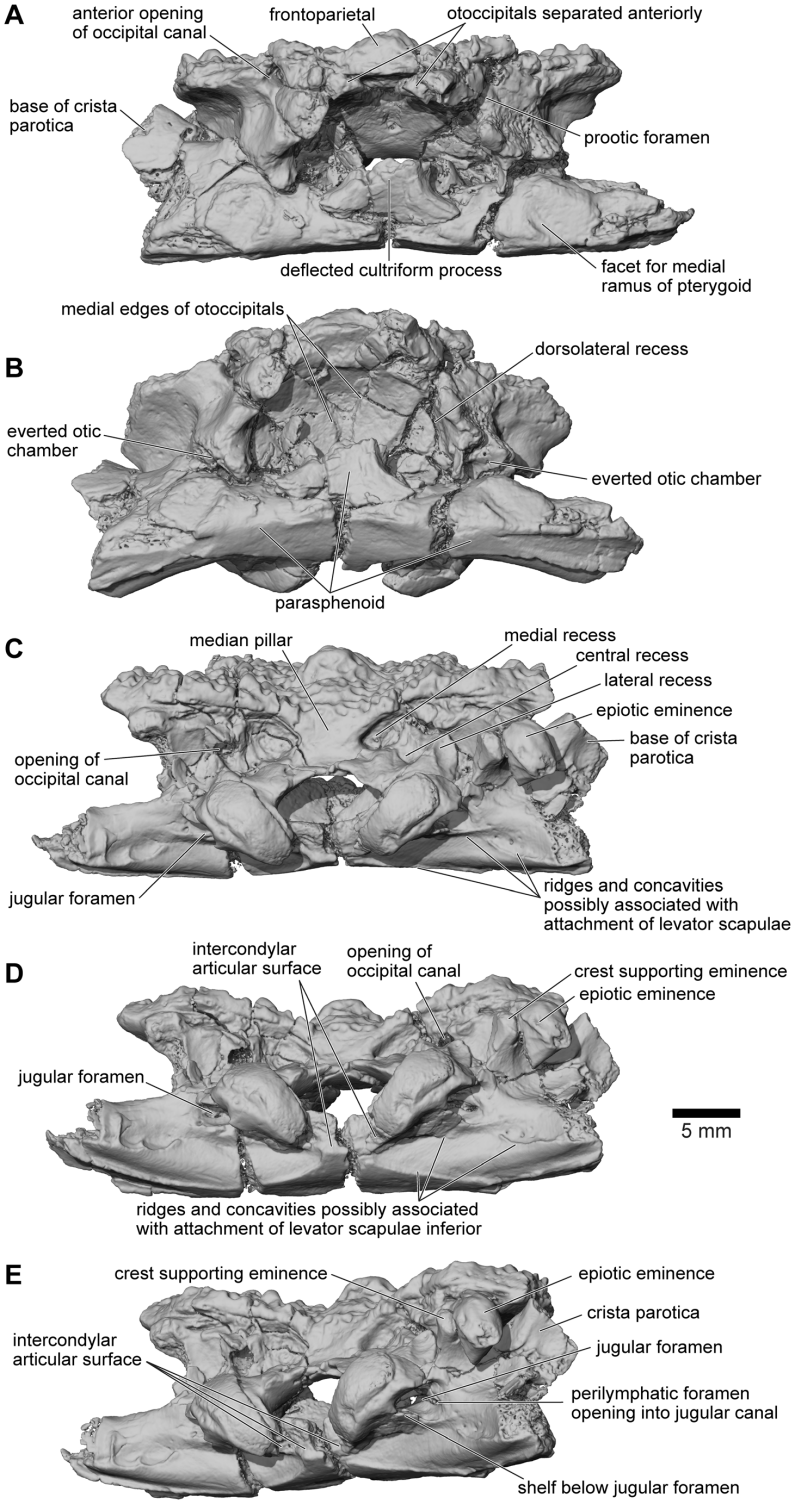
Frontoparietal and braincase, FMNH PR 2512. **A**, anterior; **B**, anteroventral; **C**, posterior; **D**, posteroventral; and **E**, oblique right posterolateral views.

**Figure 23:**
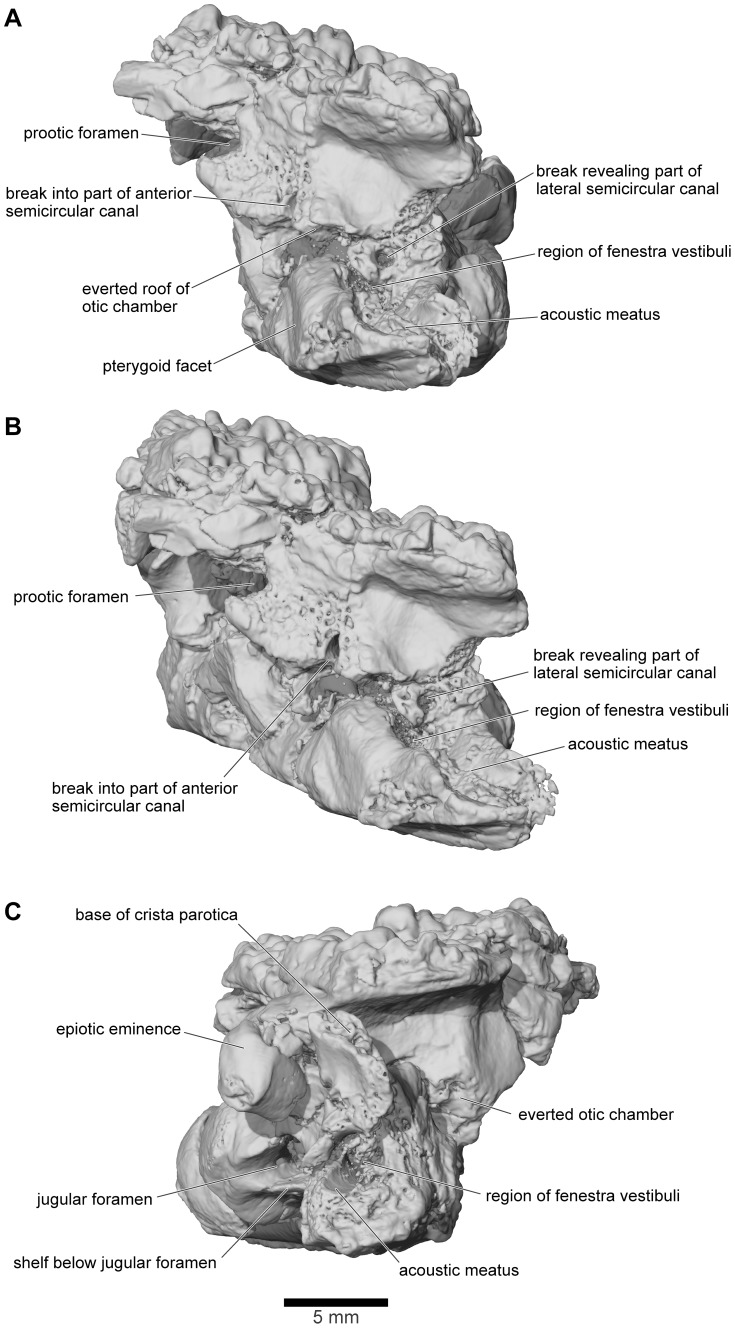
Frontoparietal and braincase, FMNH PR 2512. **A**, left lateral; **B**, left anterolateral; and **C**, right lateral views.

**Figure 24:**
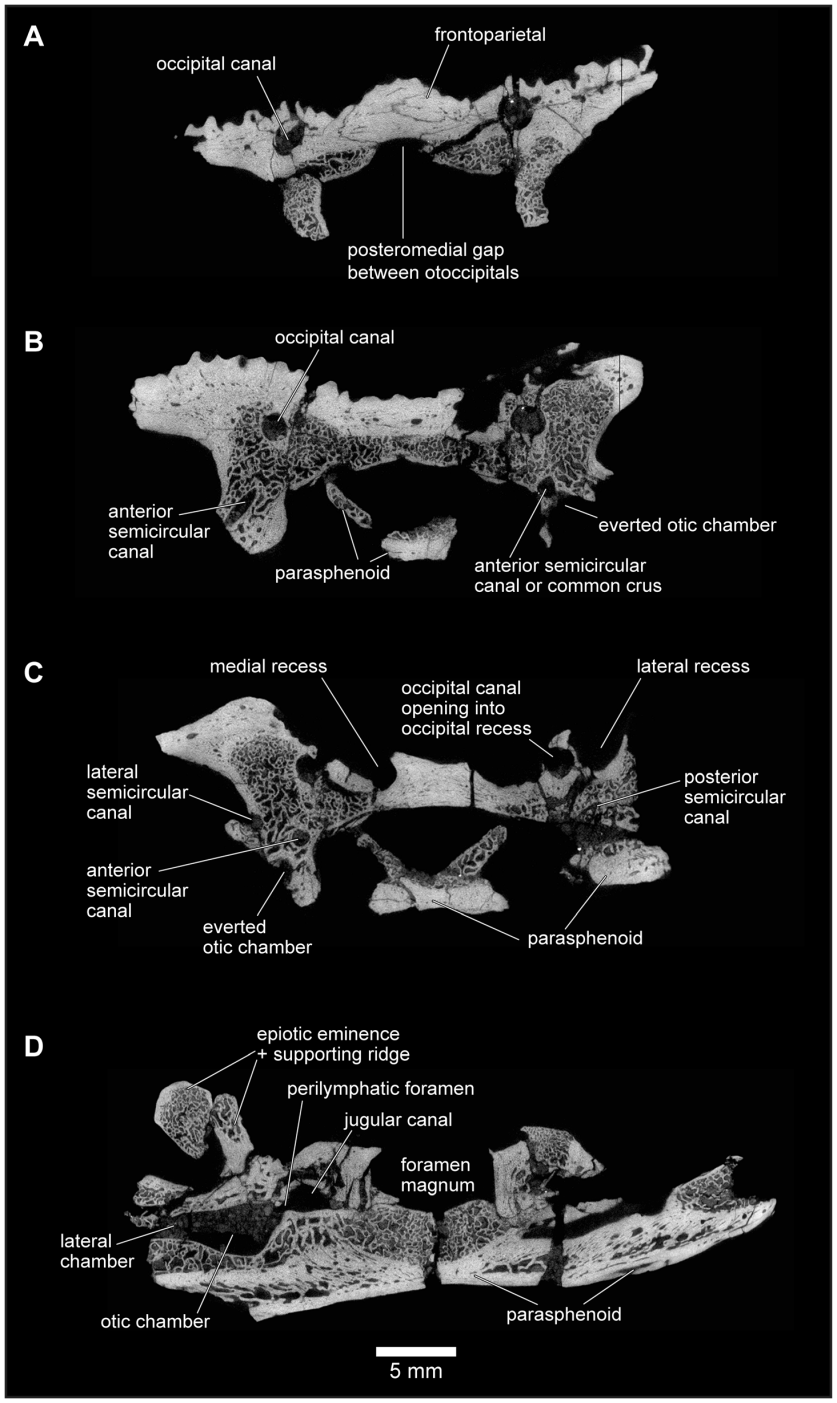
Approximately anterodorsal-posteroventral progression of μCT slices through braincase, FMNH PR 2512. **A**, slice YZ 386; **B**, slice YZ 637; **C**, slice YZ 719; and **D**, slice YZ 992. Scan slices in YZ plane of reconstructed volume. Note that anteroposterior and dorsoventral biological axes deviate approximately 45° from scan reconstruction XZ and YZ axes.

**Figure 25:**
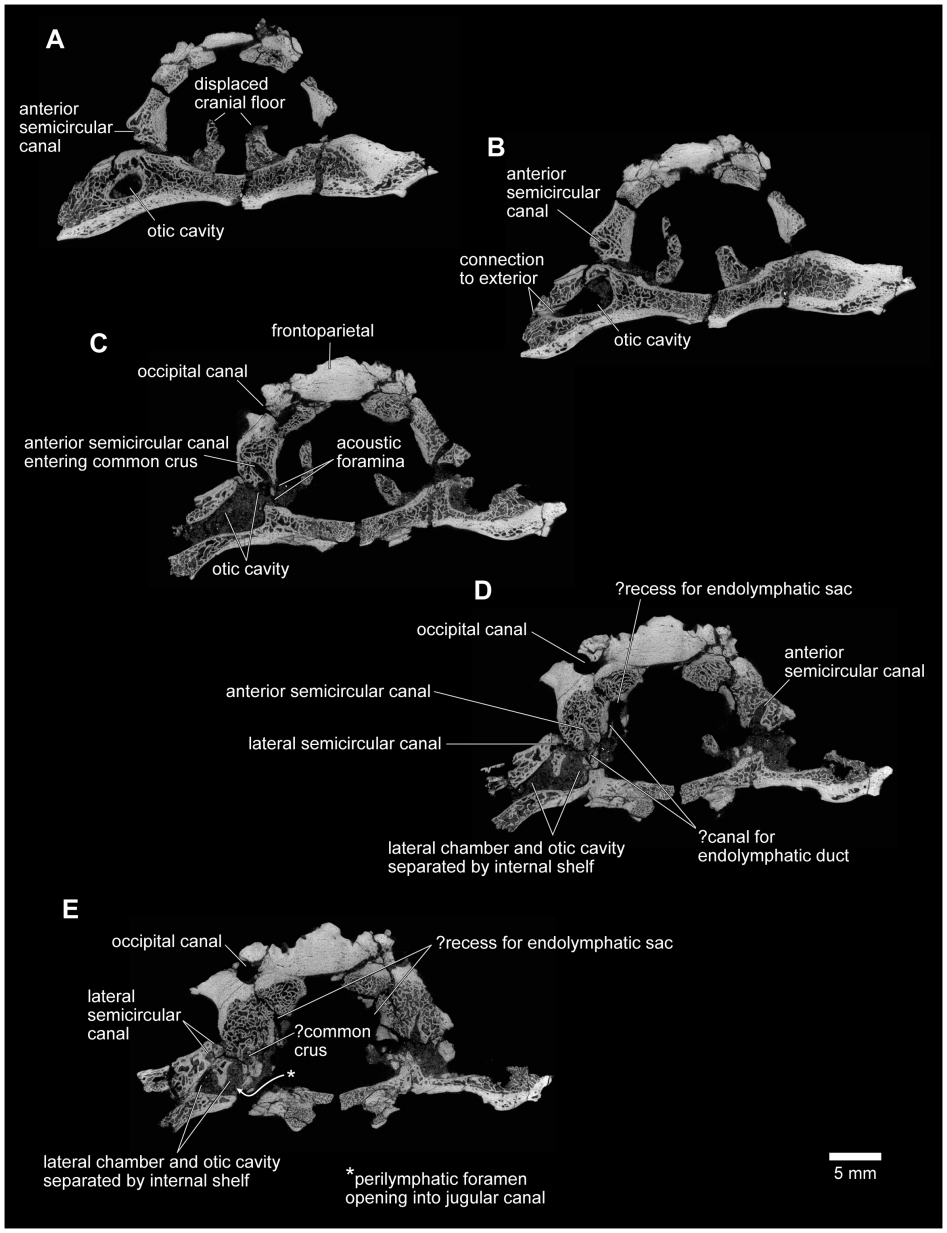
Approximately anteroventral-posterodorsal progression of μCT slices through braincase, FMNH PR 2512. **A**, slice XZ 290; **B**, slice XZ 315; **C**, slice XZ 370; and **D**, slice XZ 425. Note that anteroposterior and dorsoventral biological axes deviate approximately 45° from scan reconstruction XZ and YZ axes.

**Figure 26:**
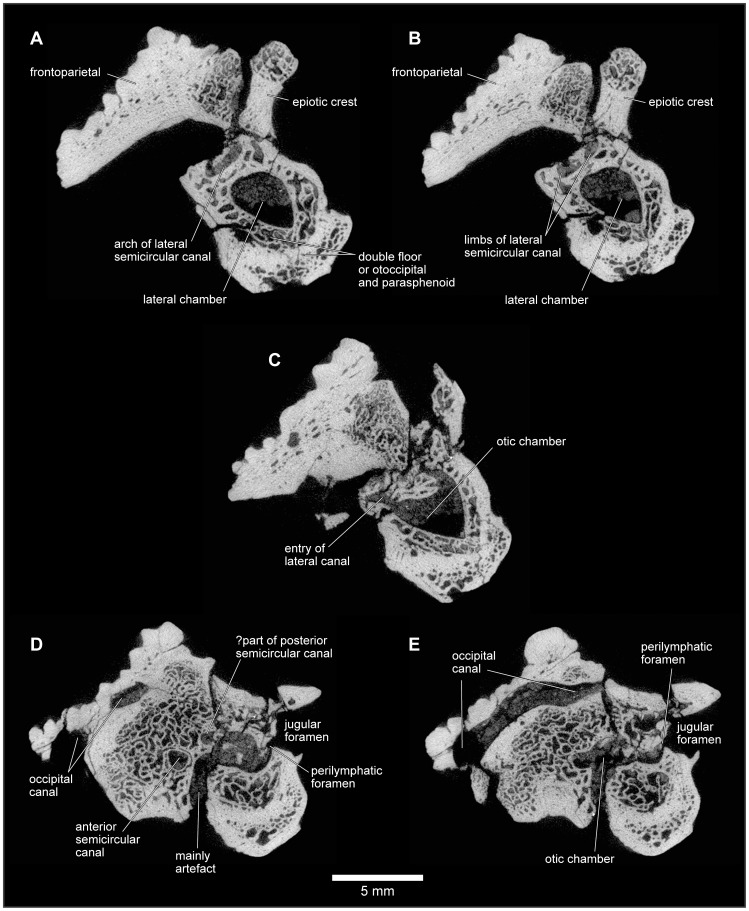
Mediolateral progression of μCT slices through right ear region, FMNH PR 2512. **A**, slice XY 425; and **B**, slice XY 441, through lateral semicircular canal and otic chamber. **C**, slice XY 500, close to boundary between lateral semicircular canal and otic chamber. **D**, slice XY 640; and **E**, slice XY 665, including occipital canal. Scan slices in XY plane of reconstructed volume.

FMNH PR 2512 preserves an almost complete posterior braincase (paired otoccipitals conjoined dorsally by the frontoparietal and ventrally by the parasphenoid, but no sphenethmoid) and is supplemented by UA 9675, the left half of a braincase. The braincase of FMNH PR 2512 was μCT scanned and the slices used to create the 3-D images in Figs. 21–23. The μCT scan slices demonstrate a striking difference between the very dense laminar bone of the dermatocranial surface and the complex, porous endochondral bone of the otoccipitals (Figs. 8, 24–26), a pattern seen also in hyperossified living anurans like *Pyxicephalus* (SEE pers. obs.) and some casque-headed hylids [67].

The braincase of FMNH PR 2512 is largely complete but it is dorsoventrally compressed, probably to around 60–70% of its original height. This compression has left the thick dorsal and posterodorsal surfaces and, to a slightly lesser degree, the ventral surface largely intact, but the thinner walled otic capsules have been crushed with their anteroventral parts rotated outward. This has exposed portions of the internal otic chamber on the lateral surface and resulted in the loss of parts of the anterior and horizontal semicircular canals and ampullae, as well as damage in the occipital region to the upper parts of the posterior semicircular canals. The damage is greater, and extends farther posteriorly, on the left side than on the right. The compression has also reduced the height of the foramen magnum and the occipital surface above this level, distorting the crista parotica and epiotic ridge/prominence and disrupting the articulation between the pterygoid and the alar process of the parasphenoid. The sphenethmoid region is not preserved on any specimen. Although not preserved in its entirety, the parasphenoid appears to have been T-shaped or slightly cruciform. Narrow alar processes extend along the full width of the otic capsules, directed slightly posteriorly but the tips are broken and the orientation is probably not natural. A short posteromedial process underlies the foramen magnum, and the base of the cultriform process, narrowing anteriorly, is preserved in the anterior midline but compression of the specimen has caused it to be deflected posterodorsally into the endocranial cavity. As seen in anterior and anteroventral views (Fig. 22A–B), symmetrical depressions on either side of the cultriform process represent the surfaces of articulation for the medial rami of the pterygoids. As preserved, the long axes of these facets run ventrolateral to dorsomedial, perhaps reflecting a more pronounced original ventrolateral angulation of the parasphenoid alar processes (rendered secondarily horizontal by compression).

On the occipital surface of FMNH PR 2512 (Fig. 22C–E), the foramen magnum lies between two elongated strap-like occipital condyles, their axes oriented dorsolateral to ventromedial. These narrow ventromedially, are not stalked, and, in life, appear to have been joined across the midline by a thin continuous articular surface that articulated with the matching surface on the median lip of the atlas (see below). Breakage in the area between the occipital condyles, including a major midline crack passing through the parasphenoid, has damaged the median articular surface, giving the impression that the condyles were separated medially. However, careful examination using both light microscopy and μCT scans shows that parts of the median surface are preserved. The bases of the condyles are perforated mediolaterally by jugular canals (Fig. 22E) that open from the cranial cavity and conveyed the glossopharyngeal and vagus nerves, as well as the internal jugular vein. Perilymphatic foramina from the otic capsule open into the jugular canals (see also below), which, in turn, open on to the occipital surface through the jugular foramina. A thin, sharp ledge extends across the ventral limit of each jugular foramen forming a frame across which a compensatory ‘round window’ would have stretched [68]. The shelf may have served to increase the size and effectiveness of the round window but also separates the window from a distinct ventral concavity that, by comparison with living anurans [68], [69], may have housed the levator scapulae inferior muscle.

**Figure 27:**
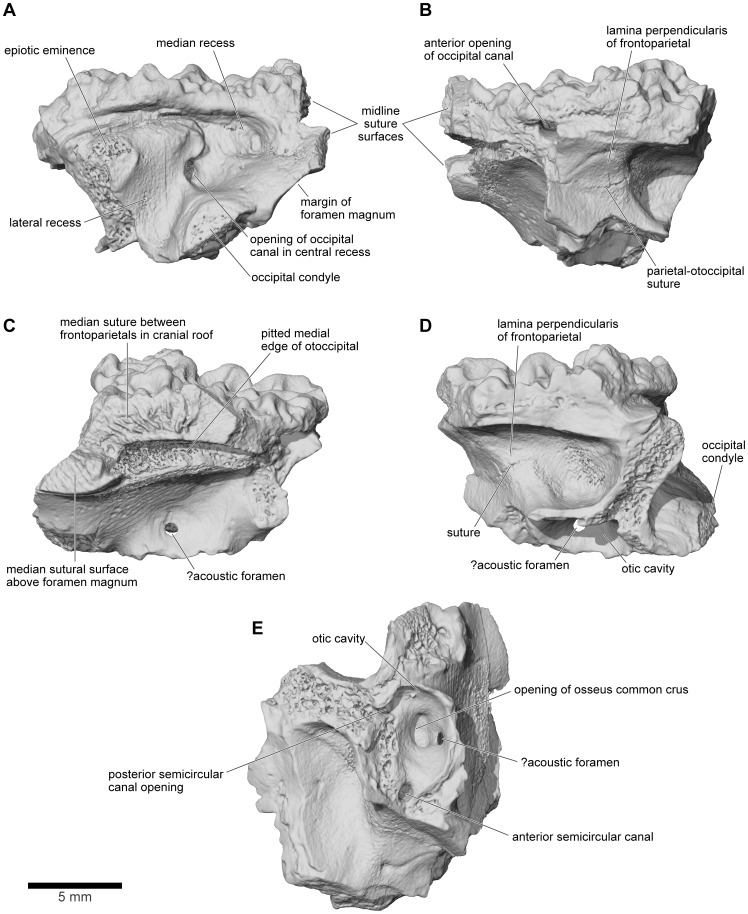
Left frontoparietal and otoccipital, UA 9675. **A**, posterior; **B**, anterior; **C**, medial; **D**, lateral; and **E**, ventrolateral views.

From FMNH PR 2512 and UA 9675, it is clear that the otoccipitals met in the posterodorsal midline to roof the braincase. In UA 9675 (Fig. 27), a partial left otoccipital is fused to the overlying frontoparietal, but each bears a separate, articular surface for the contralateral element (Fig. 27C), suggesting that the individual represented by UA 9675 had not completed development. As preserved, the frontoparietal articular surface is deep and laminated to form a strong median joint. The more posterior articular facet on the otoccipital is intact and discrete from that on the frontoparietal. It is short and ridged, showing that the left and right otoccipitals met in an interdigitated suture over the foramen magnum. Anterior to this sutural surface, the intact but pitted medial edge of the otoccipital angles laterally so that a triangular space, possibly completed in cartilage, was formed in the dorsal midline between the left and right otoccipitals. In FMNH PR 2512, the left and right frontoparietals and otoccipitals are fused with no trace of the original sutures. Posteriorly, they contribute to the formation of a thick, posterior median pillar that supports the frontoparietal (Fig. 22C–E). The pillar divides the dorsal occipital surface into distinct bilateral recesses, each of which is further subdivided into medial, central, and lateral parts that are aligned in dorsomedial to ventrolateral sequence. The medial recesses are the largest in diameter and deepest, and are separated from the central recesses by weak crests. The central recesses are flanked laterally by stronger crests; the occipital canals that carried the occipital arteries forward toward the orbit open from the dorsolateral corners of these crests. The most lateral recesses are flanked in turn by strong crests that run to the epiotic eminences (sensu [59]; see also [70]), tuberosities [clearest on the right in FMNH PR 2512, Fig. 22C–E]) that develop over the rounded ridge marking the course of the posterior semicircular canal and are associated with the attachment of part of the intertransversalis capitis muscle [71], [72]. On the right, the epiotic eminence has been displaced ventrolateral to the crest that leads up to it whereas, on the left, its terminus has been broken away. Laterally, each otoccipital is extended dorsally into a thick crista parotica that met the lateral edge of the frontoparietal (see above) and ventrally into a vertical flange that articulated with the alar process of the parasphenoid to form a posterior wall to an acoustic meatus leading to the fenestra vestibuli (Fig. 23).

The marked ridges and depressions on the occipital surface presumably represent attachment areas for strong epaxial craniovertebral muscles. In living anurans, several distinct muscle groups attach to the posterior surface of the skull, or the associated fascia. These have been named differently by various authors. Superficially the rhomboideus anterior runs from the posterior margin of the frontoparietal and adjacent fascia and attaches to the suprascapula. Deep to it, from medial to lateral, attach the deep interspinous fibres of the longissimus dorsi (intercrurales [71]; rectus capitis medialis [73]), then the superficial fibres of longissimus dorsi, and the cranial fibres of the intertransversarius (m. intertransversarius capitis superior [72]; obliquus [73]). The latter two are usually associated with the epiotic prominence, generally with at least a partially tendinous attachment ([10], [71]–[72]; SEE pers. obs. from dissections of *Ceratophrys, Osteopilus*, *Pyxicephalus,* and *Xenopus*), and the canal for the occipital artery typically opens between the medial and lateral attachments of the longissimus dorsi [71]. Based on this arrangement in living anurans, it seems likely that the deep median depressions on either side of the central midline pillar in *Beelzebufo*, and perhaps also the smaller central depressions, housed the deep interspinous portions of the longissimus dorsi, whereas the lateral depressions, their flanking crests, and the epiotic prominences may have been associated with the superficial part of the longissimus dorsi and the intertransversarius muscles. Superficial to all of these axial muscles, the anterior rhomboids would have attached to the edge of the frontoparietal and perhaps also to a narrow shelf below this, but above the occipital recesses.

In anterior view, the two otoccipitals are separated in the dorsal midline, below the roofing frontoparietal, by a substantial gap (Fig. 22A). Seen in anteroventral view (Fig. 22B), this gap is triangular and corresponds to the recess described above in UA 9675, which was possibly completed in cartilage. Ventrally, the inwardly deflected cultriform process of the parasphenoid obscures the view of the cavity. The anterolateral margins appear to be embayed, presumably by the prootic foramen (CN5+CN7), and the occipital canals open into the posterodorsal corners of the orbits between the prootics and the frontoparietal. Seen in lateral view (Fig. 23), the left side of the otic capsule bears a long acoustic meatus leading toward the inner ear, flanked posteriorly by a flange from the otoccipital and ventrally by the parasphenoid. In the uncrushed UA 9675, this surface reveals a suture line between the shallow lamina perpendicularis of the frontoparietal and the otic capsule (Fig. 27D).

The μCT scans of the braincase of FMNH PR 2512 permit a more detailed description of the ear region, which is broadly similar to that of both *Ceratophrys* and *Pyxicephalus* (SEE pers obs.). In the otic capsule of living anurans [68], a central otic chamber contains both endolymph- and perilymph-filled cavities. The endolymph-filled chambers are divided into upper and lower parts, surrounded by a perilymphatic space. The pars superior includes the utricle from which the anterior, posterior, and lateral semicircular canals extend, each terminating in an ampulla that contains a sense organ. The ampullae of the anterior and lateral canals lie anterior to the otic chamber and the ampulla of the posterior canal lies behind it. The pars inferior includes the sacculus and lagena, their sensory papillae, and their maculae. Arising close to the junction between the two parts, a small endolymphatic duct passes through a small canal/foramen in the medial wall of the otic capsule and then expands into an endolymphatic sac within the cranial cavity. The surrounding perilymphatic space is exposed to the middle ear laterally at the fenestra vestibuli, where it meets the pars interna of the columella and operculum, where present, although the perilymphatic space may or may not be extended outward into a lateral chamber [68]. The latter forms an antechamber to the main otic cavity with a special function in sound control [68]. Medially, the perilymphatic space communicates with the posterior part of the cranial cavity, emerging through one or more perilymphatic foramina into the jugular canal. The perilymphatic sac stretches across the posterior opening of the jugular foramen between the otoccipital, parasphenoid, and occipital condyle to provide a pressure release window (‘round window’). The internal jugular vein and the vagus and glossopharyngeal nerves usually also exit through the jugular foramen although they may have a separate foramen (e.g., *Pyxicephalus*, SEE pers. obs.).

**Figure 28:**
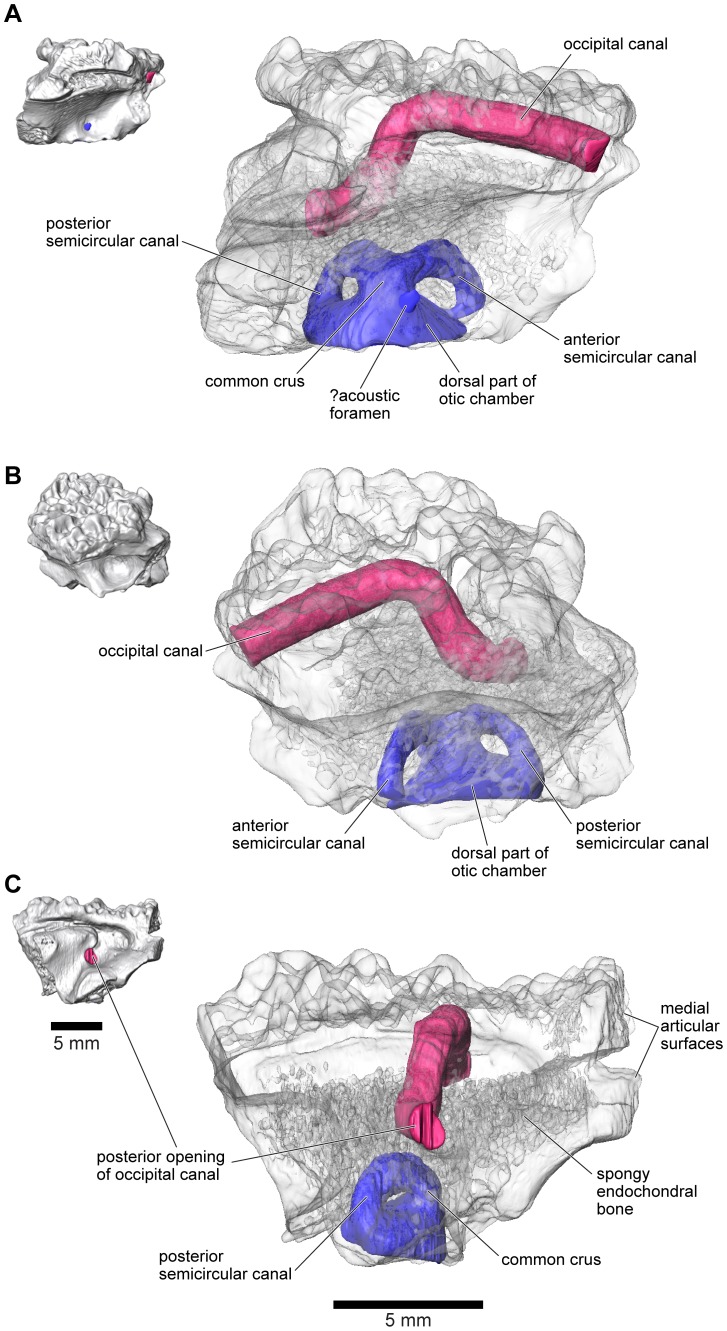
Internal morphology of left frontoparietal and otoccipital, UA 9675. **A**, medial; **B**, dorsolateral; and **C**, posterior views of digital segmentation of μCT dataset. Small opaque images at left for orientation; larger semi-transparent images at right, including occipital canal rendered in red and inner ear structures in blue.

As outlined above, the anterior and anterolateral portions of both otic capsules are damaged in FMNH PR 2512, with the loss of the anterior and lateral ampullae and those parts of the semicircular canals immediately adjacent to them. Nonetheless, the paths of the canals can be partly followed through the slices (Figs 24–26), and parts of the anterior and posterior canals running into the common crus are also preserved in UA 9675 (reconstructed in Fig. 28). In FMNH PR 2512, the lateral canal runs above the boundary of the otic cavity and the lateral chamber, close to the level of the fenestra vestibuli (Figs 24C, 25D–E, 26A–B). The lateral chamber itself is large but breakage around its margins makes it impossible to reconstruct the attachment points of either the columella (found with, but disarticulated from, the specimen, see below) or operculum. Medially, one or more acoustic foramina pierce the capsule wall carrying branches of the vestibulocochlear nerve (Fig. 25C). Most living anurans have two foramina here but the crushing makes it difficult to be certain that the opening is subdivided. A small canal runs dorsally from the upper part of the recess for the acoustic foramina, and opens into a distinct recess in the dorsolateral wall of the cranial cavity (Fig. 25D). The canal is probably for the endolymphatic duct with the endolymphatic sacs perhaps occupying the dorsolateral recesses. Posteroventrally, the otic chamber opens into the large jugular canal through the perilymphatic foramen (Figs 24D, 25E, 26E).

#### Columella

**Figure 29:**
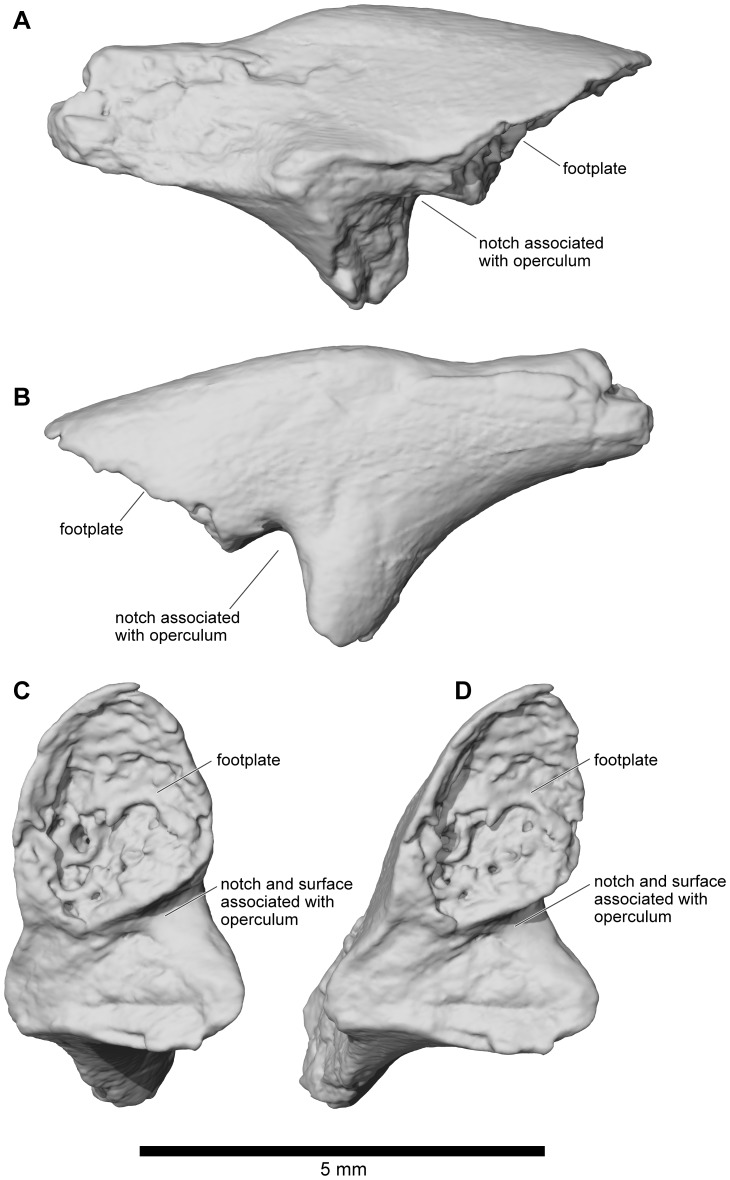
Left columella, FMNH PR 2512. **A**, posterior; **B**, anterior; **C**, medial; and **D**, oblique medial views.

The sediments around the associated cranium of FMNH 2512 were screened and all remaining bone fragments collected. Among these was a left columella that is attributed to the same individual as the rest of the skull (Fig. 29). It matches that of similarly sized large individuals of extant anurans like *Ceratophrys* (LACM 163430). It has a divided proximal (medial) end suggesting the presence of a locking mechanism [68] between it and the operculum. Its distal end is not complete but, as noted above, the structure of the squamosal suggests that there was no tympanic membrane and that the columella may have ended in the soft tissues of the head (as, for example, in the living *Bombina* [68]).

#### Angulosplenial

**Figure 30:**
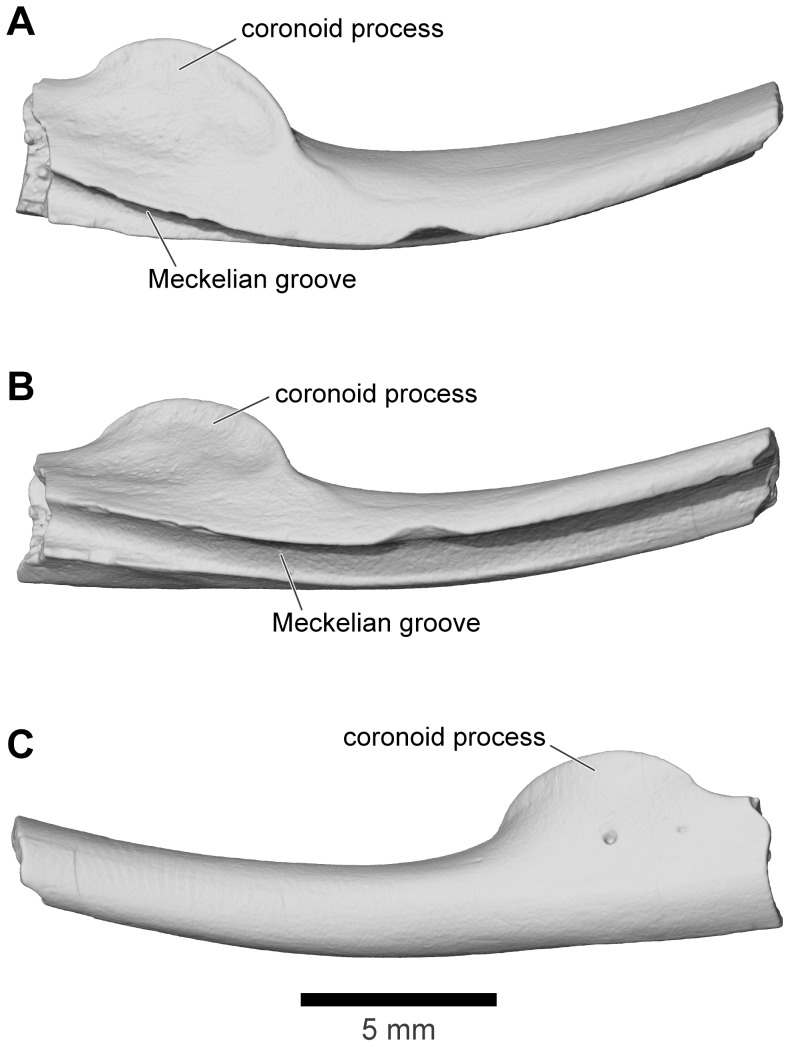
Right angulosplenial, UA 8677. **A**, dorsal; **B**, dorsolateral; and **C**, ventral views.

A partial right angulosplenial (UA 8677), described in some detail and figured by Asher and Krause ([25]: fig 1J, K) but not assigned to a taxon, is the only representative of the lower jaw in *Beelzebufo*. It shows no remarkable features (Fig. 30), apart from a rather short, rounded coronoid process. It is assigned to *B. ampinga* on the basis of its typically anuran morphology and relatively large size.

#### Dentition

**Figure 31:**
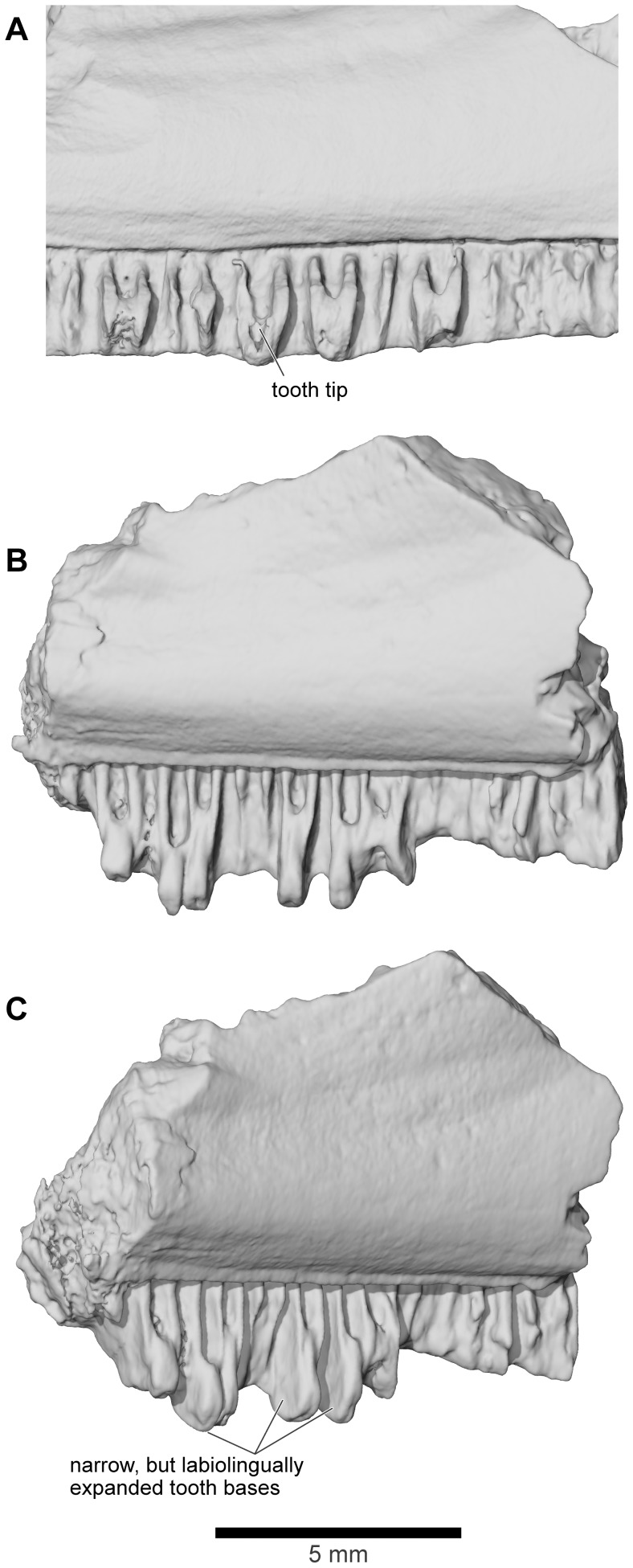
Details of maxillary dentition. **A**, lingual view of partial right maxilla showing tooth tip, FMNH PR 2506. **B**, lingual; and **C**, anterolingual views of partial right maxilla, UA 9945.

There were 50–60 teeth on each maxilla and 13–14 on each premaxilla.The teeth are not completely preserved on any specimen, but their structure can best be reconstructed from FMNH PR 2506 (Fig. 31A) and UA 9945 (Fig. 31B). The former is the midsection of a maxilla in which several teeth are preserved. These are mesiodistally narrow but labiolingually broad so that they form robust plates supported on either side by strong ridges of attachment bone (Fig. 31C). The tooth tips are broken off but what remains is a solid surface, not the cylindrical bases found in amphibians in which the pedicels have been detached. FMNH PR 2506 is a fragment of maxilla in which an unerupted tooth tip is present in a broken tooth base (Fig. 31A). It is unicuspid and tapering. Taken together, the teeth of *Beelzebufo* are strikingly similar to those of the living *Ceratophrys*, and suggest at least some degree of functional correspondence.

### Postcranial skeleton

Fewer specimens of the postcranial skeleton of *Beelzebufo* have been recovered than of the skull, presumably because roofing elements of the latter are both highly robust and also easily identified from their characteristic ornamentation. Almost nothing is known of the appendicular skeleton, except for two elements: a tibiofibula (UA 9628) and a tibiale-fibulare (UA 9957). There are also several partial anuran humeri and tibiofibulae from the Maevarano Formation that could belong to juvenile individuals of *B. ampinga*, but given their relatively small size and the suspicion that at least one small species of anuran may be present in the Maevarano Formation (based on some small but well ossified elements), their attribution is uncertain and they are therefore omitted here. The absence of adult humeri (especially the distal condylar portions) and ilia of *Beelzebufo* is puzzling, given their expected robusticity and the fact that these are usually among the most common elements in anuran-bearing fossil sites elsewhere in the world. Nonetheless, repeated and careful searches both in the field and through the collections have failed to reveal convincing representatives of either element.

**Figure 32:**
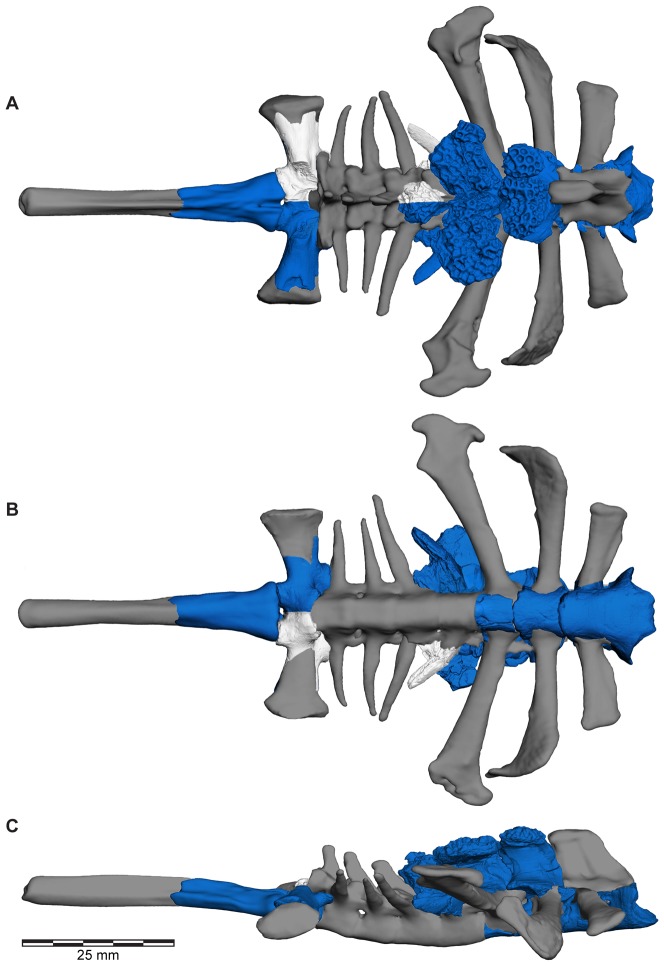
Three-dimensional digital reconstruction of axial skeleton of *Beelzebufo ampinga*. **A**, dorsal; **B**, ventral; and **C**, right lateral views of axial column. As in Fig. 1, with material of *Beelzebufo ampinga* in dark blue. Mirrored left portion of neural arch of fifth presacral vertebra in model (FMNH PR 2512 Vertebra B) and centrum and transverse process of sacral vertebra (FMNH PR 2003) are mirrored in light grey. Dark grey postcranial elements modelled on large female specimen of *Ceratophrys aurita* (LACM 163430). See Supporting Information S1 for detailed description of model.

Several additional specimens of the axial skeleton have also been discovered since the original description of *Beelzebufo* [26]; they make an important contribution to our knowledge of the postcranial anatomy of this armoured anuran (Fig. 32). All vertebrae are procoelous with hemicylindrical centra. The central articulations are slightly oblique, with the anterior cotyle facing somewhat ventrally and the posterior condyle angled somewhat dorsally. It appears that at least the third through fifth presacral vertebrae had tall thick neural spines that are triangular in cross-section and bear bilaterally expanded spine tables, the dorsal surfaces of which are coarsely sculptured like the dermal skull bones. These spine tables probably represent osteoderms or dermal shield elements that have become attached to the neural spines (as in *Ceratophrys* and *Lepidobatrachus* [59], [74]) or are expansions of the neural spines that contacted and became fused to the overlying skin.

The reconstructions in Figs 1, 2, 5, and 32 are based on available axial and hind limb specimens of *Beelzebufo* with skeletal elements of *Ceratophrys* (LACM 163430) used as a template for positioning and orientation. See Methods and Section A of File S1 for a more detailed description of how the model was created.

#### Atlas and second presacral vertebra

**Figure 33:**
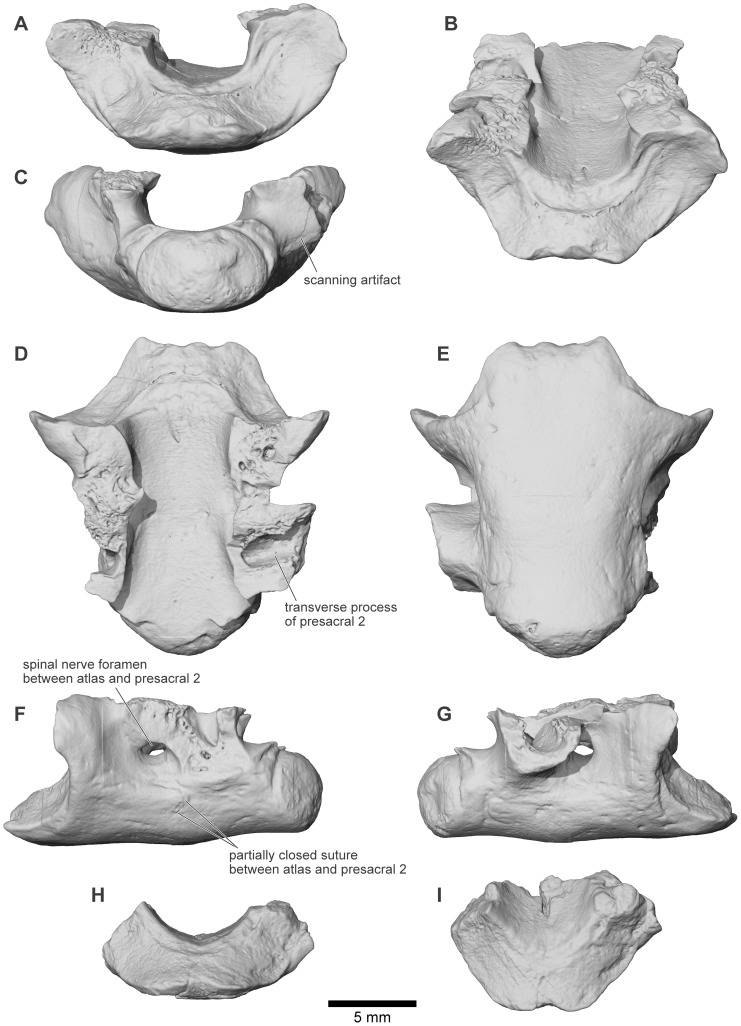
Atlas and second presacral vertebrae. **A**, anterior; **B**, anterodorsal; **C**, posterior; **D**, dorsal; **E**, ventral; **F**, left lateral; and **G**, right lateral views, UA 9600 (holotype). **H**, anterior; and **I**, anterodorsal views of atlas vertebra, FMNH PR 2512. Note scanning artifacts on UA 9600 most easily traceable as horizontal lines in D and E, and as vertical lines in F and G.

The type specimen of *Beelzebufo ampinga* (UA 9600, Fig. 33A–G) is an atlas fused to the second presacral vertebra (PS2) but with a faintly visible suture line and an enclosed intervertebral foramen between the pedicles on each side for the passage of the spinal nerve of that level. The pedicles and the bases of the transverse processes of presacral 2 are preserved but the laminae and neural spines are not. Another fused atlas + presacral 2, though less complete (recovered as two central pieces and entirely missing the neural arches), is also preserved (Fig. 33H–I); it is part of the same individual represented by FMNH PR 2512 at locality MAD98-25, thereby confirming the association of the other cranial and postcranial elements of that individual with the name-bearing type specimen (UA 9600). The atlantal cotyles match the condyles of the associated braincase of FMNH PR 2512, thus further supporting the attribution.

The atlas of UA 9600 is large, 17.2 mm across the cotyles, and 8.9 mm in midline length (measured on the ventral surface). The width across the cotyles cannot be reliably measured on the atlas of FMNH PR 2512 because of breakage on the lateral margins of both cotyles, but the equivalent width across the occipital condyles on the skull is 17.1 mm. The suture line of fusion between the atlas centrum and presacral 2 cannot be discerned and therefore midline length cannot be measured on FMNH PR 2512 either. Nonetheless, the fused element (atlas + presacral 2) appears to be as long as, or even slightly longer, than that of UA 9600 but it is also less robust; whether this is owing to dimorphism, or ontogenetic or individual variation, cannot be determined.

In anterior view (Fig. 33A,H), the cotyles on the atlantes of both specimens are narrow and strap-like, with a long axis running from dorsolateral, where the concavity is greatest, to ventromedial. These cotylar surfaces meet at the midline so that the atlas matches that of Lynch's Type III, as found in Ceratophryidae and ascaphids [60], in which the cotylar surfaces are confluent. Where the cotyles come together medially, they form a square-tipped protruding lip that abuts the thin articular surface between the occipital condyles on the skull (Fig. 33B). The atlas of FMNH PR 2512 has the same morphology as the holotype except that the median lip has a more obviously bilobed anterior margin (Fig. 33I).

The centrum of the second presacral of UA 9600 is 7.2 mm long (measured along the ventral midline and not including the posterior condyle); it is therefore considerably shorter than that of the atlas. It is also robust, with a broad, posterior condyle that is dorsoventrally compressed (transverse width = 7.4 mm; height = 4.6 mm; proportion = 1.6). The hollow cylindrical transverse processes are represented only by their bases (Fig. 33D–E). The centrum of the second presacral of FMNH PR 2512, the length of which cannot be measured, has a posterior condyle that is 6.3 mm wide and 4.2 mm high (proportion = 1.5).

#### Other presacral vertebrae

**Figure 34:**
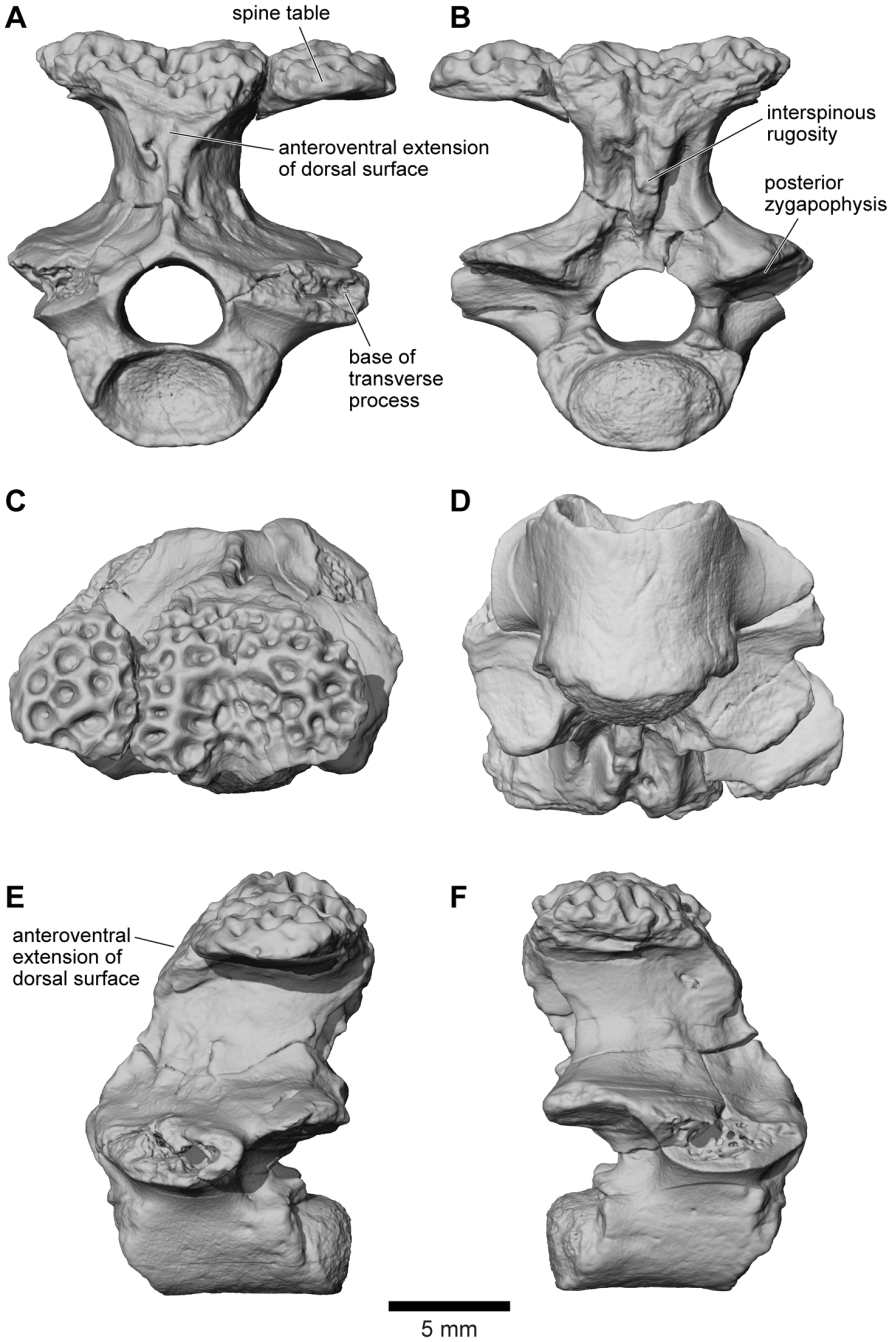
Presacral vertebra, UA 9947. **A**, anterior; **B**, posterior; **C**, dorsal; **D**, ventral; **E**, left lateral; and **F**, right lateral views. UA 9947 interpreted as possible third presacral vertebra and placed in that position for digital reconstruction in Figs 1, 2, 5, and 32.

**Figure 35:**
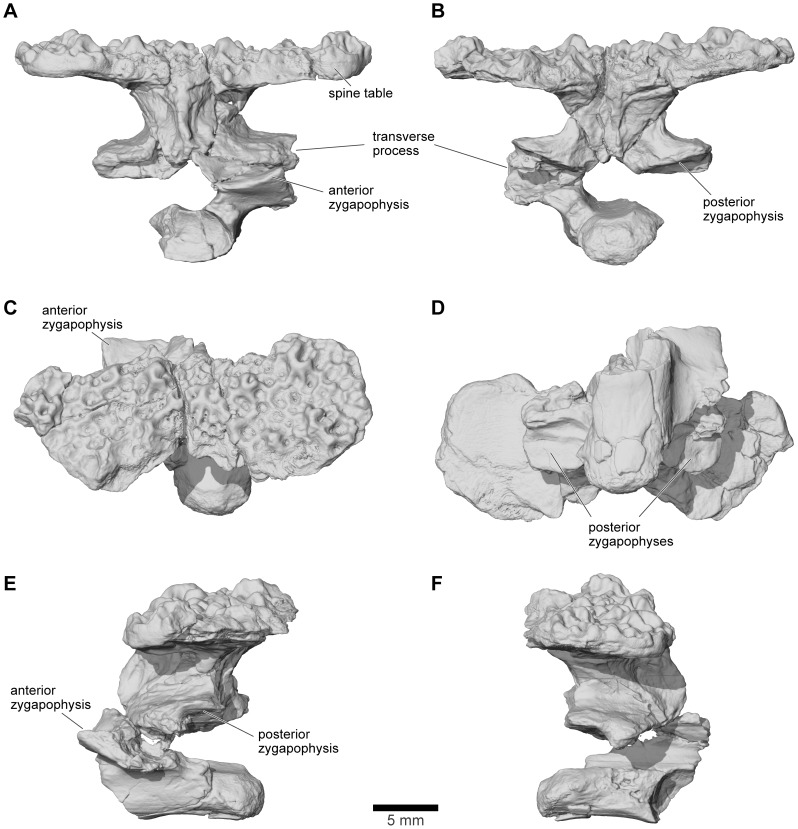
Presacral Vertebra A, FMNH PR 2512. **A**, anterior; **B**, posterior; **C**, dorsal; **D**, ventral; **E**, left lateral; and **F**, right lateral views. Vertebra A interpreted as possible fourth presacral vertebra and placed in that position for digital reconstruction in Figs 1, 2, 5, and 32.

**Figure 36:**
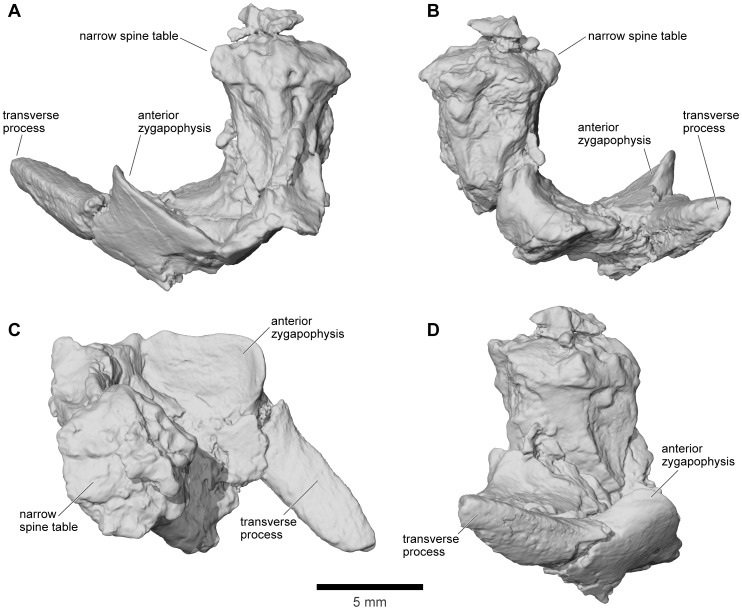
Presacral Vertebra B, FMNH PR 2512. **A**, anterior; **B**, posterior; **C**, dorsal; and **D**, right lateral views. Vertebra B interpreted as possible fifth or sixth presacral vertebra and placed in fifth presacral position for digital reconstruction in Figs 1, 2, 5, and 32.

**Figure 37:**
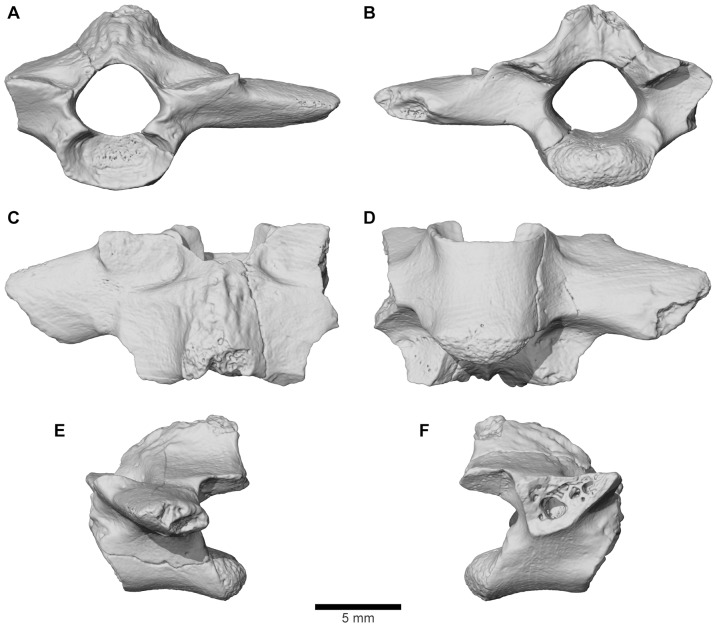
Presacral vertebra, UA 9948. **A**, anterior; **B**, posterior; **C**, dorsal; **D**, ventral; **E**, left lateral; and **F**, right lateral views. UA 9948 interpreted as sixth or seventh presacral vertebra.

Three other presacral vertebral specimens (in addition to the fused atlas + presacral 2) and several other spine table fragments were found associated with the cranial material of FMNH PR 2512. These are supplemented by four isolated specimens from other localities: UA 9947, a well preserved and nearly complete vertebra; UA 9948, a vertebra missing part (left) or all (right) of the transverse processes and all but the base of the neural spine; FMNH PR 2504, a vertebral centrum and partial neural arch; and UA 9954, a vertebral centrum with the bases of the pedicels. Together, these elements can be arranged into an approximated presacral series, based on a combination of centrum length, transverse process morphology, zygapophyseal size, and neural spine morphology (Figs 32, 34–37). In addition, a number of sculptured fragments are probably (UA 9619, UA 9627, UA 9678) or possibly (FMNH PR 2497, UA 9632, UA 9637, UA 9952) parts of vertebral spine tables.

UA 9947 (Fig. 34) is a comparatively well preserved presacral vertebra (PS), although the transverse processes are broken. The centrum is relatively long (L = 7.1 mm, measured along the ventral midline and not including the condyle) and the neural arch bears a tall robust neural spine capped by a bilaterally expanded, sculptured spine table, which, though now broken, was ∼19.5 mm in transverse width during life. The base of the neural spine is triangular in horizontal section, with the lateral surfaces angling from posterolateral to anteromedial and meeting anteriorly in a sharp median crest. The posterior surface of the spine is broad and almost flat except for a low midline ridge and paired recesses on the medial edges of the posterior zygapophyses (Fig. 34B). This suggests the presence of strong interspinal muscles and/or ligaments. The shafts of the transverse processes are not preserved but the broken cross-sections of their bases are dorsoventrally compressed and hollow. The sculptured dorsal surface of the spine table extends anteroventrally into a V-shape on the cranial face of the neural spine (Fig. 34A). Due to this anteroventral extension, we interpret this vertebra as being the first of the presacral series with an expanded spine table and therefore probably the third presacral. This would be consistent with the presence of flatter, more expanded spine tables on more posterior vertebrae.

The three presacral vertebral specimens recovered with the cranial and postcranial elements at MAD98-25 all bear the same catalogue number (FMNH PR 2512). To facilitate description and identification in the text and figures, they are here informally designated FMNH PR 2512 Vertebra A, FMNH PR 2512 Vertebra B, and FMNH PR 2512 Vertebra C. The first (FMNH PR 2512 Vertebra A) is a nearly complete vertebra (Fig. 35) that is interpreted as being from near the middle of the presacral series, and perhaps represents the fourth presacral. It resembles UA 9947 in having a tall, robust neural spine capped with an sculptured spine table, but differs in that the sculpture does not extend anteroventrally, the spine table is more than 40% wider (transverse width = 28.0 mm), and the centrum is longer (L = 7.7 mm). In extant frogs, vertebrae in the mid-column usually possess the longest centra (Table S3 in File S1). As on UA 9947, the anterior and posterior zygapophyses are short and wide. If two casts of FMNH PR 2512 Vertebra A are artificially articulated with one another, the spine tables contact suggesting there was some imbrication or, at least, fibrous connection between these parts of the dorsal armour. As on UA 9947, the transverse processes are broken but what remains of the left process is dorsoventrally compressed distally.

A second presacral vertebra (FMNH PR 2512 Vertebra B) is relatively poorly preserved and much less complete. The centrum is broken away, but the portion of the neural arch that is preserved shows salient features (Fig. 36). The spine is relatively tall. At first glance, it appears that the spine table has been broken, leaving only the central part but, in fact, the edges appear almost intact and therefore the neural spine bears only a narrow dorsal rugosity (Fig. 36C). The anterior and posterior zygapophyses are short and wide. The vertebra retains part of a short, rod-like, and tapering right transverse process with a posterolateral orientation (Fig. 36C). FMNH PR 2512 Vertebra B is interpreted as pertaining to the middle portion of the presacral series, perhaps PS5, which, at least in LACM 163430 (*Ceratophrys aurita*), is the vertebra with the most posteriorly canted transverse process. Also found at locality MAD98-25, but much more weathered, is a third vertebral specimen (FMNH PR 2512 Vertebra C, not figured) that preserves complementary parts to FMNH PR 2512 Vertebra B. It is comprised of a long centrum (L = 7.7 mm) and partial right neural arch, but no contact point could be found between FMNH PR 2512 Vertebra B and FMNH PR 2512 Vertebra C. Whether or not these two specimens are part of the same individual vertebra, FMNH PR 2512 Vertebra C probably also came from somewhere in the mid-presacral series.

UA 9948 (Fig. 37) is almost complete except for the transverse processes, a section of the anterior cotyle, and the posterodistal part of the neural spine. The centrum (L = 6.0 mm) and neural arch are relatively short, and the neural spine is low and positioned relatively posteriorly on the arch. There is no spine table. Without the neural arch on the fused atlas + PS2 vertebra, it is difficult to be certain of the overall spinal profile, but comparison with extant taxa like *Ceratophrys* suggests that the anterior neural spines are more likely to have been relatively tall. Moreover, in other robust frogs (e.g., *Ceratophrys*, *Lepidobatrachus*, *Brachycephalus*), the armour is most fully developed over the anterior half of the body. The base of the transverse process in UA 9948 is hollow, but part of the left process is preserved and shows that it was angled somewhat posteriorly in life. The short centrum, the low, posteriorly positioned neural spine without a spine table, and the posteriorly angled transverse processes indicate that this vertebra is probably from the posterior end of the presacral series. However, although the anterior zygapophyses are short and wide, the postzygapophyses are narrow and do not correspond in shape to the broad anterior zygapophyses of the sacral vertebra. We therefore interpret this vertebra as possibly PS7, or even PS6, rather than PS8, but because of the uncertainty it is not included in the digital reconstruction (Figs 1, 2, 5, 32).

These vertebral specimens are supplemented by two others that are less complete: FMNH PR 2504, a presacral with a length (L = 7.2 mm without condyle) similar to those of FMNH PR 2512 and deep transverse processes (and therefore probably from the mid-presacral region, ∼PS4/5); and UA 9954, a relatively short, broad centrum (L = 5.6 mm) that could have derived from the posterior part of the presacral series (but also could belong to a relatively small individual).

Taken together, these various vertebral specimens reveal several key points about the presacral series (Fig. 32):

1. The neural spines were tall (at least in the anterior and middle parts of the presacral series), thick, and posteriorly wide (triangular cross-section), with a large gap between the underside of the spine table and the dorsal surface of the neural arch. This gap presumably held strong epaxial muscles, tendons, and ligaments. The broad posterior surfaces of the neural spines and sharp anterior crests are suggestive of strong interspinal muscles, flanked by intertransversarius muscles. Correspondingly, the deep recesses in the occipital region of the skull are also indicative of powerful craniovertebral muscles.
2. The spine tables formed an elongated ovoid shield over the anterior part of the trunk, beginning behind the head and tapering toward the sacrum, but apparently ending several vertebrae in front of the sacrum. These tables seem to have abutted with one another to form a protective pseudocarapace. There may also have been separate lateral or posterior shield elements because several thin flat pieces of ornamented bone have also been recovered (see below).
3. All vertebrae were procoelous; there is no evidence of the diplasiocoely seen in many ranoids [60].

#### Sacral vertebra

**Figure 38:**
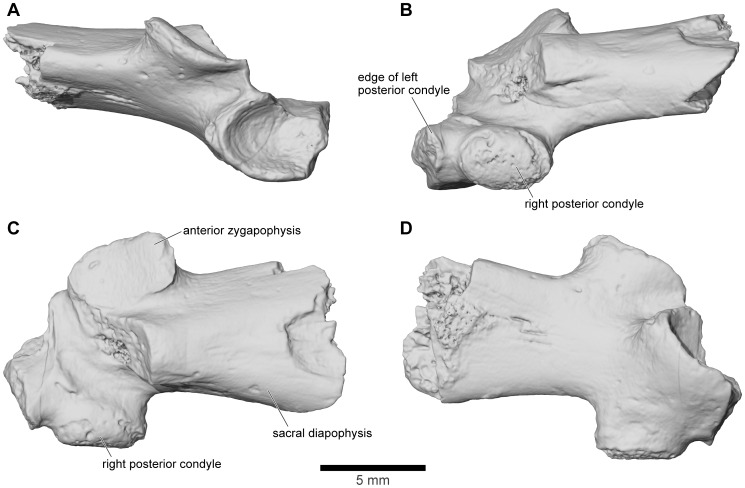
Sacral vertebra, FMNH PR 2300. **A**, anterior; **B**, posterior; **C**, dorsal; and **D**, ventral views.

FMNH PR 2003 (Fig. 38) is the right half (but missing the neural arch) of a sacral vertebra that was described and figured by Asher and Krause ([25]:fig. 1A–D) but not attributed to any particular taxon beyond Neobatrachia. Evans et al. [26] referred it to *Beelzebufo*. The bone is wide but anteroposteriorly short (midline L = ∼5.4)(Fig. 38C–D). The centrum is depressed, as in the presacral series, and has an anterior cotyle (Fig. 38A) and a bicondylar posteror margin in which the condyles are wider than they are deep (and hence ovoid). In posterior view (Fig. 38B), the long axis of the preserved right condyle is slightly oblique (dorsolateral to ventromedial) to the horizontal plane and is roughly of a size that matches well with the cotyles on the most complete urostyle (UA 9636). The sacral diapophysis is robust and extends laterally (rather than antero- or posterolaterally). It is broken distally and, as seen in dorsal view (Fig. 32), had probably lost about 25% of its length. It is dorsoventrally compressed and, as preserved, the anteroposterior length of the distal end (7.8 mm) is 137% of the basal width (5.7 mm). Assuming a continuing gradual expansion, the complete diapophysis probably had a distal end with an anteroposterior length of 1.6−2x basal length. It was therefore neither cylindrical and rod-like (as in many ranoids and some hylids [75]) nor significantly flared (e.g., as in pipids and pelobatoids [60], [76]). It matches the rather generalized condition seen in many living hyloids [59], as well as some ranoids [75], and the sacral articulation appears to correspond to the Type IIA of Emerson [77]. The anterior zygapophyses are short, broad, and almost horizontal; there are no posterior zygapophyses.

#### Urostyle

**Figure 39:**
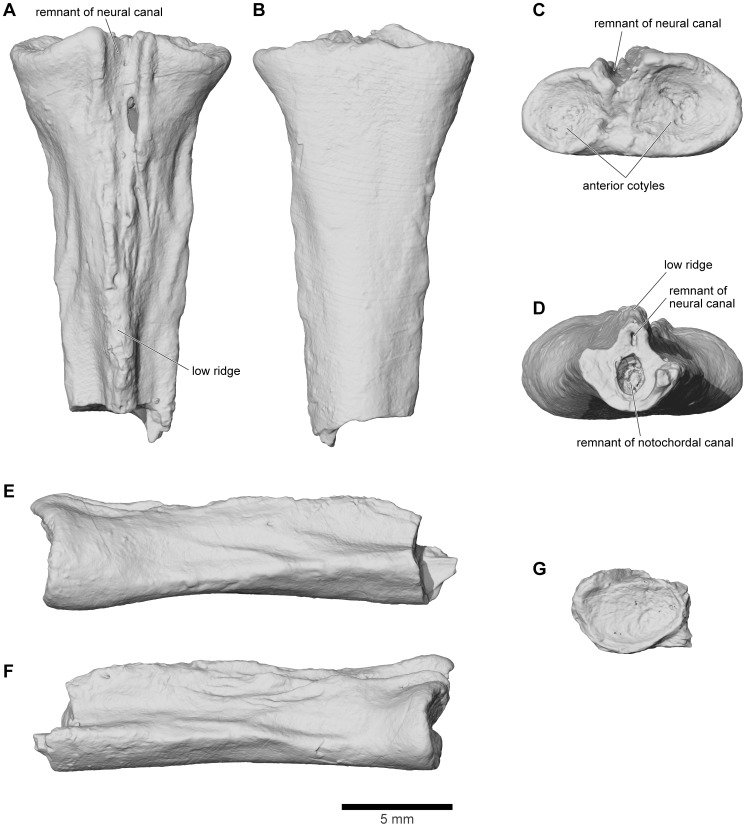
Urostyle. **A**, dorsal; **B**, ventral; **C**, anterior; **D**, posterior; **E**, left lateral; and **F**, right lateral views, UA 9636. **G**, anterior view of less complete urostyle fragment, FMNH PR 2512, representing only the right cotyle.

UA 9636 (Fig. 39A–F) is the anterior portion of a robust urostyle with a bicotylar anterior surface (matching the paired condyles of the sacrum), but no anterior zygapophyses. The bicotylar width is 10.0 mm and the length of the preserved fragment is 17.3 mm. In anterior view (Fig. 39C), the two cotyles are separated by a U-shaped groove. Small ridges on either side of the groove pass posteriorly and join at the midline in a single, low dorsal ridge (unlike the tall ridge in the comparative specimen of *Ceratophrys aurita* (LACM 163430) used in the reconstructions in Figs 1, 2, 5, and 32. However, it is possible that the small paired ridges supported a cartilaginous dorsal arch and crest. Before the two smaller ridges join to form a single ridge, a small canal representing a remnant of the neural canal enters the bone and passes posteriorly. Two canals are visible in the broken cross section at the posterior terminus of UA 9636 (Fig. 39D), the dorsal one of which, as revealed by the μCT scans, is a continuation of the neural canal, whereas a much larger ventral one marks the original course of the notochord. The urostyle lacks any trace of lateral processes.

The right anterior cotyle of a second urostyle (Fig. 39G) was recovered by screenwashing at locality MAD 98-25 and probably represents the same individual as the other cranial and postcranial elements assigned to FMNH PR 2512. UA 9636 and the partial urostyle of FMNH PR 2512 represent similarly sized individuals.

#### Osteoderms

**Figure 40:**
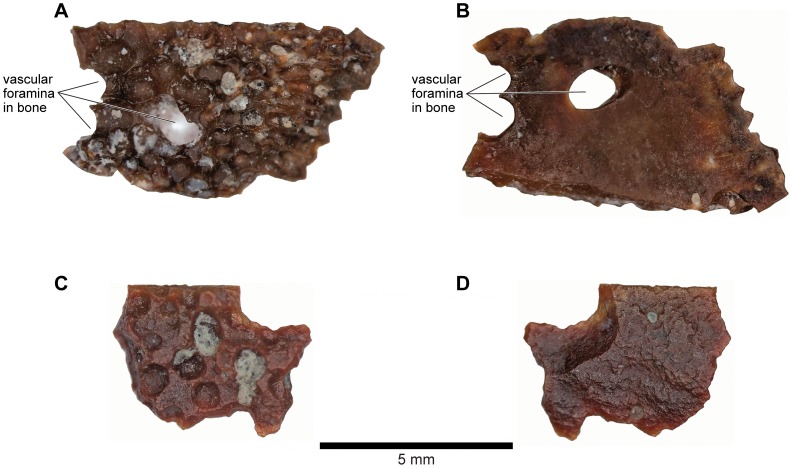
Osteoderm fragments. **A**, external; and **B**, internal views, UA 9620. **C**, external; and **D**, internal views, FMNH PR 2512.

In addition to the isolated fragments of the thick vertebral spine tables, an exceptionally thin, gently curved, and sculptured bony fragment (UA 9620) from locality MAD 93-14 may represent a more lateral osteoderm or bony shield element and may, in life, have extended protection on to the dorsolateral or posterior aspects of the trunk (Fig. 40A–B). A smaller piece of similar bony material was recovered with FMNH PR 2512 at locality MAD98-25 (Fig. 40C–D).

#### Tibiofibula

**Figure 41:**
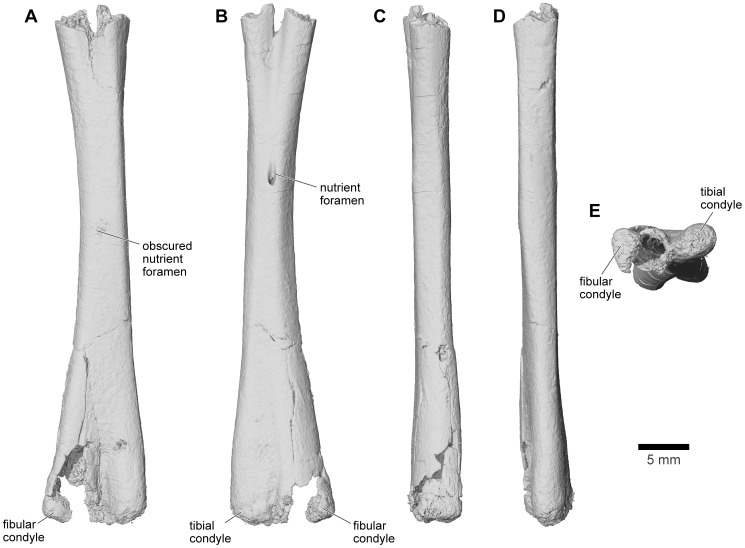
Right tibiofibula, UA 9628. **A**, anterior; **B**, posterior; **C**, lateral; **D**, medial; and **E**, distal views.

The tibiofibula, UA 9628, is represented by a large (51.3 mm in length) and robust bone from the right side (Fig. 41). Allowing for some proximal breakage, and the absence of the articular epiphyses, the original length was probably 56–62 mm (in well-ossified living frogs, the epiphyses can add 10–20% to the overall length of the tibiofibula: SEE pers. obs.). As preserved, the proximal end is transversely narrower (7.3 mm) than the distal end (10.3 mm) but the widths may originally have been similar as the proximal part of the shaft has been broken away (Fig. 41A–D). The distal end is damaged about the midline but its medial and lateral corners are complete and indicate the original terminus of the bone, with both tibial and fibular condyles (Fig. 41E). Due to the flaring of the lateral corner, the fibular margin appears concave whereas the tibial border is relatively straight. A conspicuous nutrient foramen, with a groove leading into it from above, perforates the posterior surface of the shaft (if the element were oriented vertically) just lateral to the longitudinal midline and closer to the proximal end (at approximately one-third of its preserved length; Fig. 41B). This presumably carried for a branch of the tibial artery. A smaller, obscured, foramen is present on the anterior surface (Fig. 41A). Midline grooves increasing in depth towards the (proximal) end are present on both the anterior and posterior surfaces. Similar, but shallower, grooves are developed near the distal end.

#### Tibiale-fibulare

**Figure 42:**
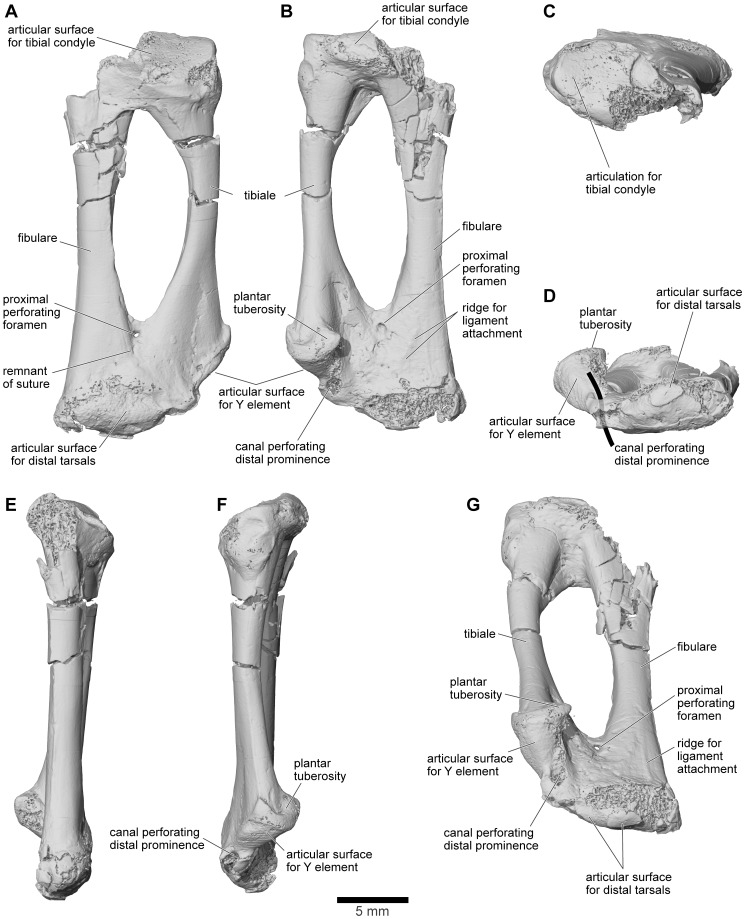
Right tibiale–fibulare, UA 9957. **A**, anterior; **B**, posterior; **C**, proximal; **D**, distal; **E**, lateral; **F**, medial; and **G**, oblique distal views.

UA 9957 (Fig. 42) is a right tibiale-fibulare that is almost complete, except for the proximal head of the fibulare, and generally well preserved. The two elements are completely fused proximally and distally to enclose a lenticular interosseus space, although a faint dorsal suture line is visible at the distal end. A small foramen perforates the sutural region close to its proximal edge (Fig. 42A–B). The proximal end of the bone (Fig. 42C) is transversely narrower (11.1 mm) than the widest part of the distal end (14.2 mm), and bears a large dorsally positioned surface that would have been extended by the epiphysis.

The fibulare is 24.6 mm long as preserved but, allowing for the missing part of the head and the articular surfaces, was originally ∼28–30 mm long. It is relatively slender proximally, with a straight shaft that expands medially in its distal one-third where it contacts the tibiale. This distal end (Fig. 42D) bears a large, convex, anterodorsally extended articular surface for the heads of the fourth and fifth metatarsals and, marked by a slight emargination medially, for a compound distal tarsal 2+3.

The tibiale is more strongly curved than the fibulare (i.e., bowed medially). It is 26.8 mm long, but is conspicuously shorter along its outer margin. The proximal end is somewhat expanded posterodorsally and bears a large, slightly concave surface that slopes gently from posterodorsal to anteromedial. With the associated joint cartilage and the fibulare, this surface would have provided articulation for the tibiofibula. The distal end is distinctly stepped, with a medial articular surface that, by comparison with that of modern anurans is likely to have met the problematic tarsal known as the Y element [78]. This surface extends onto a plantar tuberosity (Fig. 42 B,D,F–G). Together with a concavity in the plantar surface of the tibiale (Fig. 42B,D), this tuberosity creates an interosseus channel. In extant frogs, this channel accommodates the tendon of the intertarsalis muscle passing to its insertion on the Y element [79]. In modern frogs, the tibiale tuberosity also gives attachment to a transverse ligament that crosses to insert on the fibulare, thus enclosing the channel for the intertarsalis tendon (SEE pers. obs.). The attachment site for the transverse ligament may be marked on the fibulare by a weak ridge on the posterior aspect of the distal end (Fig. 42B). The position (medial or lateral) and relative diameter of the intertarsalis channel varies markedly in different frogs (SEE pers, obs.) but this variation has not been analysed in relation either to phylogenetic position or function. Distolateral to the tuberosity, the tibiale is developed into a hemispherical prominence that lacks a plantar articular surface but may have been linked dorsally to the larger surface on the fibulare. This prominence is perforated by a short canal (Fig. 42D). As the Y element supports the prehallux in anurans and the tibiale tuberosity acts as a pulley surface for the intertarsalis tendon, the prominence of the tuberosity may be an indication that this region of the foot was robust. The proportions of the tibiale-fibulare in terms of fibulare length compared to distal width (2–2.11x) are similar to those of other large-bodied walking anurans like *Calyptocephalella, Ceratophrys,* and *Pyxicephalus*) (see Table S4 in File S1).

### Phylogenetic analysis

#### Datasets and methods

We have taken a multi-dataset approach to assessing the phylogenetic placement of *Beelzebufo* within the neobatrachian radiation. One dataset consists entirely of morphological/phenotypic data and is an expanded version of that run by Báez et al. [80]. Báez et al.'s [80] dataset is composed mainly of relatively weakly ossified taxa, except for the ceratophryids and the South American Early Cretaceous taxa under consideration (notably *Eurycephalella* and *Arariphrynus*). In their analysis, *Cratia* was placed on the neobatrachian stem, but *Eurycephalella* and *Arariphrynus* grouped with ceratophryids, possibly due to shared robusticity, a problem Evans et al. [26] tried to neutralise by including strongly ossified taxa from a wide spectrum of frog families in their analyses. Therefore, in the current reanalysis of the position of *Beelzebufo*, we rescored the taxa from the [26] matrix into that of Báez et al. [80], bringing the number of included taxa up to 81 (Sections B and E in File S1). We included the putative South American fossil ceratophryids or stem-ceratophryids *Baurubatrachus* (Late Cretaceous [81]) and *Wawelia* (Miocene [82]), the putative nobleobatrachian *Uberobatrachus* (Maastrichtian [83]), and, for the morphology only analysis, the Eocene European *Thaumastosaurus* [84]. However, phylogenetically more informative material of the latter taxon has recently been described [85]. Laloy et al.'s [85] phylogenetic analysis uses the same morphological matrix as that herein. *Beelzebufo* does not group with *Thaumastosaurus*, and the European taxon is revealed to be a ranoid not a hyloid frog. During preliminary analyses, the South American *Cratia* was found to be very labile. We therefore excluded this taxon from the final analyses as it was masking considerable phylogenetic signal in the data. Inspection of the trees showed that it never nested within ceratophryids or with *Beelzebufo* so its exclusion does not bias the results of the placement of *Beelzebufo*.

For the most part, we used the character definitions as revised by Báez et al. [80], but changed that of character 42 (sacral rib proportions) to make it clearer (Section C of File S1). Character 6 (relationship of the frontoparietal fontanelle to the sphenethmoid) was particularly problematic to interpret and code, especially with hyperossified taxa in which the frontoparietals meet in the midline. We therefore omitted it from the final analyses presented, but did so only after running each set of analyses both with and without it to ensure its removal had no impact on tree topology (see Section D of File S1). Tree lengths given below are for analyses in which this character was omitted.

The second dataset is a combined evidence dataset including the morphological/phenotypic characters used in the morphology-only dataset plus genetic data from 12 genes. These data were taken from the recent large-scale analysis of Amphibia by Pyron and Wiens [27] and include nine nuclear genes and three mitochondrial genes: nuclear—C-X-C chemokine receptor type 4 (CXCR4), histone 3a (H3a), sodium-calcium exchanger (NCX-1), prox-opiomelanocortin (POMC), recombination-activating gene 1 (RAG1), rhodopsin (RHOD), seventh-in-absentia (SIA), solute-carrier family 8 (SLC8A3), tyrosinase (TYR); mitochondrial—cytochrome *b* (cyt-b), and the large and small mitochondrial ribosomal subunits (12S/16S). We followed [27] in excluding the adjacent tRNAs. Likewise, we employed the concatenated alignment of [27] that consists of 12,712 base pairs. Sequence data for the 81 taxa used above were added to the morphological/phenotypic data and an additional 21 taxa were added to the combined evidence matrix bringing the total taxon sample to 102. Increased taxon sampling was focused on basal members of the clades within Ranoidea and Hyloidea as well as on the stem of Neobatrachia, with the rationale that this sampling would improve estimation of the neobatrachian root and the basal splits within Ranoidea and Hyloidea clades that were not sampled in the morphology-only dataset. Complete taxon sampling details are provided in Section B of File S1.

Maximum Parsimony (MP) analyses were conducted using the Tree Analysis New Technology software package (TNT) v. 1.1 [86]–[87]. For the morphology-only dataset, heuristic searches were employed, performing 10,000 replicates of Wagner trees (using random addition sequences), followed by tree bisection and reconnection (TBR) holding 10 trees per TBR replicate. Zero-length branches were collapsed if they lacked support under any of the most parsimonious reconstructions (i.e., rule 1 of Coddington and Scharff [88]). For the combined evidence dataset, a more aggressive search was run using the *xmult* command. Searches were run until the shortest topology was hit 20 times. Trees saved from this search were then subjected to a final round of TBR holding 10 trees per replicate. Morphology-only trees were rooted on *Alytes obstetricans* and combined evidence trees were rooted on *Ascaphus montanus*.

Bayesian Inference (BI) trees were estimated using MrBayes v3.2 [89]. During analysis, MCMC chain convergence was assessed using the average standard deviation of split frequencies and examing trace files in Tracer [90]. Convergence to stationary was assumed for split frequencies below 0.01 and ESS values >200 [91]. For the morphological/phenotypic data we specified the Standard model (Markov k-state variable model [Mkv] with a gamma-distributed rate variation). For the molecular data in the combined analysis we ran two alternate analyses. In one, we specified a very parameter-rich model following that used by [27]. The data were partitioned by gene, codon position (for the protein coding genes), and stems and loops (for the ribosomal genes). A GTR + Γ + I model was selected, model parameters were unlinked across all partitions, and rates were allowed to vary over all partitions (ratepr = variable). For the second combined analysis, only a single molecular partition was used including all genes, which was analysed under a GTR + Γ + I model. Morphology-only trees were rooted on *Alytes obstetricans* and combined evidence trees were rooted on *Ascaphus montanus*.

### Morphology-only results

The original phylogenetic analysis of *Beelzebufo* [26] used an expanded version of a matrix constructed by Fabrezi [92]. Recently, this character set was revised by Báez et al. [80] and coded with a different set of taxa in order to investigate the relationships of three frogs from the Lower

Cretaceous (Aptian, 125.0−112.0 Ma) Crato Formation of Brazil, namely *Cratia*, *Eurycephalella*, and *Arariphrynus*. Their TNT analysis (Traditional search mode) was run with Implied Weighting [93] (k = 7), as was that of Fabrezi [92]. A preliminary rerun of Báez et al.'s [80] matrix, using the same settings, yielded a matching tree, but we also repeated the analysis with different levels of Implied Weighting (k = 1–10,15,30). Given the differences in topology, we opted to run all subsequent analyses unweighted.

**Figure 43:**
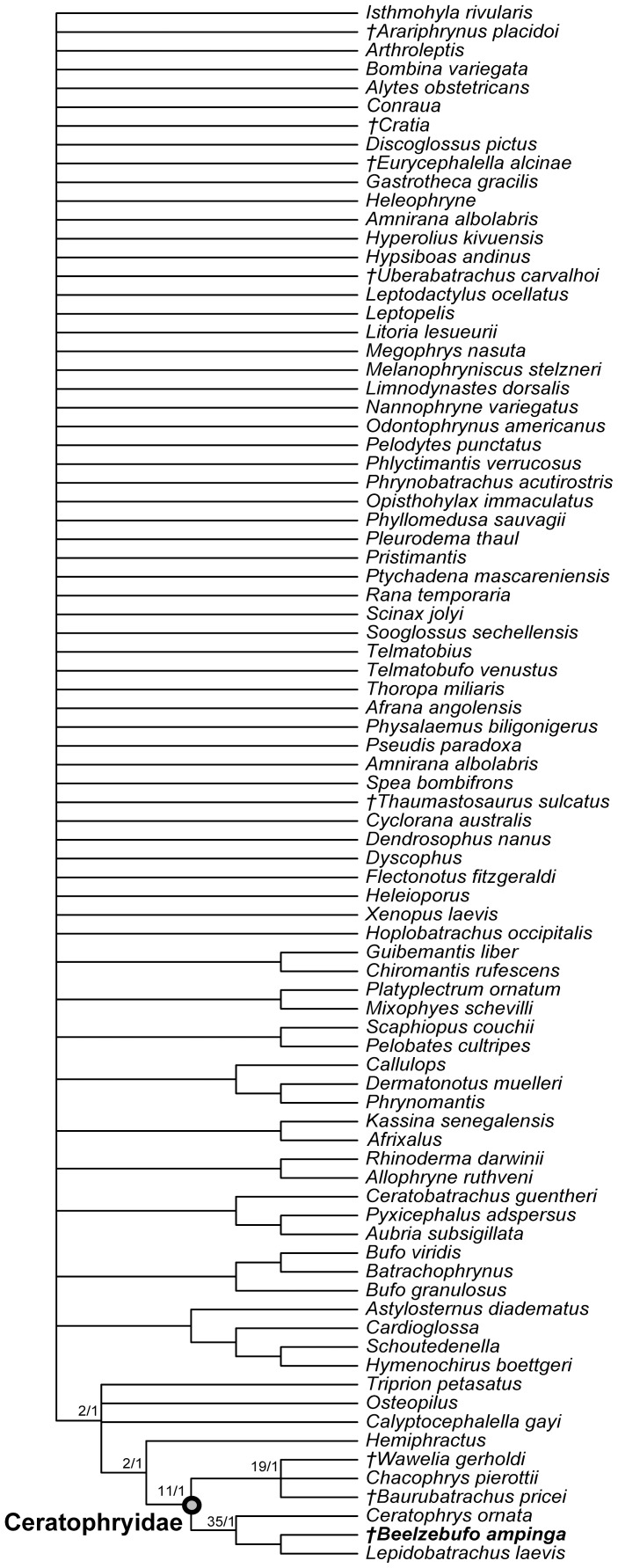
Morphology–only (maximum parsimony) strict consensus of 63,108 most parsimonious trees using full matrix, rooted on *Alytes obstetricans*. Numbers at nodes represent jackknife GC/Bremer values.

An initial MP analysis yielded 63,108 most parsimonious trees (L = 691; CI = 0.152; RI = 0.556), the strict consensus of which shows a large polytomy, in which there is almost no resolution except for ten small clades: *Rhinoderma* + *Allophryne*; *Kassina* + *Afrixalus*; *Scaphiopus* + *Pelobates*; *Guibemantis* + *Chiromantis*; *Platyplectrum* + *Mixophyes*; *Callulops +* (*Phrynomantis* + *Dermatonotus*); *Astylosternus +* (*Cardioglossa +* (*Schoutedenella* [ = *Arthroleptis*] + *Hymenochirus*)); *Ceratobatrachus* + (*Pyxicephalus* + *Aubria*); *Bufo granulosus* [ = *Incilius nebulifer*] + (*Bufo viridis* + *Batrachophrynus*); and one larger hyperossified clade: *Triprion* + *Osteopilus* + *Hemiphractus* + *Calyptocephalella* + ((*Ceratophrys* + (*Lepidobatrachus* + *Beelzebufo*)) + (*Wawelia* + *Baurubatrachus* + *Chacophrys*)). Although much of the Strict Consensus tree (Fig. 43) remains unresolved, *Beelzebufo* is placed with Ceratophryidae (and the Miocene *Wawelia* and the Cretaceous *Baurubatrachus*), as in previous phylogenetic analyses [26], [29], with *Hemiphractus*, *Calyptocephalella*, *Osteopilus*, and *Triprion* as proximate outgroups. The hyperossified hyloid clade around *Beelzebufo* has weak jackknife support (GC = 2) and a Bremer value of 1.

Given the possibility that the hyperossified clade is an artificial grouping of robust hyloids that lack distinctive characters of the type that place hyperossified ranoids, pelobatoids, and bufonids with their less ossified relatives, we ran a second MP morphology-only analysis omitting the characters most often associated with high levels of ossification, namely characters 1–5, 7, 9–11, 13, 15, 22, 25, and 38, although we accept that some of these are not universally linked to hyperossification. We left character 48 (dermal armour) in the analysis as it links extant ceratophryids (although it is absent in some species [74]), but coded *Beelzebufo* as (?) so as not to assume homology. This analysis found 2,648 trees (L = 507, CI = 0.168; RI = 0.584). The resulting strict consensus tree (Fig. S1 in File S1) places *Beelzebufo* within Neobatrachia crownward of *Heleophryne* and Sooglossidae, and within a clade that encompasses extant ceratophryids, *Wawelia*, and *Baurubatrachus*. In this analysis, *Triprion* and *Osteopilus* grouped more realistically with hylids, and both *Hemiphractus* and *Calyptocephalella* were separated from ceratophryids. Most of the clades still lack high support.

To test the possibility that hyperossification alone was sufficient to group all hyperossified frogs regardless of ranoid or hyloid affinities, we ran a third MP morphology-only analysis where we included only characters associated with hyperossification. Doing this resulted in 99,999 trees (L = 101), the strict consensus of which is nearly a complete star phylogeny (Fig. S2 in File S1) with only two clades resolved: *Xenopus* + *Hoplobatrachus* and *Beelzebufo* + *Lepidobatrachus* + *Ceratophrys*. The ceratophryid clade is supported by a single synapomorphy—a sutured or partially fused midline contact between the frontoparietals.

To test the possibility that a combination of missing data and the inclusion of *Baurubatrachus* and *Wawelia* were drawing *Beelzebufo* into the ceratophryid clade, we ran a fourth MP morphology-only analysis in which firstly *Wawelia* (Fig. S3 in File S1), then *Baurubatrachus* (Fig. S4 in File S1), and then both South American fossil taxa (Fig. S5 in File S1), were deleted. Removal of *Wawelia* and *Baurubatrachus* individually resulted in trees with identical scores, the strict consensus of which (although differing in the level of resolution) each retained a ceratophryid clade containing *Beelzebufo*. Removing both taxa yielded 6,376 trees (L = 691; CI = 0.152; RI = 0.556), the strict consensus of which is reduced compared to the total analysis. Nevertheless, it retained a clade (albeit again weakly supported) comprising the three living ceratophryids with *Beelzebufo*.

**Figure 44:**
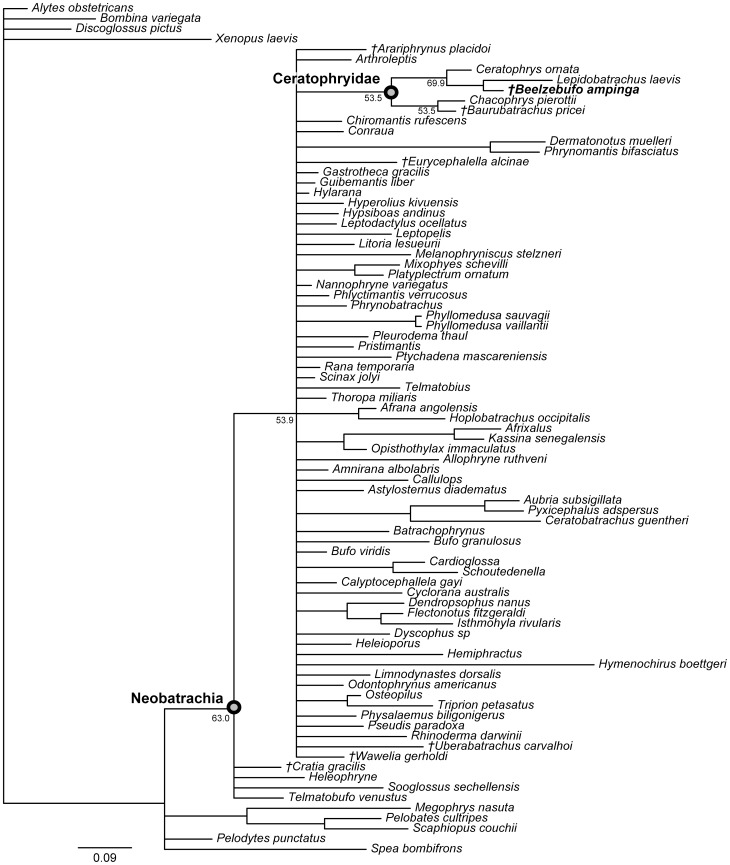
Morphology–only Bayesian inference tree, rooted on *Alytes obstetricans*. Numbers at nodes are posterior probabilities.

The Bayesian Inference tree for the morphology-only dataset (Fig. 44) is slightly more resolved than the MP tree. The BI analysis was run for 10 million generations and the first 25% were discarded as “burn-in.” The Neobatrachia node is recovered with *Cratia gracilis*, *Heleophryne natalensis* (* = Hadromophryne*), *Sooglossus sechellensis*, and *Telmatobufo venustus* outside of the node containing all other neobatrachians. Nine small clades are recovered among neobatrachians plus a Ceratophryidae clade containing the same set of taxa as in the MP tree except for *Wawelia*, which is unresolved among most neobatrachians. The node containing *Ceratophrys*, *Lepidobatrachus*, and *Beelzebufo* is supported by a relatively low posterior probability (69%), but this is similar to support found by [29] for the same node as well as the relatively low bootstrap support found by [27] for the *Lepidobatrachus* + *Ceratophrys* node (65%) in the maximum likelihood (ML) analysis, which contained no fossils.

### Combined evidence results

**Figure 45:**
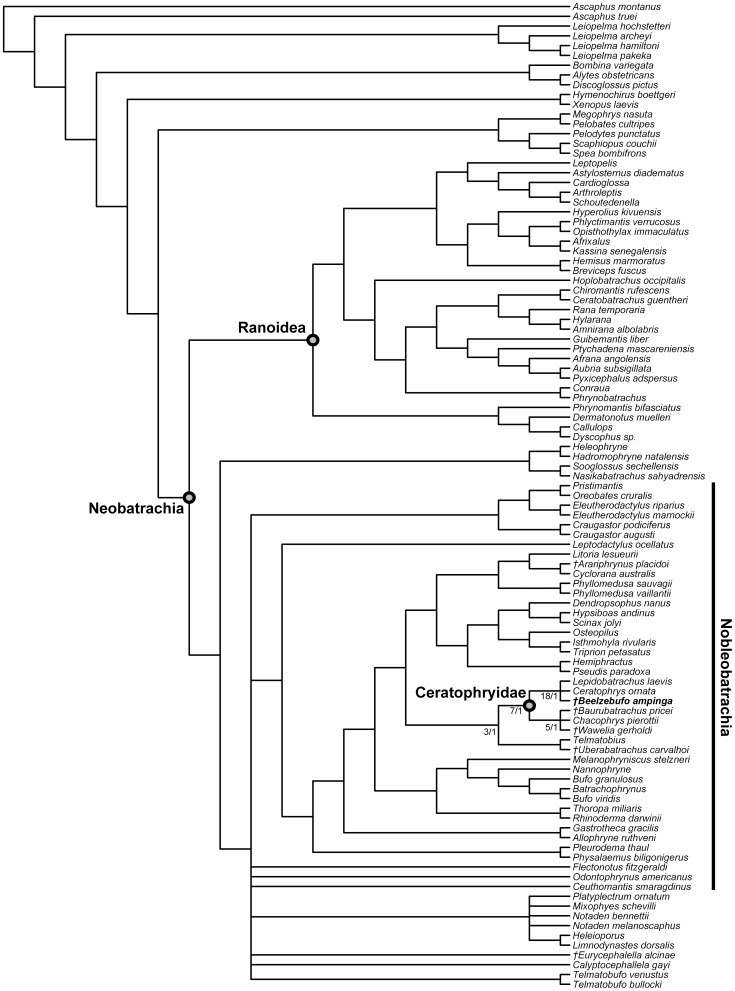
Combined evidence (maximum parsimony) strict consensus of four most parsimonious trees, rooted on *Ascaphus montanus*. Numbers at nodes represent jackknife GC/Bremer values.

Maximum Parsimony analysis of the combined dataset resulted in four most parsimonious trees (L = 36,014; CI = 0.279; RI = 0.410). The strict consensus (Fig. 45) is well resolved and shows relationships broadly congruent with those of [27]. A monophyletic Neobatrachia is recovered as well as monophyletic Ranoidea and Hyloidea clades. Detailed relationships among the family-level clades of Ranoidea and Hyloidea differ from those of [27], but this is not surprising given that our analysis heavily down-sampled from the taxon-sampling regime of [27] (ntax = 102 versus ntax = 2,871, respectively). A detailed description of the results are beyond the scope of this paper, and we will focus only on points pertinent to *Beelzebufo* and the other fossil forms included in the analysis.

**Figure 46:**
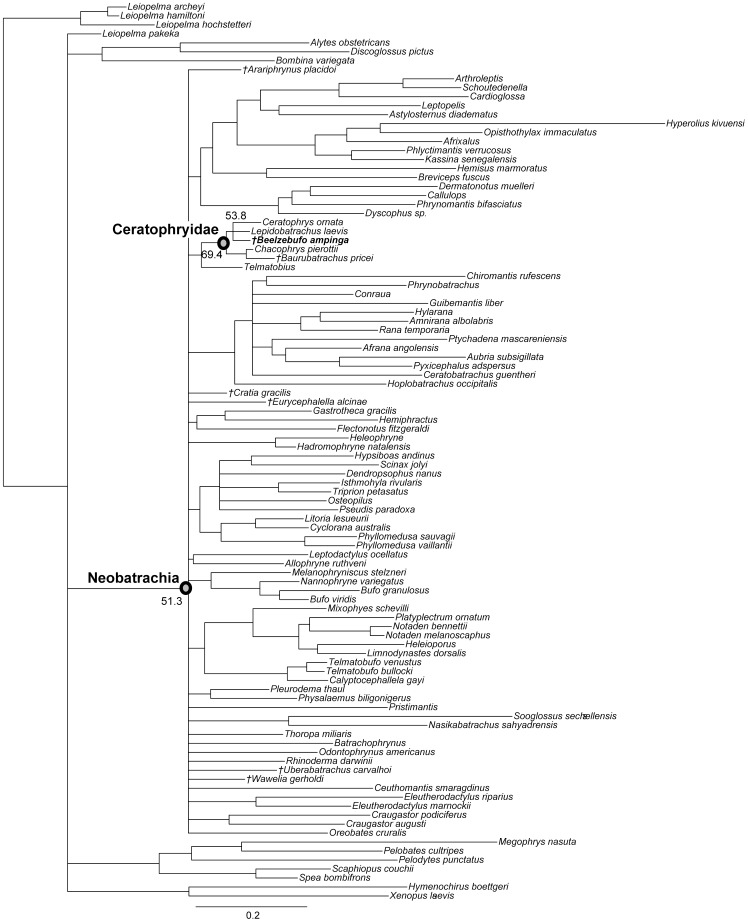
Combined evidence Bayesian Inference tree, rooted on *Ascaphus montanus*. Note that as the *Ascaphus* species lay on a very long branch at the base of the tree, they have been omitted to reduce figure size. Numbers at nodes are posterior probabilities.

Bayesian Inference (of both the highly partitioned and two-partition datasets) produced trees much less resolved than the MP analysis (Fig. 46). The two-partition analysis was run for 55 million generations and the highly partitioned analysis was run for 90 million generations. Both reached stationarity based on split frequencies and ESS values in Tracer. The recovered trees from both BI analyses show very similar results. A monophyletic Neobatrachia is recovered and the outgroups are well resolved. Relationships among neobatrachian taxa are largely unresolved but a number of the more derived family-level hyloid and ranoid clades are recovered. The large polytomy among neobatrachians may be driven in part by the inclusion of the fossil taxa, which lack data for the vast majority of characters in the matrix. Lack of support near the centre of the tree in BI phylogenies, especially when incomplete taxa are included, has been noted by previous authors [94]–[96].

In both the MP and BI combined evidence trees, a ceratophryid clade was recovered consisting of *Ceratophrys*, *Lepidobatrachus*, *Beelzebufo*, *Chacophrys*, and *Baurubatrachus*. In the MP analysis *Wawelia* is also recovered as a ceratophryid. *Telmatobius* + *Uberabatrachus* is the sister taxon to Ceratophryidae. This is in fact not much different from the ML results of [27], given that our analysis does not sample any odontophrynid, batrachylid, cycloramphid, hylodid, or alsodid taxa, which are all more derived members of the ceratophryid + telmatobiid clade in [27]. Support metrics for Ceratophryidae are low: BI posterior probabilities of 69% (two partition analysis) and 66% (multi-partition analysis); and low jackknife (GC = 14) and Bremer support ( = 1) in the MP analysis. However, support for the similarly composed clade in [27], *Ceratophrys ornata* + *Lepidobatrachus* + *Chacophrys*, is low in their ML analysis (65% bootstrap), thus indicating that it is not the inclusion of *Beelzebufo* or other putative fossil ceratophryids that is reducing support for the node.

Sensitivity analyses were run using the combined evidence dataset (see Section D of File S1). Like those conducted for the morphology-only dataset, we checked to see if: 1) exclusion of morphological characters associated with hyperossification affected the placement of *Beelzebufo* (Fig. S6 in File S1); 2) exclusion of *Baurubatrachus* affected the placement of *Beelzebufo* (Fig. S7 in File S1); 3) exclusion of *Wawelia* affected the placement of *Beelzebufo* (Fig. S8 in File S1); and 4) exclusion of *Baurubatrachus* and *Wawelia* affected the placement of *Beelzebufo* (Fig. S9 in File S1). In all cases, *Beelzebufo* continued to group with *Ceratophrys* and *Lepidobatrachus*.

### Phylogenetic results summary

Regardless of the data type or method of tree reconstruction/estimation (i.e., morphology-only or combined evidence; MP or BI), phylogenetic analyses always find the Late Cretaceous *Beelzebufo* from Madagascar and the Late Cretaceous *Baurubatrachus* from Brazil in a ceratophryid clade with extant members *Ceratophrys*, *Lepidobatrachus*, and *Chacophrys*. *Chacophrys* and *Baurubatrachus* are sister taxa in all analyses and *Beelzebufo*, *Ceratophrys*, and *Lepidobatrachus* form a clade in all analyses. MP, whether with the morphology-only or with the combined dataset, always recovers the Miocene *Wawelia* with the *Chacophrys* + *Baurubatrachus* clade.

The low topological resolution among frogs in the morphology-only analysis indicates a large amount of character conflict present in the dataset. Exclusion of characters associated with hyperossification improves resolution (but returns atypical clades) suggesting that the prevalence of robust frogs across various neobatrachian clades may be resulting in numerous equally parsimonious topologies perhaps owing to insufficient sampling of morphological features sufficient to parse these disparate clades. Combining morphological and molecular data results in greater resolution among neobatrachian clades.

Most of the morphological features that support the monophyly of Ceratophryidae + Telmatobiidae, and Ceratophryidae and its subclades, relate to being a robust hyperossified frog. Therefore most of the morphological features that place *Beelzebufo* and the other fossil taxa with ceratophryids are features of hyperossification. A single morphological feature, a skull roof in the orbital region that is less than a quarter of the orbital width (character 38.1), supports Ceratophryidae + Telmatobiidae. Ceratophryidae is supported by ten morphological synapomorphies to the exclusion of *Telmatobius* + *Uberabatrachus*. These include cranial exostosis (character 2.1), no dorsal exposure of the sphenethmoid (character 7.0), the presence of a parieto-squamosal arch (character 9.1), the otic ramus of the squamosal overlapping the crista parotica (character 10.1), monocuspid teeth (character 13.1), the anterior process of the vomer not reaching the maxillary arch (character 19.1—unknown in *Beelzebufo*), odontoids on the lower jaw (character 25.1—unknown in *Beelzebufo*), presence of an anterolateral process on the hyloid plate (character 28.1—unknown in *Beelzebufo*), presence of a femoral crest (character 68.1—unknown in *Beelzebufo*), and a hypertrophied, spade-like distal element on the prehallux (character 72.3—unknown in *Beelzebufo*). *Beelzebufo* + *Lepidobatrachus* + *Ceratophrys* is supported by high neural spines on the anterior presacral vertebrae (character 38.1—convergently shared with other hyperossified lineages such as *Hemiphractus*, *Pseudis*, *Bufo granulosus* [ = *Incilius nebulifer*], *Odontophrynus*, *Calyptocephallela*, *Ceratobatrachus*, and *Pyxicephalidae*); and the presence of a dorsal shield (character 48.1—a feature uniquely present among these three taxa).

Exclusion of characters associated with hyperossification does not overturn the phylogenetic placement of *Beelzebufo*. In both the morphology-only and combined analyses, five synapomorphies (characters 16.1, 19.1, 28.1, 31.2, and 72.3) unite ceratophryids. Only one of these traits is preserved in *Beelzebufo* (character 16.1), which is the absence of a palatine shelf on the premaxilla.

The morphological support for many of the deeper nodes within and including Neobatrachia is weak, although this may be more of a reflection on the admittedly limited morphological character sampling in the present matrix. Only two morphological characters support Neobatrachia monophyly, the procoelous centra in the posterior-most presacral vertebrae (character 37.1) and the presence of an anterior lamina on the scapula (character 58.1—unknown in *Beelzebufo*). *Beelzebufo* does preserve a well-developed zygomatic ramus of the squamosal that articulates with the maxilla (character 11.2) and contact between the pterygoid and parasphenoid (character 22.1). These features serve to nest *Beelzebufo* up within Nobleobatrachia within a clade containing Hemiphractidae, Phyllomedusinae, and Hylinae.

## Discussion

The material attributed to *Beelzebufo* includes articulated, associated, and isolated elements and taken together, their size, robusticity, consistency of morphology, and sculpture pattern argue for referral to a single, large, possibly dimorphic taxon. There is evidence of one or more smaller frogs in the Maevarano Formation faunal assemblage, but these will be discussed elsewhere. The new material of *Beelzebufo*, in combination with the original described specimens [25]–[26], confirms that it was a large, heavily armoured anuran that broadly resembled living ceratophryids in its morphology. Nonetheless, the questions raised with respect to biogeography [32] and divergence times [29] require a reconsideration of these issues, in conjunction with an assessment of lifestyle based on the skeletal specialisations in the context of paleoenvironmental reconstructions of the Maevarano Formation.

### Body Size

**Figure 47:**
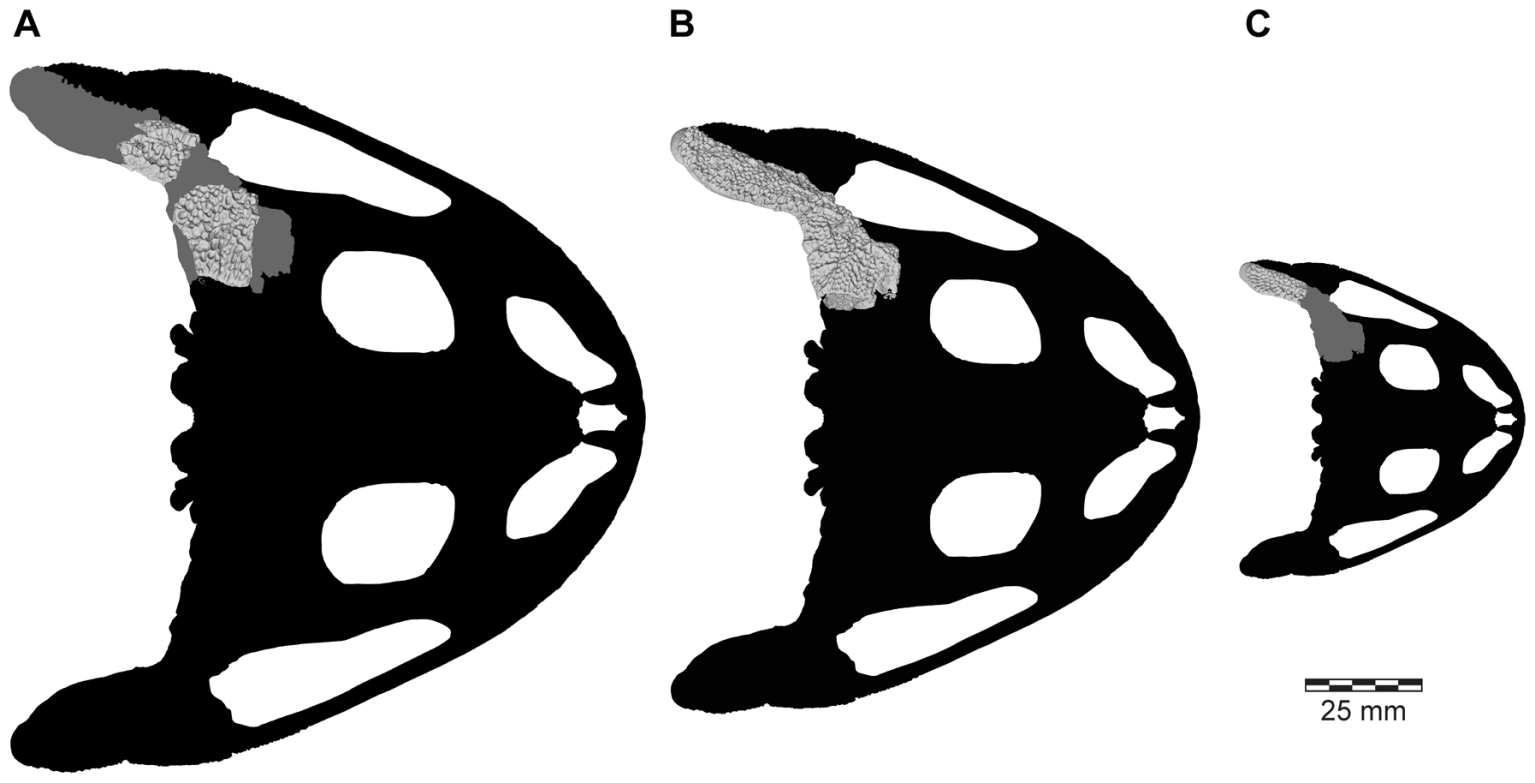
Intraspecific size range of *Beelzebufo ampinga*. Left squamosals of **A**, UA 9629; **B**, FMNH PR 2512 (reversed for comparison); and **C**, UA 9614, all in dorsal view. Skull silhouettes based on Fig. 4B and scaled by variation in size range of selected squamosals. Assuming isometric growth trajectory, individual represented by UA 9629 would have been about 20 percent larger than FMNH PR 2512, and that represented by UA 9614 about half the size of FMNH PR 2512.

**Figure 48:**
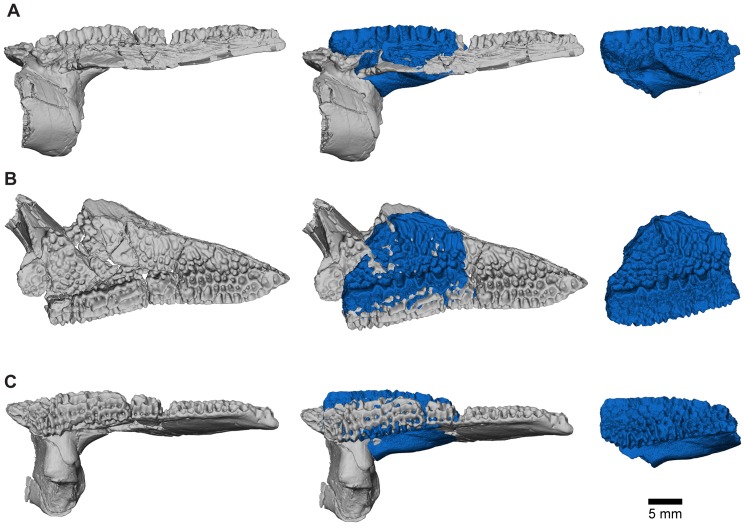
Intraspecific differences in pattern of bone growth. **A**, dorsal; **B**, lateral; and **C**, ventrolateral comparisons of digital volume of relatively robust quadratojugal fragment UA 9639 (blue, at right) with that of quadrate-quadratojugal of FMNH PR 2512 (grey, mirror-imaged, at left). Integrated volumes (centre) show relatively greater medial and lateral development of bone growth in UA 9639, particularly in quadratojugal buttress.

Reconstructing snout-to-vent length (SVL) is difficult without a complete axial skeleton, pelvis, or anterior cranium. However, the reconstruction in Figures 1–2, based on FMNH PR 2512 and the postcranial skeleton of *Ceratophrys aurita* (LACM 163430), yields an estimated SVL of 193 mm and a posterior skull width of ∼129 mm. If growth was isometric (but see below), larger individuals represented by the squamosal UA 9629 (Fig. 47A) could have exceeded this by 20% (SVL = ∼232 mm; skull width ∼154 mm). This is lower than the size estimates in [26] but is still at the upper end of the size range for robust-bodied extant anurans like the African Bullfrog, *Pyxicephalus adspersus* (up to 245 mm [97]) and the Marine Toad, *Rhinella marina* (100–238 mm [98]). Furthermore, as the cranial sutures were still open in these *Beelzebufo* individuals, there may have been a potential for further growth. However, it is clear that skeletal growth and skeletal maturation varied between individuals, and that some completed their growth at a smaller size than others (Fig. 47). In FMNH PR 2512, for example, the median sutures between the frontoparietals and otoccipitals are completely closed, whereas in the similar-sized UA 9675 these median sutures remain open. Conversely, the lateral skull sutures remain patent in FMNH PR 2512, including that between the squamosal and quadratojugal, whereas in the similar-sized FMNH PR 2536 (Fig. 18F–G) the suture between these elements has closed without trace. There are also differences between individuals in the pattern of bone growth. The largest squamosal (UA 9629) scales roughly isometrically against FMNH PR 2512. The bone is thicker but not unexpectedly so for its size. By contrast, the quadratojugal fragment UA 9639 is of similar outline size to the corresponding element of FMNH PR 2512 (Fig. 48), but is significantly thicker with a massive quadrate buttress. Rather than continuing to increase in overall size, despite the patent sutures, this individual appears to have become heavier and more robust. These differences may be indicative of sexual dimorphism and/or perhaps different growth/maturation rates relating to environmental conditions. Studies on extant frogs have shown that pre- and postmetamorphic growth, and skeletal maturation, are influenced by factors such as food availability, temperature, and seasonal water availability [99]–[102]. Growth may continue after sexual maturity is reached, but its rate and ultimate cessation depend on seasonal conditions and the sex of the individual [103], females attaining larger size in around 90% of anuran species [104]. Large frogs also tend to be relatively long-lived (e.g., ∼25 years for *Rhinella marina*; ∼16 years for *Pyxicephalus adspersus*; 12–16 years for *Ceratophrys* spp. (AnAge database build 12 [105]), and this may also have been the case for *Beelzebufo*.

### Functional anatomy and lifestyle

In the latest Cretaceous (Maastrichtian), northern Madagascar lay close to 30° south latitude [106]–[109], within the high-pressure, subtropical arid belt. Consistent with this, the palaeoenvironment of the Maevarano Formation has been reconstructed as semi-arid and highly seasonal, with prolonged dry periods interspersed with sporadic heavy rains [41]–[43], [109]–[110]. The dry season probably yielded severe drought conditions, with animals attracted to the desiccating riverbeds and remaining pools of water [110]–[111]. This was a challenging environment for a large amphibian, but is comparable to that sometimes experienced by extant *Ceratophrys* (South America) and *Pyxicephalus* (Africa). In fact, anuran hyperossification has frequently been linked to life in arid or seasonally arid environments of this kind [60], [112]–[116].

Today ceratophryids are found throughout much of South America, in warm, dry, non-forested environments with ephemeral pools [59], [117]–[119], most notably in the Chaco region of Argentina [102], [120]. Their thick dry skin, globular shape, short limbs, and large size are advantageous under these conditions [59], as is a fast rate of larval development [121]–[122]. Many of these features also apply to *Beelzebufo ampinga*, notably large size, thick skin (as suggested by the coarse cranial and vertebral sculpture), short deep body (from vertebral size and structure), and short limbs (relatively short, robust distal limb elements). Moreover, the open sutures, even in large individuals, and the size range of individual bones (e.g., Fig. 47) are suggestive of extended postmetamorphic growth, like that of *Ceratophrys* [122]. Leaving aside the question of relationship, *Ceratophrys* provides a reasonable living model, as does the African Bullfrog, *Pyxicephalus adspersus*, which, though unrelated to *Ceratophrys* (ranoid v. hyloid), resembles it both behaviourally and, in a functional sense, morphologically (large size, globular shape, large robust skull, unicuspid teeth), although there are many important differences [72]. Like *Ceratophrys* and *Pyxicephalus*, *Beelzebufo* is interpreted as a predominantly terrestrial anuran. Its occurrence in the Lac Kinkony Member of the Maevarano Formation suggests that it inhabited coastal/paralic as well as the more inland environments represented by the Anembalemba and Masorobe members.

Large extant hyperossified anurans like *Ceratophrys* and *Pyxicephalus* are typically aggressive, ambush predators that take a range of invertebrate and vertebrate prey, including other anurans, small mammals, lizards, and birds [123]–[124]. They are not built for speed and conserve energy by using a sit-and-wait strategy. One study of *Cerataphrys cornuta* [123] found that 53% of the prey (by volume) was vertebrate, with small mammals and anurans forming the major component. The strong ceratophryid bite is correlated with the possession of strong adductor muscles acting in conjunction with a robust skull, posteriorly placed jaw joints, unicuspid teeth, and the presence of fang-like odontoids on the lower jaw [122], [124], as well as the stabilizing effects of a strong premaxillary-maxillary articulation [60] and robust contacts between the maxilla and the nasal on the one hand and squamosal and quadratojugal on the other [125]. Most of these cranial features are found in *Beelzebufo*, which also has a wide head. The transverse width across the occipital condyles in the skull of FMNH PR 2512 is 17.1 mm and, as reconstructed, the biquadrate skull width is ∼106.3 mm ( =  6.22x transcondylar width). This is greater than that of most hyperossified extant taxa examined (e.g., *Pelobates cultripes*, 3.92x; *Calyptocephalella gayi*, 4.79–5.23x; *Pyxicephalus adspersus*, 5.02–5.03x; *Rhinella marina*, 5.65x), and is comparable to *Litoria australis* (6.31x) and *Ceratophrys* spp. (5.23–6.67x) given that the transcondylar width in FMNH PR 2512 is somewhat exaggerated by dorsoventral crushing and midline displacement.The skull of *Lepidobatrachus asper* is proportionally even wider (biquadrate width 6.79x transcotylar width) (Table S5 in File S1). Among extant frogs, disproportionately large, wide skulls equate with large gape and the consumption of vertebrate prey [126], which further supports the interpretation of *Beelzebufo* as an aggressive vertebrate predator like *Ceratophrys* and *Pyxicephalus* [60], [112], [126].

Given the overall size of *Beelzebufo*, its tibiofibulae were relatively short (7.27–8.05x length of PS4, allowing for the fact they come from different individuals), with similar proportions to those of some bufonids, pelobatids and microhylids (Table S4 in File S1), suggesting that *Beelzebufo* was rather short-limbed, using walking rather than saltation as its main locomotor mode, which is also consistent with its inferred heavy body. The proportions of the tibiale-fibulare, in terms of fibulare length compared to distal width (2.00–2.11x), are consistent with this interpretation, and again resemble those of other large, short-bodied walking anurans like *Calyptocephalella*, *Ceratophrys*, and *Pyxicephalus* (see Table S4 in File S1). Thus, like *Ceratophrys* and *Pyxicephalus*, *Beelzebufo* was probably slow-moving (large head, deep and globular armoured body, short legs) and reliant on ambush [26].

In living anurans, the absence of a posteriorly directed otic ramus on the squamosal generally reflects the absence of a tympanic membrane [59], although the reverse is not always the case (some frogs that lack a tympanum retain the otic process – Z. Roček pers. comm. 2013). Consequently, *Beelzebufo* probably did not have a tympanic membrane. It did have a columella, but the width and shape of the skull render it unlikely that the columella, even with a cartilaginous distal extension, could have reached the skin and, as in some living anurans (e.g., *Bombina* [68]), the columella may have ended in cranial soft tissue. In frogs, loss of the tympanic membrane occurs most often in aquatic specialists and burrowers [68].

In many anurans, the vertebral neural spines are short, posterodorsally directed processes at the posterior edges of the vertebrae. Tall vertical anterior spines like those of *Beelzebufo* are relatively uncommon and are suggestive of well-developed epaxial musculature, an interpretation that, as noted above, would be consistent with the deep recesses and ridges in the occipital region. Tall neural spines also occur in *Ceratophrys*, *Lepidobatrachus*, and *Pyxicephalus* ([80], SEE, pers. obs.), but they are not as robust in cross-section and lack spine tables. However, most species of *Ceratophrys* and *Lepidobatrachus* also have a dorsal dermal shield [61], [74], [92], [127]. The bony plates comprising this shield typically rest on the flattened tips of the vertebral neural spines and are attached to them by ligaments [74], although they may become co-ossified with the neural spines in some fully developed individuals (*C. cranwelli*, Z. Roček, pers. comm. 2013). Like the dorsal shield of ceratophryids, the spine tables of *Beelzebufo*, or at least their dorsal layer, presumably formed as condensations in the dermis and then fused to the vertebral neural spines during development. Among living anurans, the only other taxon with an arrangement consistently resembling that of *Beelzebufo* is the tiny, but strongly ossified South American *Brachycephalus* [128]–[129]. In that genus separate paravertebral plates form and spread inward, covering and then fusing to the neural spines. These dermal plates are undoubtedly protective but may also, like cranial exostosis, have a role in water conservation [92], [127]. In *Beelzebufo*, the anterior spine tables are combined with tall, thick neural spines that imply the presence of strong interspinal muscles and ligaments. Together with the fusion of the first two vertebrae, this could have yielded a stiff vertebral column that was resistant to dorsoventral buckling. Emerson [130] related this type of adaptation in the genus *Hemisus* to head-first burrowing but *Hemisus* is strikingly different from *Beelzebufo* in being narrow-headed. However, axial stiffening may not be restricted to head-first burrowers as Radhakrishan et al. [131] reported that the Indian *Nasikabatrachus* contracts its epaxial muscles (which bulge out on either side of the vertebral column) to stiffen the body during hind limb burrowing.

Finally, the most unusual aspect of the skeleton of *Beelzebufo* is the development of the large posterolaterally directed quadratojugal-squamosal flanges on the skull. Typically, the ventral components of the anuran pectoral girdle (clavicles, coracoids) meet in either a fixed (firmisternal) or overlapping (arciferal) contact below the anterior thorax. Of the dorsal components, the ossified scapulae are usually positioned just behind the skull with their upper margins level with, or just below, the transverse processes of the anterior presacrals. They are extended dorsally by cartilaginous suprascapulae that curve toward the dorsal midline. The girdles are suspended by muscles (e.g., serratus, scapularis) from the transverse processes of the anterior presacral vertebrae (typically PS3–4). In *Beelzebufo*, the pectoral girdles are unknown but the dorsolateral skull flanges extended more than 30 mm posterior to the occipital condyles, taking them beyond the level of PS4. It is clear from the exostosis on all but the posterior tips of the flanges that they remained in close contact with the skin covering the rest of the cranium. In the process of making the reconstruction in Figures 1 and 2, it became clear that the flanges must have overlapped the scapulae laterally. Moreover, the suprascapulae would have been limited to a dorsolateral position due to the expanded spine tables. An analogous, though less extreme, condition exists in *Ceratophrys* in which the posterolateral margins of the skull also slightly overlap the scapulae because the quadrates are positioned well behind the occiput. Movement of the humerus is not restricted in *Ceratophrys* as the glenoid fossa lies below the level of the skull, but it is possible that the large posterolateral flanges in *Beelzebufo* may have affected forelimb movements to some degree.

In conclusion, much of the morphology—loss of a tympanic membrane, long acoustic meatus, cranial exostosis, short-limbed globose body shape, tall neural spines (and, by implication, strong epaxial muscles), expansive spine tables [60], [130], [132]—is consistent with the hypothesis that *Beelzebufo* was at least partly adapted to burrowing, a common strategy for terrestrial anurans in an arid or seasonally arid environment [122]. Although the cranium of *Beelzebufo* is robustly built, with firm connections between the components, its width and possibly limiting posterolateral flanges argue against head-first burrowing [130] and probably also forelimb burrowing, leaving hind limb burrowing with a stiffened back, as described for *Nasikabatrachus* [131], as the most likely option. This is also the most common burrowing technique amongst living anurans [130], [133]) and anurans with short tibiofibulae tend to both walk (rather than hop) and dig [130]. Our measurements of tarsal (tibiale-fibulare) proportions (length/width, Table S4 in File S1) show that *Beelzebufo* had neither the very short wide tarsal bone of specialised burrowers (e.g., *Rhinophrynus*, *Rhombophryne*, *Scaphiophryne*), nor the elongate element of saltators (e.g., hylids), and most closely resembles the tarsal proportions of *Calyptocephalella, Limnodynastes tasmaniensis, Platyplectrum spenceri, Litoria platycephala*, and *Kaloula pulchra*, the latter three of which are seasonal burrowers. Ceratophryines have a keratinised pad over the first metatarsal as an adaptation to digging [102] and Emerson [130] figures the areas adjacent to the first metatarsal, and overlying the prehallux and its articulation, as being important focal points in hind limb burrowing. We do not have the pes of *Beelzebufo* but the articular region for the Y element on the tibiale-fibulare is prominent. *Beelzebufo* may have spent the hottest, driest periods fully or partially buried, possibly within a cocoon, as do many arid-adapted living anurans [122], [130], [133], emerging to feed and reproduce during periods of wetter and/or cooler conditions.

### Phylogenetic relationships

Together, the features of its vertebral column preclude attribution of *Beelzebufo* to leiopelmatids (amphicoely, monocondylar sacro-urostylar joint, urostyle with transverse processes [60]), ‘discoglossids’ (Costata sensu [9]: opisthocoely, urostyle with transverse processes [60]), or pipids (opisthocoely, expanded sacral diapophyses, fused sacro-coccygeal joint), and make attribution to pelobatids (expanded sacral diapophyses, fused or monocondylar sacro-coccygeal joint), pelodytids (dilated sacral diapophyses), or the extinct palaeobatrachids (synsacrum, dilated sacral diapophyses) unlikely [10], [60], [76]. They also rule out fossil groups such as the Cretaceous Asian gobiatines (amphicoely, expanded sacral diapophyses, transverse processes on urostyle [134]–[136]).

As noted above, very few morphological characters have been identified as diagnostic for Neobatrachia (e.g., neopalatine bone present, fusion of distal carpal 3 to the others, complete separation of sartorius from semitendinosus, accessory head of adductor longus, no parahyoid [9], [60], [112], [137]), and Frost et al. [9] considered only the sartorius character to be robust. None of these can be coded for *Beelzebufo* (unless the partial facet on the maxilla is for the neopalatine), but every analysis we ran placed *Beelzebufo* within Neobatrachia crownward of both heleophrynids and sooglossids. This is supported by a combination of features (e.g., no transverse processes on the urostyle, T-shaped parasphenoid, procoely, bicondylar sacro-urostylar joint [80], [138]). The presence of a well-developed lateral chamber in the ear may also support this position [68], as may holochordal vertebral centra [76]. Among neobatrachians, *Beelzebufo* differs from the ‘basal’ African heleophrynids in lacking transverse processes on its urostyle [10], [59] and from sooglossids in the presence of a bicondylar rather than monocondylar sacro-urostylar joint [10]. *Nasikabatrachus* [139] is medium-sized (∼68 mm SVL) and well ossified, and its shared common ancestor with sooglossids is likely to have been on the Indo-Madagascar plate when it separated from the rest of Gondwana (see below, Biogeography). Very few details of the skull and skeleton have been described and little morphological detail is visible on the published X-ray [139], making comparison difficult and precluding inclusion in the morphology-based analysis. Nonetheless, the X-ray images do show that *Nasikabatrachus* lacks the posterolateral skull flanges and armoured vertebral spine tables found in *Beelzebufo*, and it also differs in having a relatively smaller head and small orbits.

Within Neobatrachia, *Beelzebufo* differs from many Ranoidea in lacking diplasiocoely (where the last presacral is biconcave and fits against a condyle at the front of the sacrum) and from ‘derived’ ranoids (Natatanura sensu [9]) like mantellids, rhacophorines, and pyxicephalids in having moderately expanded and dorsoventrally flattened sacral diapophyses (rather than narrow cylindrical ones [10], [75]–[76], [140]). Although some ranoids lack diplasiocoely (e.g., hemisotids [75], cophyline microhylids [140]), these differ from *Beelzebufo* in cranial shape (small-mouthed) and in having widely spaced and sometimes stalked occipital condyles.

In the original description of *Beelzebufo* [26], a phylogenetic analysis using an extended version of the morphological/phenotypic data matrix of Fabrezi [92] placed *Beelzebufo* as the sister taxon of *Ceratophrys* within Ceratophryidae. With the recognition that hyperossification may lead to convergence among living taxa (see also [141]), pairs of related taxa, one ‘normal’ and one hyperossified, were included in an attempt to limit size effects. A separate analysis was also run using only the taxa with hyperossified and/or exostosed skulls (from pelobatoids, and several ranoid and hyloid lineages, including ceratophryids and bufonids); again, *Beelzebufo* always grouped with ceratophryids.

Here we have performed a detailed reanalysis of the phylogenetic placement of *Beelzebufo*, considering both an expanded taxon-sampling regime and an expanded set of character data. In addition to a morphology-only analysis, we conducted a combined evidence analysis for over 100 taxa including nucleotide data from 12 published genes. These datasets were analysed using both maximum parsimony (MP) and Bayesian inference (BI). Regardless of dataset or model choice, the Malagasy taxon *Beelzebufo* always nests within South American Ceratophryidae. Neobatrachian ingroup relationships remain poorly resolved in the BI phylogenies but in the strict consensus of the combined evidence MP trees *Beelzebufo* and extant ceratophryids consistently nest within Neobatrachia crownward of Heleophrynidae and Sooglossidae, outside Ranoidea, and well within Hyloidea (sensu [27]), equivalent to the Nobleobatrachia of [9]). Furthermore, the morphological and combined evidence analyses continue to group *Baurubatrachus* with *Beelzebufo* and extant ceratophryids. Similarly, *Telmatobius* is recovered as the sister taxon to a clade including extant ceratophryids, *Beelzebufo*, and *Baurubatrachus*, thus conforming to the membership of Ceratophryidae in Frost et al. [9], although not that of Pyron and Wiens [27] as adopted here. MP analysis recovers the Miocene *Wawelia* within Ceratophryidae as the sister taxon to the extant genus *Chacophrys*. Therefore, whether or not *Telmatobius* is excluded from Ceratophryidae (either by way of phylogeny or nomenclature), all three fossil taxa are recovered within the crown group of the clade.

In addition to those characters frequently associated with hyperossification (see above, Phylogenetic Results Summary: ch. 2.1; 7.0; 9.1; 10.1; 13.1; 38.1), *Beelzebufo* shares a more specific subset of characters with living ceratophryids, notably: interlocking premaxillary/maxillary articulation, absence of premaxillary sculpture (although it can be present as a patch in large individuals of *Ceratophrys*), absence of a palatine shelf (pars palatina) on either the premaxilla or maxilla [61], [72], [92], [102], [142], a toothed maxilla bearing unicuspid non-pedicellate teeth [9], [59], [61], [102], [117], [119], [143]–[144], an interlocking joint between the parietosquamosal shelf and crista parotica (SEE pers. obs.), and the development of dorsal dermal armour. The shape of the latter (as reconstructed, Fig. 32) most closely resembles the developing shield of a juvenile *Lepidobatrachus llanensis* figured by Fabrezi ([92]: fig.4d). These characters also differentiate *Beelzebufo* from hyperossified australobatrachians [9] such as *Calyptocephalella*, and the hyloid *Hemiphractus*. The possession of a Type III atlas [59] is another character shared between *Beelzebufo* and ceratophryids, although Trueb [60] reported the same condition in ascaphids and it is possible that a hyperossified version of Lynch's Type II morphology (cotyles separated by a small ventral gap), with extra bone deposition around the cotyles, could yield a similar appearance. Coding *Beelzebufo* as having a Type II rather than Type III atlas made no difference to its phylogenetic placement. However, the FMNH PR 2512 cranial material demonstrates that there is no embayment of the posterior skull margin, removing a potential synapomorphy with *Ceratophrys* by comparison with *Lepidobatrachus* [26].

Ruane et al. [29], using the Evans et al. [26] data matrix, also found that *Beelzebufo* grouped with ceratophryids in morphological and combined evidence analyses. The authors, however, rejected this attribution on the basis of two factors: weak support values and the fact that using *Beelzebufo* as the sole calibration point (calibrating the *Ceratophrys*-*Lepidobatrachus* split) in their BEAST analysis resulted in unrealistically old divergence estimates for crown-group Batrachia, Hyloidea, and Ranoidea. In our analyses the monophyly of Ceratophryidae also has low support values, but the monophyly of the ceratophryids considered here is low even in analyses dealing only with extant taxa [27]. Thus the placement of *Beelzebufo* among ceratophryids is robust to data and model choice, and it is not the inclusion of fossil taxa that is lowering clade support in ceratophryids. Moreover, when only three additional calibration points were added, the anomalous divergence estimates disappeared ([29]: fig. 4). Similarly, if *Beelzebufo* is used as the sole calibration point (calibrating this time the *stem* of *Ceratophrys* + *Lepidobatrachus*), the anomalous Hyloidea and Ranoidea estimates disappear and only the much deeper Batrachia node appears to be overestimated, but this time by a much smaller margin (roughly 70 Ma versus 900 Ma).

The criteria used by Ruane et al. [29] for rejecting the phylogenetic results pertaining to *Beelzebufo* are also inconsistently applied. According to them, *Beelzebufo* is not a crown ceratophryid because the divergence estimates differ from what is expected based on three other external fossil calibration points. This is clearly expressed at the beginning of the last paragraph of page 10 where the authors explicitly rule out crown-group status for *Beelzebufo* but say that they cannot rule out a stem-group position. Yet, at the end of that same paragraph, the authors conclude that it is likely that *Beelzebufo* is neither a crown nor stem-group ceratophryid because of the divergence estimates obtained when using it to calibrate the molecular clock. This statement is curious given that in the preceding paragraph the authors acknowledge the Cretaceous-aged *Baurubatrachus* as a stem ceratophryid and cite it as support for a South American origin for the clade. Including *Baurubatrachus* as a ceratophryid (whether as a stem or crown-group member) undermines the very point of Ruane et al.'s [29] argument, that the Cretaceous age of *Beelzebufo* is inconsistent with molecular divergence estimates. Unless the molecular clock is calibrated with *Beelzebufo* (either as a stem or crown-group ceratophryid), then the divergence estimates for the origin of Ceratophryidae postdates *Baurubatrachus* by roughly 30 million years.

Historically there has been some degree of variability in dating the ceratophryid lineage. Maxson and Ruibal [145] estimated that *Lepidobatrachus* had separated from the common ancestor of *Chacophrys* and *Ceratophrys* by the Eocene, with the latter taxa diverging in the early Miocene. This would be consistent with the Paleogene-Neogene record from South America, and would allow for stem ceratophryids in the Late Cretaceous. However, more recent analyses [29], [62], [146] have mostly yielded younger (Miocene) divergence estimates (∼12–20 Ma) for the *Lepidobatrachus*-*Ceratophrys* split (Table S6 in File S1), and date the stem of Ceratophryidae at ∼45–65 Ma (e.g., [62]: Table S6). These dates are reasonable given that fossil remains attributed to *Ceratophrys* have been recorded from several Late Miocene to Pleistocene localities [147]–[150], and a skull of *Lepidobatrachus* has been reported from the Pliocene of Argentina (∼5 Ma)[151]. Going beyond the living genera, *Wawelia gerholdi* [82], [152] from the Miocene of Argentina is generally accepted as a ceratophryid, or stem-ceratophryid, and additional ceratophryid material has been reported from the Oligocene and Miocene of Argentina [149]–[150].

A recent timetree for Anura by Irisarri et al. [63] recovers older divergence estimates than Ruane et al. [29], with the difference being considerable in some cases. The ranoid/hyloid split was estimated at ∼125 Ma by Ruane et al. [29] whereas Irisarri et al. [63] placed the split ∼150 Ma with 95% confidence intervals stretching as far back as 175 Ma. Likewise, Ruane et al. [29] estimated that the crown of Hyloidea ( = Nobleobatrachia) originated ∼58 Ma, whereas Irisarri et al. [63] estimated this origin as over 20 million years older, at ∼80 Ma. Interestingly, this older date for Nobleobatrachia conforms to the estimate recovered by Ruane et al. [29] when *Beelzebufo* was used as a calibration point for crown Ceratophryidae. In summary, it is our view that molecular timetrees for Anura, while converging on a consensus for the major clade divergences, still represent a work in progress and remain sensitive to internal calibration point choice (e.g., compare calibrations between [29] and [63]).

Our phylogenetic results (MP and BI of combined evidence; Figs 43–46) place *Baurubatrachus*, *Beelzebufo*, and *Wawelia* within the crown of Ceratophryidae (sensu [27]). It is also the case that the resolution among ceratophryids is poor. This is not unique to phylogenies including fossils. A recent analysis has raised questions as to the monophyly of extant *Ceratophrys* [27], and the interrelationships of *Ceratophrys*, *Lepidobatrachus*, and *Chacophrys* remain unclear ([9], [27], [29], [62] and this analysis). Most divergence estimates for Ceratophryidae are based on the *Lepidobatrachus* + *Ceratophrys* split (∼12–20 Ma depending on the analysis). We cannot rule out, and our analysis is consisent with, *Beelzebufo* as the sister taxon to a *Lepidobatrachus* + *Ceratophrys* clade. Thus it is possible that molecular divergence estimates of a young *Lepidobatrachus* + *Ceratophrys* split (∼12 Ma) are accurate. This could leave a more inclusive Ceratophryidae as having diverged in the Cretaceous and it would be this divergence that is being sampled by taxa such as *Baurubatrachus* and *Beelezbufo* and being estimated using molecular clocks when calibrated with one of these two Cretaceous taxa.

Despite the new and more complete material and the comprehensive analyses discussed above, we cannot rule out the possibility that the striking resemblance between *Beelzebufo* and extant ceratophryids is the result of convergent evolution. Like *Pyxicephalus* and *Ceratophrys* [72], *Beelzebufo* and *Ceratophrys* are both large predatory anurans living a similar lifestyle under similar environmental conditions. Anurans with strongly ossified skulls and/or exostosis have been recorded among extinct palaeobatrachids, pelobatids (e.g., *Eopelobates* [153]), pipids (e.g., *Pachybatrachus*, [115]), and gobiatines [135], and within several extant neobatrachian groups (including ceratophryids, bufonids, hylids, brachycephalids, australobatrachians, and ranoids). Moreover, in the past, large morphs may have developed within other families, as shown by *Nasikabatrachus* from India [139], [154]), which differs significantly from its small Seychellian sooglossid sister group.

Nonetheless, we have highlighted that the placement of *Beelzebufo* within Ceratophryidae is not based solely on the presence of hyperossified features (removal of these traits and reanalysis still recover the ceratophryid affinity) and, furthermore, that *Beelzebufo* possesses at least three additional morphological features that ally it with derived nobleobatrachian (hyloid) frogs to the exclusion of ranoid frogs. Indeed, of the 11 morphological traits supporting the basal-most nodes within Ranoidea, the four that are preserved in *Beelzebufo* contradict its placement within the clade (characters 3, 35, 36, and 42). The possibility of convergence is ever-present in phylogeny estimation. Therefore we encourage the construction of more character-rich morphological matrices for Anura and hope for continued fossil discoveries as these are the only path forward to further support or reject the present hypothesis of relationship.

### Biogeography

The extant fauna of Madagascar shows a high level of endemicity that reflects its long physical isolation. Indo-Madagascar began to rift from Africa in the Middle Jurassic (∼165 Ma), and from East Gondwana (Australia+Antarctica) in the Early Cretaceous (∼130 Ma), with Madagascar finally separating from the Indian subcontinent-Seychelles block in the Late Cretaceous (∼88 Ma). Similarities between the Late Cretaceous terrestrial and freshwater vertebrate faunas of India and Madagascar are therefore not unexpected, but many researchers have also noted faunal similarities with South America [28], [53], [155]–[163], and Hay et al. [28] and Case [161] posited the existence of a land route from South America to Indo-Madagascar, via Antarctica, and two land bridges (the Kerguelen Plateau and the Gunnerus Ridge), until as late as ∼80 Ma. The occurrence of a South American anuran family (Ceratophryidae) in Madagascar was considered to be consistent with this interpretation [26]. However, more recent research [31]–[33] has established that all land routes between Antarctica and Indo-Madagascar were severed and submerged by ∼115–112 Ma, well before the end of the Early Cretaceous, and that significant deep-water gaps rapidly developed as Indo-Madagascar moved northwards. This puts a much earlier time constraint on any overland dispersal between the two landmasses. Ali and Krause [33], employing a ghost lineage assessment of large terrestrial vertebrates (abelisauroid theropods, titanosaurian sauropods, and notosuchian crocodyliforms, i.e., those terrestrial vertebrates with the least probability of having swum or rafted across large marine barriers) from the Late Cretaceous of India and Madagascar, concluded that their basal stocks were likely present on the conjoined landmass before it became isolated in the Early Cretaceous. Similarly, Crottini et al. [18] have posited that oplurid iguanians and podocnemid turtles may also have been present on Madagascar in the Early Cretaceous, even earlier than previously indicated [164]. Moreover, recent discoveries in Africa have shown that many of the Late Cretaceous groups that seemed to show a South American/Indo-Madagascan distribution pattern (e.g., abelisaurid theropods [165]–[167]; notosuchian crocodyliforms [168]–[169]) were actually more widely distributed in the Early Cretaceous and earliest Late Cretaceous. Finally, although the fossil record from the Cretaceous of Antarctica is very poor (completely lacking for small-medium sized terrestrial and freshwater vertebrates), none of the dinosaurian higher taxa (i.e., titanosaurian sauropods, abelisaurid theropods, basal pygostylians, enantiornithines, ornithurines) that are represented in the Late Cretaceous of South America, Madagascar, and India have been found there. Instead, the dinosaurian fauna consists of ankylosaurs, hadrosaurs, and neornithines [33]. Thus, although the evidence is limited, the Late Cretaceous large vertebrate fauna of Indo-Madagascar appears to have been somewhat relictual, in the sense that it retained representatives of lineages whose ancestors entered Indo-Madagascar early and became isolated there [33].

**Figure 49:**
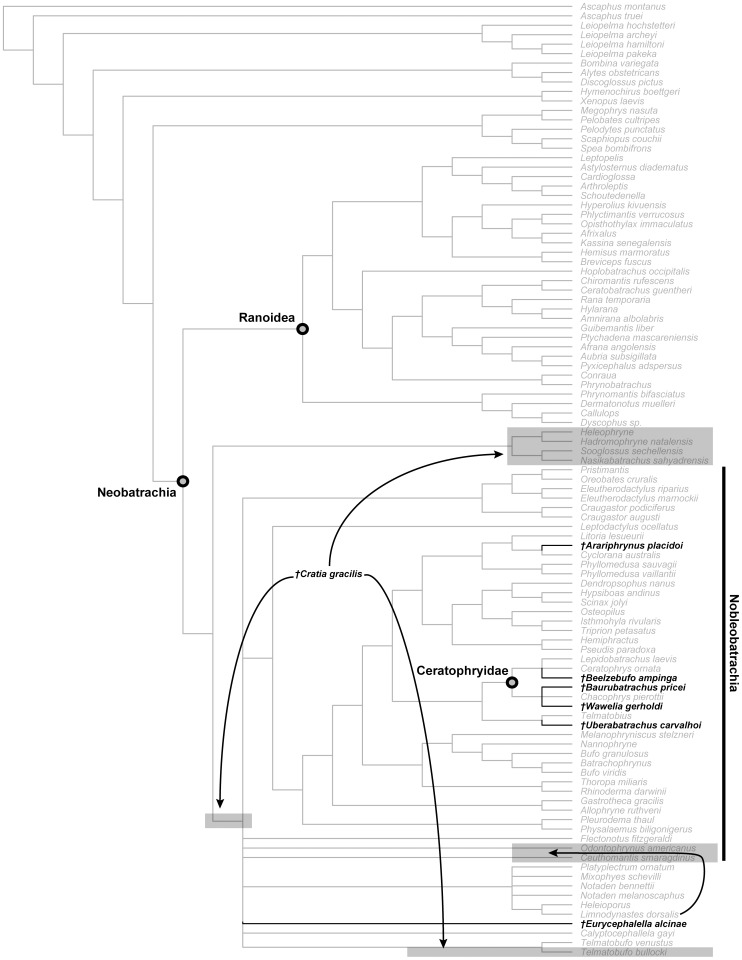
Combined evidence (maximum parsimony) tree showing placement of fossil genera discussed in text. *Arariphrynus* (Early Cretaceous); *Baurubatrachus*, *Uberabatrachus* and *Beelzebufo* (Late Cretaceous); and *Wawelia* (Miocene) all fall within Nobleobatrachia. Alternative positions (as shown) were obtained for Early Cretaceous *Cratia* and *Eurycephalella*. All except *Beelzebufo* are from South America.

Clearly this has implications for *Beelzebufo*. If *Beelzebufo* is genuinely a ceratophryid, as the results of the phylogenetic analyses continue to maintain, and its ancestors entered Indo-Madagascar by land, then it requires ceratophryids to have arisen by at least 112 Ma. This is at variance with previous molecular divergence estimates [29]–[30], [62]–[63], [146], [170], which date the radiation of Nobleobatrachia (sensu [9]) after the physical isolation of Indo-Madagascar (∼88 Ma), and the emergence of ceratophryids significantly later (Table S6 in File S1). However, Báez et al. [80] placed *Arariphrynus* and *Eurycephalella* from the Brazilian Crato Formation (∼125–112 Ma) within Nobleobatrachia, and the results of our analyses generally support this, at least for *Arariphrynus* (Fig. 49). The position of *Eurycephalella* is less stable. The Maastrichtian South American *Uberabatrachus* [83] also nests well within Nobleobatrachia (Fig. 49) as, of course, does *Baurubatrachus.* Hyloids have also been reported from the latest Cretaceous of India [171]–[172], albeit only on the basis of rare and very fragmentary material. Thus the fossil record, if correctly interpreted, offers some support for an earlier diversification of Nobleobatrachia than many molecular analyses predict. Nonetheless, on current evidence, inferring the presence of an early ceratophryid on Indo-Madagascar prior to its isolation from Antarctica remains problematic.

However, small tetrapods are not subject to the same constraints as large dinosaurs and notosuchian crocodylians in terms of overwater dispersal ability [15]–[17]. Reconstructions of elevation and drainage patterns for the Late Cretaceous of Africa [173] indicate that many large rivers flowed out onto its eastern coastline. As today, these would have carried mats of vegetation into the proto-Indian Ocean and/or Mozambique Channel. Palaeo-oceanographic modelling [20], [33], [174] suggests that from at least the Early Palaeocene (65 Ma) until the Miocene, west to east ocean currents could have transported rafts of vegetation across to Madagascar. Although paleocurrent direction has not been modelled for the Maastrichtian, it is unlikely to have been significantly different, given that the relative positions of Africa and Madagascar remained unchanged [31], and Madagascar still lay too far south to be affected by equatorial currents [175]. Indeed, this route into Madagascar has been proposed by other researchers [32].

Although anurans are often considered poor candidates for trans-oceanic dispersal because of their perceived intolerance of salt water [10], molecular phylogenetic analyses of the Malagasy anuran fauna [3], [5], [13]–[16], [18], [176]–[177] have provided strong evidence that the ancestors of at least some extant Malagasy taxa (e.g., hyperoliids in the late Oligocene/early Miocene [15]–[16]; *Ptychadena* in the Plio-Pleistocene [21]) arrived from Africa by sea. Mantellids (and perhaps dyscophine microhylids) may also have dispersed back overwater to Madagascar from India during the latest Cretaceous or Early Paleocene [20], [178]–[179], albeit from an ancestral Indo-Madagascan stock [15]–[16], [176], [178]. This is relevant to *Beelzebufo* because a thick-skinned arid-adapted anuran would be a good candidate for dispersal of this kind. Nonetheless, this solution would still require that ceratophryids had evolved by the Maastrichtian and that they were already present in eastern Africa by that time (or on another Gondwanan landmass with favorable current flow).

Ranoids dominate the modern African anuran fauna, but pipoids dominate the Cretaceous fossil record of both South America and Africa [80], [115], [180]–[190], probably because they are more aquatic and thus more likely to be preserved. Almost nothing is currently known of the non-pipoid Cretaceous anurans of Africa south of the Sahara, other than possible ranoid fragments from the Cretaceous of Sudan [191]. Fragmentary anuran material has been reported from the Early Cretaceous of Cameroon [192] and Malawi [193], but it has not yet been described. The Malawian material includes a strongly ossified frontoparietal (figured in [193]) but, as there were also hyperossified pipoids in Africa (*Pachybatrachus* [115]), it cannot be attributed without more detailed comparisons.

Today, the anuran fauna of Madagascar is exclusively ranoid and divergence estimates suggest that early microhylids [18], [29], [62] and the ancestral stock of mantellines-rhacophorines [75], [176], [194]–[195] had reached Indo-Madagascar by at least the Late Cretaceous. However, taking into account modern and fossil distributions on neighbouring Gondwanan landmasses [186] and estimated molecular divergence dates for living frogs [18], [29], [62], [170], [196], representatives of several other anuran lineages could also have been present on Indo-Madagascar in the Cretaceous prior to its break up, notably the ancestral stock of Seychellian sooglossids and the Indian *Nasikabatrachus* [139], and the ancestors of one or more of the ranoid lineages now endemic to India (e.g., micrixalids, nyctibatrachids, lankanectids, dicroglossids, ranixalines [62], [75], [176], [195]. Potentially, as with the dinosaurs and crocodyliforms, the Late Cretaceous anuran fauna could also have contained representatives of other lineages that had diversified early enough to enter Indo-Madagascar prior to its isolation, notably pipoids, ‘basal’ neobatrachian groups like heleophrynids, and australobatrachians [59], [80], [171], [197]–[201]. Australobatrachians (sensu [9]) include the Australasian myobatrachians and the South American *Calyptocephalella* and are estimated to have arisen ∼150–70 Ma [29], [62], [139], [170], [200], a date supported by records of *Calyptocephalella*, or a near relative, from the Late Cretaceous of Argentina [141], [201]. The earliest Australian anuran record places a myobatrachid (*Lechriodus*) there in the Eocene (∼54.5 Ma [198]), but *Indobatrachus* from the Palaeocene/Lower Eocene of India has sometimes been attributed to this clade [59], [197], [199], a classification that, if correct, would imply the group was also present on Indo-Madagascar in the Cretaceous. *Beelzebufo* shows no particular resemblance to members of any of these groups, nor does it group with them in any phylogenetic analysis.

The rich fossil record from the Maevarano Formation has demonstrated that the latest Cretaceous fauna of Madagascar (dominated by non-avialan dinosaurs, archaic birds, crocodyliforms, and mammals ([34]: table 1; [163]) was strikingly different from that of today and it is likely that this change was precipitated by events at the end of the Cretaceous [20]. The fate of the smaller tetrapods is more difficult to predict. Molecular studies suggest that endemic Malagasy anurans like the mantellids and microhylids underwent a rapid diversification around this time [3], [13]–[16], [176], [195], [202], possibly in response to rainforest expansion [18]. The fate of the archaic frog fauna, including *Beelzebufo*, remains unknown pending the discovery of Palaeogene localities.

## Conclusions

New material of *Beelzebufo*, including a partial association, has permitted a more detailed description of its anatomy, revealing a large-headed, heavily armoured anuran that was almost certainly an ambush predator of small vertebrates. New phylogenetic analyses, using both morphological and combined data sets, continue to place *Beelzebufo* within hyloid anurans, in the family Ceratophryidae. We recognise that this is problematic in relation to many recent molecular divergence estimates and palaeobiogeography, and that it will, doubtless, raise further discussion. With respect to palaeobiogeography, however, it is important to acknowledge how little is known of the Mesozoic small vertebrates of Africa, India, Antarctica, or Australia. Given that animals as large as abelisaurid and noasaurid dinosaurs were not recorded from Africa until 2004 [165], it is perhaps premature to rule out the possibility that one or more groups of hyloid frogs might have been present there as well. Continued work on the Mesozoic and Tertiary small vertebrates of all Gondwanan landmasses is crucial if we are to unravel their complex history, and *Beelzebufo* suggests there are more surprises in store.

## Supporting Information

File S1 **Documentation, sensitivity analyses, supplementary trees and tables.** Section A. Three-dimensional digital skeletal model of *Beelzebufo ampinga.* Section B. List of taxa used in phylogenetic analyses. Section C. Morphological character list used in phylogenetic analyses. Section D. Sensitivity analyses run. Section E. Osteological specimens examined. Section F. References cited in Sections A–E. Figure S1. Sensitivity analysis 1: morphological dataset. No character 6, no *Thaumastosaurus*, no *Cratia*, no hyperossified characters. Figure S2. Sensitivity analysis 2: morphological dataset. No character 6, no *Thaumastosaurus*, no *Cratia*, only hyperossified characters. Figure S3. Sensitivity analysis 3: morphological dataset. No character 6, no *Thaumastosaurus*, no *Cratia*, no *Wawelia*. Figure S4. Sensitivity analysis 4: morphological dataset. No character 6, no *Thaumastosaurus*, no *Cratia*, no *Baurubatrachus*. Figure S5. Sensitivity analysis 5: morphological dataset. No character 6, no *Thaumastosaurus*, no *Cratia*, no *Wawelia*, no *Baurubatrachus*. Figure S6. Sensitivity analysis 6: combined dataset. No character 6, no *Thaumastosaurus*, no *Cratia*, no hyperossified characters. Figure S7. Sensitivity analysis 7: combined dataset. No character 6, no *Thaumastosaurus*, no *Cratia*, no *Baurubatrachus*. Figure S8. Sensitivity analysis 8: combined dataset. No character 6, no *Thaumastosaurus*, no *Cratia*, no *Wawelia*. Figure S9. Sensitivity analysis 9: combined dataset. No character 6, no *Thaumastosaurus*, no *Cratia*, no *Wawelia*, no *Baurubatrachus*. Table S1. Changes in specimen numbers and identifications from Evans et al. (2008). Table S2. *Beelzebufo* specimens used in skeleton and skull reconstructions. Table S3. Axial column element lengths of representative anuran taxa. Table S4. Measurements and proportions of tibiofibula and tibiale-fibulare of representative anuran taxa. Table S5. Skull measurements and proportions of representative anuran taxa. Table S6. Molecular divergence dates for Hyloidea
(PDF)

Video S1 **Supplemental video to** **Fig. 1** **.** Three-dimensional model of *Beelzebufo ampinga* skeleton through a full rotation around the midline axis, highlighting sources of model materials. Model as imaged and described in Fig. 1, except with left forelimb visible, and reconstructed jaws and forelimbs rendered transparent to display ventral cranium clearly.
(MPG)

Video S2 **Supplemental video to** **Fig. 4a** **.** Skull reconstruction of *Beelzebufo ampinga*. Model as imaged and described in Fig. 4a, but through a full rotation around the midline axis.
(MPG)

Video S3 **Supplemental video to** **Fig. 5** **.** Three-dimensional model of *Beelzebufo ampinga* skeleton through a full rotation around the midline axis, highlighting specimen FMNH PR 2512 (dark blue) from other specimens of *Beelzebufo* (light blue), and showing sources of reconstructed materials. Model as imaged and described in Fig. 5, except with left forelimb visible, and reconstructed jaws and forelimbs rendered transparent to display ventral cranium clearly.
(MPG)
