# Supplementary material for: New Material of Beelzebufo, a Hyperossified Frog (Amphibia: Anura) from the Late Cretaceous of Madagascar
Source: PLoS One. 2014 Jan 28;9(1):e87236. doi: 10.1371/journal.pone.0087236 (PMC3905036; doi:10.1371/journal.pone.0087236)
Supplement: File S1 — Documentation, sensitivity analyses, supplementary trees and tables. Section A. Three-dimensional digital skeletal model of Beelzebufo ampinga. Section B. List of taxa used in phylogenetic analyses. Section C. Morphological character list used in phylogenetic analyses. Section D. Sensitivity analyses run. Section E. Osteological specimens examined. Section F. References cited in Sections A–E. Figure S1. Sensitivity analysis 1: morphological dataset. No character 6, no Thaumastosaurus, no Cratia, no hyperossified characters. Figure S2. Sensitivity analysis 2: morphological dataset. No character 6, no Thaumastosaurus, no Cratia, only hyperossified characters. Figure S3. Sensitivity analysis 3: morphological dataset. No character 6, no Thaumastosaurus, no Cratia, no Wawelia. Figure S4. Sensitivity analysis 4: morphological dataset. No character 6, no Thaumastosaurus, no Cratia, no Baurubatrachus. Figure S5. Sensitivity analysis 5: morphological dataset. No character 6, no Thaumastosaurus, no Cratia, no Wawelia, no Baurubatrachus. Figure S6. Sensitivity analysis 6: combined dataset. No character 6, no Thaumastosaurus, no Cratia, no hyperossified characters. Figure S7. Sensitivity analysis 7: combined dataset. No character 6, no Thaumastosaurus, no Cratia, no Baurubatrachus. Figure S8. Sensitivity analysis 8: combined dataset. No character 6, no Thaumastosaurus, no Cratia, no Wawelia. Figure S9. Sensitivity analysis 9: combined dataset. No character 6, no Thaumastosaurus, no Cratia, no Wawelia, no Baurubatrachus. Table S1. Changes in specimen numbers and identifications from Evans et al. (2008). Table S2. Beelzebufo specimens used in skeleton and skull reconstructions. Table S3. Axial column element lengths of representative anuran taxa. Table S4. Measurements and proportions of tibiofibula and tibiale-fibulare of representative anuran taxa. Table S5. Skull measurements and proportions of representative anuran taxa. Table S6. Molecular divergence dates for Hyloidea (PDF) [file pone.0087236.s001.pdf]

File S1. Documentation, sensitivity analyses, supplementary trees and tables.

Section A. Three-dimensional digital skeletal model of *Beelzebufo ampinga*.

Description and Rationale

The three-dimensional digital skeletal model of *Beelzebufo ampinga* uses only elements from *Beelzebufo* and from the postcranial skeleton of a single individual of *Ceratophrys aurita* (LACM 163430). The goal of the reconstruction was to create a conservative, repeatable, and defensible model that relied on a minimum of assumptions for and alterations to the model animal skeleton LACM 163430 beyond an initial calibration and, aside from mirroring and filling in missing spaces in the tooth row, a minimum of alteration to specimens of *Beelzebufo*. No non-*Beelzebufo* material was used in the cranial model as it was originally constructed, but for aesthetic reasons a re-scaled and re-proportioned surface of the jaws of *Ceratophrys aurita* (LACM 163430) was added prior to imaging for the manuscript for Figures 1, 2, and 5 (see below). For alterations that were necessary, we adhered whenever possible to an assumption of isometric shape change, not for biological reasons but rather to keep any shape change as conservative and non-arbitrary as possible. All size and shape changes were documented and comparisons with original, non-altered materials as simple as a side-by-side file comparison. All shape positions exist in a single coordinate space and can be easily recorded, and differences in hypothetical positions tracked exactly.

The relative completeness of FMNH PR 2512, as well as it being the only recovered specimen with both crania and postcrania, made it the best candidate for an accurate composite skeletal reconstruction. Because of a lack of knowledge of relative proportions amongst those specimens in the cranial and postcranial skeleton of *Beelzebufo* not from FMNH PR 2512, we sought to minimize arbitrary, non-mirroring alterations (isometrically or allometrically) of digital surfaces of elements, and allowed it in only one case for *Beelzebufo* (we increased the size of UA 9947 isometrically by a factor of 1.06305). This resulted in the exclusion of some specimens, notably vertebrae from the posterior portion of the column, from the reconstruction; this exclusion implies only that, within our scaling of LACM 163430 as calibrated to FMNH PR 2512 (see below), these vertebrae are outside the size range of a hypothetical individual of *Beelzebufo* having the size of FMNH PR 2512.

Cranial Model (Figs 1−5)

Work on the digital cranial model of *Beelzebufo* began early in 2010 but was catalyzed by discoveries of additional material of FMNH PR 2512 in the austral winters of 2010 and 2011. Because FMNH PR 2512 is preserved from its cranial posterolateral margin to its midline, it allowed for a clear and well-defined posterior skull width and height, within the context of which it became possible to place additional specimens with a high degree of confidence, although the precise relationship of the anterior and posterior portions of the skull is not yet known because of a lack of overlap in morphology. Final placement of cranial fragments was ultimately based on assumptions of the extent and form of missing material, largely through extrapolation of sutural surfaces, and largely on the anterior portions of the skull.

The scale or proportion of no specimen used in the cranial reconstruction was altered. Although it is not the case that all specimens are from individuals of a single size, there is no direct evidence available to define or restrict relative changes in a way not considered arbitrary. No non-*Beelzebufo* material was used in the cranial model as it was originally constructed, but a re-scaled and re-proportioned surface of the jaws of LACM 163430 (mediolateral and dorsoventral dimensions increased by a factor of 1.16545; anteroposterior dimension increased by a factor of 1.38148) was added prior to imaging for the manuscript (Figs 1–2). Most missing but reconstructed morphology was inferred through the assumption of bilateral symmetry;

mirror-imaging of relevant specimen surfaces were used to accomplish this. Homologous portions of overlapping morphology, particularly in the maxilla and quadratojugal, allowed for reasonable composite modelling from multiple specimens although, in the facial process of the maxilla in particular, slight differences in size between FMNH PR 2510 and FMNH PR 2507 resulted in a small portion (~2 mm) of doubled morphology in the anteromedial portion of the process.

Gaps in the tooth row in the premaxilla, at the premaxilla/maxilla boundary, and within the maxilla were reconstructed not by mirroring. but by cloning portions of the tooth row of FMNH PR 2510. This was the only instance of deliberately introducing incorrectly-positioned morphology to the model, but is justifiable given the relative invariance of tooth row morphology in anurans.

Postcranial model (Figs 1, 2, 5, and 32)

Unlike the skull, the paucity of preserved postcranial remains of *Beelzebufo* necessitated the introduction of a model framework of a known skeleton. This missing material was modelled using a large female skeleton of *Ceratophrys aurita* (LACM 163430). *Ceratophrys* was selected as a model animal due to the consistent placement of *Beelzebufo* within the Ceratophryidae in phylogenetic analyses, as well as its similarly large size and inferred similar ecological habit.

Because the atlas/occipital articulation from FMNH PR 2512 is preserved, we use this relationship to calibrate the entire postcranium of LACM 163430 to the atlas and second presacral (PS) vertebra, and thus to the skull of FMNH PR 2512. As a first step, however, both the atlas + PS 2 of LACM 163430 and the atlas of FMNH PR 2512 were compared to the holotype (UA 9600) cervical + PS 2 as a keystone surface, because of the relative completeness of the holotype and our desire to use the holotype as part of the reconstruction.

The atlas + PS 2 of LACM 163430 was largely size and shape consistent with UA 9600 in the anteroposterior and mediolateral dimensions when increased by a factor of 1.10124. The dorsoventral dimension, however, was still relatively low when compared to UA 9600, and so the effective dorsoventral dimension was increased by a factor of 1.23773. These numbers were used to create a transformed axial column polygon mesh of LACM 163430, resulting in a surface representing the model size of the axial column (and by assumption, appendicular skeleton) of the holotype of *Beelzebufo*.

In scaling the holotype to FMNH PR 2512, an isometrically applied factor of 0.955296 was required to create an atlas + PS 2 the size of that of FMNH PR 2512. This effectively smaller holotype surface is what was incorporated into the model.

Finally, to scale the holotype-calibrated axial column (and by assumption appendicular material) of LACM 163430 to the skull of FMNH PR 2512, we used the factors of 1.10124 and 0.955296 to arrive at a model factor for LACM 163430 of 1.05201. This factor was applied uniformly to the remaining postcranial surfaces of LACM 163430, and this served as the framework into which elements of *Beelzebufo* were placed. With the exception of presacral vertebra UA 9947, which was expanded isometrically by a factor of 1.06305, no further alterations to the size of any element in the model was altered after this point.

Likely owing to a number of factors including phylogenetic position, size, convergence, and the conservative nature of the anuran skeleton, the model skeleton provided a surprisingly good fit for most (though not all) postcranial materials of *Beelzebufo*. In instances where combining homologous portions of the skeleton yielded obvious discontinuities (such as the relatively posteriorly projecting transverse processes of FMNH PR 2512 Vertebra B versus the *Ceratophrys*-based PS5, and the much more dorsally accentuated dorsal ridge on the urostyle of *Ceratophrys*), triangles in the *Ceratophrys* model were always blended to accommodate those of *Beelzebufo*.

Unlike the cranial model, use of mirroring was restricted because the model skeleton provides a complete view of the reconstructed size and elements of the animal. Mirroring was used in FMNH PR 2512 Vertebra B and sacral vertebra FMNH PR 2003 to provide midline symmetry to the axial column.

Videos

Supplemental mpeg videos for Figs 1, 4a, and 5 were created using the Avizo 7.1 Movie Maker module. Six hundred frames were captured at a frame rate of 50/second and a quality metric of 0.4. The videos associated with Figs 1 and 5 were captured with a screen resolution of 528 x 1008, and the surface models of the forelimbs, pectoral girdle, and jaws were rendered transparent (0.8) to improve visibility of highlighted specimens on the ventral aspect of the skull and axial column. The video for Fig. 4 was captured at a screen resolution of 992 x 1008.

Associated videos

Video S1 Supplemental video to Fig. 1. Three-dimensional model of *Beelzebufo ampinga* skeleton through a full rotation around the midline axis, highlighting sources of model materials. Model as imaged and described in Fig. 1, except with left forelimb visible and reconstructed jaws and forelimbs rendered transparent to display ventral cranium clearly.

Video S2 Supplemental video to Fig. 4a. Skull reconstruction of *Beelzebufo ampinga*. Model as imaged and described in Fig. 4a, but through a full rotation around the midline axis.

Video S3 Supplemental video to Fig. 5. Three-dimensional model of skeleton of *Beelzebufo ampinga* through a full rotation around the midline axis, highlighting specimen FMNH PR 2512 (dark blue) from other specimens of *Beelzebufo* (light blue), and showing sources of reconstructed materials. Model as imaged and described in Fig. 5, except with left forelimb visible and reconstructed jaws and forelimbs rendered transparent to display ventral cranium clearly.

Section B. Reference taxon list for species used in the combined phylogenetic analysis.

The revised nomenclatural revisions (in bold) are based on Amphibian Species of the World 5.6, an Online reference [Section F, Reference 1]

Afrana angolensis = Amieta angoliensis
Afrixalus
    MORPH: fulvovittatus
    DNA: knysnae
Allophryne ruthveni
Alytes obstetricans
Amnirana albolabris ***= Hylarana albolabris***
†*Arariphrynus placidoi*
Arthroleptis
    MORPH: *adolphifriederici*
    DNA: *xenodactyloides*
Ascaphus montanus
Ascaphus truei
Astylosternus diadematus
Aubria subsigillata
Batrachophrynus sp.
†*Baurubatrachus pricei*
†*Beelzebufo ampinga*
Bombina variegata
Bufo granulosus ***= Incilius nebulifer***

Bufo viridis
Callulops
MORPH: *kopsteini*
DNA: *robustus*
Calyptocephallela gayi
Cardioglossa
MORPH: *cyaneospila*
DNA: *elegans*
Ceratobatrachus guentheri
Ceratophrys ornata
Chacophrys pierottii
Chiromantis rufescens
Conraua
MORPH: *crassipes*
DNA: *robusta*
†*Cratia gracilis*
Cyclorana australis **= *Litoria australis***
Dendropsophus nanus
Dermatonotus muelleri
Discoglossus pictus
Dyscophus
MORPH: sp.
DNA: *insularis*
†*Eurycephalella alcinae*
Flectonotus fitzgeraldi
Gastrotheca gracilis
Guibemantis liber
Heleioporus
MORPH: *eyeri*
DNA: *australiacus*
Heleophryne
MORPH: *natalensis* **= *Hadromophryne natalensis***
DNA: *purcelli*
Hemiphractus
MORPH: sp
DNA: *scutatus*
Hoplobatrachus occipitalis
Hylarana
MORPH: *albolabris*
DNA: *nicobariensis*
Hymenochirus boettgeri
Hyperolius kivuensis
Hypsiboas andinus **= *Hypsiboas riojanus***
Isthmohyla rivularis
Kassina senegalensis
Leiopelma archeyi
Leiopelma hamiltoni
Leiopelma hochstetteri
Leiopelma pakeka
Lepidobatrachus laevis
Leptodactylus ocellatus **= *Leptodactylus bolivianus***
Leptopelis
MORPH: *christyi*

DNA: *argenteus*
Limnodynastes dorsalis
Litoria lesueurii
Megophrys nasuta
Melanophryniscus stelzneri
Mixophyes schevilli
Nannophryne variegatus **= *Bufo variegatus***
Odontophrynus americanus
Opisthothylax immaculatus
Osteopilus
MORPH: sp
DNA: *vastus*
Pelobates cultripes
Pelodytes punctatus
Phlyctimantis verrucosus
Phrynobatrachus
MORPH: *acutirostris*
Phrynomantis bifasciatus
Phyllomedusa sauvagii
Phyllomedusa vaillantii
Physalaemus biligonigerus
Platyplectrum ornatum
Pleurodema thaul
Pristimantis
MORPH: *huicundo*
DNA: *ashkapara* **= *Yunganastes ashkapara***
Pseudis paradoxa
Ptychadena mascareniensis
Pyxicephalus adspersus
Rana temporaria
Rhinoderma darwinii
Scaphiopus couchii
Schoutedenella **= *Arthroleptis***
MORPH: *sylvaticus, lameerei, schubotzi*
DNA: *schubotzi*
Scinax jolyi
Sooglossus sechellensis
Spea bombifrons
Telmatobius
MORPH: *scrocchii*
DNA: *niger*
Telmatobufo venustus
†*Thaumastosaurus sulcatus*
Thoropa miliaris
Triprion petasatus
†*Uberabatrachus carvalhoi*
†*Wawelia gerholdi*
Xenopus laevis

Section C. Phenotypic character set used in both the Maximum Parsimony and Bayesian Inference analyses.

Characters are derived from Fabrezi [Section F, reference 2], which were subsequently modified by Evans et al. [Section F, reference 3] and Báez et al. [Section F, reference 4]. Characters potentially related to hyperossification are denoted here with the abbreviation "**H**". Character 6 is particularly problematic to interpret and score, especially in hyperossified taxa and was excluded in the phylogenetic analyses.

1. Nasals, medial contact: present (0) or absent (1). **H**
2. Skull, cranial exostosis: absent (0) or present (1). **H**
3. Skull, shape of skull roof in orbital region (i.e., frontoparietal): parallel-sided (0), wider anteriorly (1), or wider posteriorly (2). **H**
4. Skull, minimum width of skull roof in orbital region relative to orbital width: less than 1/4 (0), between 1/4 and 1/3 (1), or more than 1/3 (2). **H**
5. Frontoparietal, nature of midline contact: no medial contact (0), sutured or partially fused (1), or fused along entire length (2). **H**
6. Frontoparietal fenestra, sphenethmoid contribution: completely surrounded anteriorly by sphenethmoid (0) or not completely surrounded anteriorly by sphenethmoid (1).
7. Sphenethmoid, dorsal exposure: absent (0) or present (1). **H**
8. Sphenethmoid, ventral configuration: single (0) or divided (1).
9. Parieto-squamosal arch: absent (0) or present (1). **H**
10. Squamosal, development of otic ramus: absent or rudimentary (0), overlapping crista parotica (1), or overlapping crista parotica and otoccipital (2). **H**
11. Squamosal, development of zygomatic ramus: reduced (0), moderately developed ending free (1), or well developed, articulated with maxilla (2). **H**
12. Premaxillary and maxillary teeth: absent (0) or present (1).
13. Tooth, cusp shape: bicuspid (0) or monocuspid (1). **H**
14. Quadratojugal: absent (0) or present (1).
15. Contact of quadratojugal and maxilla: absent (0) or present (1).
16. Premaxilla, palatine shelf: present (0) or absent (1).
17. Premaxilla, lingual process: absent (0) or present (1).
18. Vomer, teeth: present (0) or absent (1).
19. Vomer, anterior process: reaching maxillary arch (0) or not reaching maxillary arch (1).

20: Palatine as a separate element (neopalatine of Trueb 1993): absent (0) or present (1).

21. Pterygoid, length of anterior ramus: long, reaching antorbital planum (0) or short (1).
22. Pterygoid and parasphenoid, contact: absent (0) or present (1). **H**
23. Parasphenoid, shape of anterior end of cultriform process: serrated or rounded (0), acuminate (1), or notched (2).
24. Parasphenoid, anterior extent of cultriform process: well posterior to level of antorbital plane (0), at level of antorbital plane or slightly posteriorly (1), or anterior to level of antorbital plane (2).
25. Odontoids on lower jaw: absent (0) or present (1). **H**
26. Hyalia, general configuration: complete (0), distally incomplete (1), or proximally incomplete (2).
27. Hyale, anterior processes: absent (0) or present (1).
28. Hyoid plate, anterolateral process: absent (0) or present (1).
29. Hyoid plate, shape of anterolateral process: pointed (0), dilated distally (1), or expanded (2).
30. Hyoid plate, posterolateral process: absent (0) or present (1).
31. Hyoid ossification, posteromedial process: present on cartilaginous stalk (0), abuts directly on hyoid (1), or invades hyoid (2).
32. Parahyoid: absent (0) or present (1).

33. Hyoid plate, calcifications: absent (0) or present (1).
34. Presacral vertebrae, number: eight (0), seven (1), or seven (with 8th fused to sacrum) (2).
35. Atlas vertebrae, cotyle arrangement: fully confluent (Lynch type 3) (0), narrowly separated but distinct (Lynch's type 2) (1), or widely separated (Lynch's type 1) (2).
36. Presacral vertebrae, posterior-most centrum morphology: opisthocoelous (0), procoelous (1), or biconcave (2).
37. Presacral vertebrae, imbrication of posterior-most four neural arches: imbricate (0) or non-imbricate (1).
38. Presacral vertebrae, height of neural spines in anterior presacrals: low (0) or high (1). **H**
39. Presacral vertebrae, orientation of transverse processes of 6th presacral: nearly perpendicular to axial axis (0), moderately forward (1), markedly forward (2), or posteriorly inclined (3).
40. Presacral vertebrae, length of transverse processes of posterior presacrals relative to sacral diapophyses: clearly shorter (0) or equal to subequal (1).
41. Presacral vertebrae, uncinate processes on transverse process of third vertebra: present (0) or absent (1).
42. Sacral vertebrae, expansion of diapophyses: widely expanded (length of distal end of sacral rib equal or greater than total width of sacrum) (0), moderately dilated (anterior and posterior margins of sacral rib clearly divergent, distal end of rib flared by comparison with proximal end, but less than total sacral width) (1), or cylindrical (anterior and posterior margins of sacral rib more or less parallel, distal end narrow and similar in diameter to proximal end) (2).

    This is modified from [S4] which had: "(0) widely expanded (ratio of length of distal end to mediolateral width greater than 1.5); (1) moderately expanded (ratio of distal end to mediolateral width greater than 0.75 but less than 1.5); (2) unexpanded or 'narrow' (ratio of length of distal end to mediolateral width less than 0.75)." This wording is problematic, as the authors presumably mean the total width across the sacrum. However, we measured specimens of three of the taxa that are scored as "0" (i.e. length/width greater than 1.5); they all fell in the "1" category (1.1−1.3, 1.2, 1.1).
43. Sacrum and urostyle, nature of articulation: bicondylar (0), monocondylar (1), or fused (2).
44. Urostyle, transverse processes: present (0) or absent (1).
45. Urostyle, length relative to presacral column: nearly as long (0), longer (1), or much shorter (2).
46. Free ribs: absent (0) or present (1).
47. Free intervertebral discs in subadults: absent (0) or present (1).
48. Dorsal shield: absent (0) or present (1).
49. Omosternum: absent (0) or present (1).
50. Omosternum, degree of ossification: completely cartilaginous (0) or with ossified style (1).
51. Omosternum, shape of ossified style: proximally forked (0) or proximally unforked (1).
52. Sternum, degree of ossification: completely cartilaginous (0) or with ossified style (1).
53. Sternum, expansion of coracoid relative to its length: less than one half (0) or more than one half (1).
54. Shape of clavicle: curved (0) or straight (1).
55. Clavicle orientation: directed anteriorly (0) or perpendicular to sagittal plane (1).
56. Epicoracoid cartilages in coracoideal region: overlapping (0) or not overlapping (usually extremely reduced) (1).
57. Scapula, maximum diameter of glenoid fossa relative to scapular shaft: more than one (0), between one and one half (1), or less than one half (2).
58. Scapula, anterior lamina: absent (0) or present (1).
59. Clavicle-scapular relation: overlapping anteriorly (0), abutting medially (1), or fused (2).
60. Intercalary elements: absent (0) or present (1).
61. Postaxial carpals (ulnare and distals 5 and 4): all free (0), ulnare free, 5+4 fused (1), or ulnare free, 3+4+5 fused (2).

62. Prepollex: one spherical proximal element (0), two elements, the distal one elongated (1), three or more elements (2), or two elements, the distal one hypermorphic (3).
63. Finger 4, shape of terminal phalanx: straight (0) or curved (1).
64. Finger 4, distal tip of terminal phalanx: simple (slightly knobbed or rounded) (0), notched (1), T-shaped (2), Y - shaped (3), or clearly knobbed (4).
65. Carpal torsion: absent (0) or present (1).
66. Ilium, well-developed dorsal crest on iliac shaft: absent (0) or present (1).
67. Epipubis: absent (0) or present (1).
68. Femoral crest: absent (0) or present (1).
69. Os sesamoides tarsale: absent (0) or present (1).
70. Distal tarsals 3 and 2: free (0) or fused (1).
71. Discrete distal tarsal 1: absent (0) or present (1).
72. Prehallux: one spherical proximal element (0), two elements, the distal one enlongate (1), three or more elements (2), or two elements, the distal one hypermorphic and spade-like (3).
73. Shape of terminal phalanx of toe: straight (0) or curved (1).
74. Distal tip of terminal phalanx of toe: simple (slightly knobbed or rounded) (0), notched (1), T-shaped (2), Y-shaped (3), or clearly knobbed (4).
75. Pupil shape: vertical (0), triangular (1), or horizontal (2).

Section D. Sensitivity Analyses run.

Files for each sensitivity analysis are available in the Documents section of project page on MorphoBank (www.morphobank.org/permalink/954. For associated trees see pp.S11–19.

[BB1.tnt]
All characters, all taxa except *Uberabatrachus*:
An initial MP analysis used the full matrix and yielded 63,108 most parsimonious trees.

[bb4.tnt.beelzebufo.morphology.nohypeross.tnt]
Sensitivity analysis 1: morphological dataset. No character 6, no *Thaumastosaurus*, no *Cratia*, no hyperossified characters:
This is the primary morphology-only analysis. Characters associated with hyperossification have been excluded. Results: MPTs=2,648, L=; CI=0.279, RI=0.410. **Fig. S1.**

[bb4.tnt.beelzebufo.morphology.onlyhypeross.tnt]
Sensitivity analysis 2: morphological dataset. No character 6, no *Thaumastosaurus*, no *Cratia*, only hyperossified characters:
This is the primary morphology-only analysis. Only characters associated with hyperossification were used, all other characters were excluded. Results: MPTs=99,999, L=101; CI=0.279, RI=0.410. **Fig. S2.**

[bb4.tnt.beelzebufo.morphology.noWawelia.tnt]
Sensitivity analysis 3: morphological dataset. No character 6, no *Thaumastosaurus*, no *Cratia*, no *Wawelia*:
This is the primary morphology-only analysis with *Wawelia* excluded. Results: MPTs=6,376, L=691; CI=0.152, RI=0.556. **Fig. S3.**

[bb4.tnt.beelzebufo.morphology.noBaurubatrachus.tnt]
Sensitivity analysis 4: morphological dataset. No character 6, no *Thaumastosaurus*, no *Cratia*, no *Baurubatrachus*:

This is the primary morphology-only analysis with *Baurubatrachus* excluded. Results: MPTs=6,376, L=691; CI=0.152, RI=0.556. **Fig. S4.**

[bb4.tnt.beelzebufo.morphology.noWawelia.noBaurubatrachus.tnt]
Sensitivity analysis 5: morphological dataset. No character 6, no *Thaumastosaurus*, no *Cratia*, no *Wawelia*, no *Baurubatrachus*:

This is the primary morphology-only analysis with *Wawelia* and *Baurubatrachus* excluded. Results: MPTs=6,376, L=691; CI=0.152, RI=0.556. **Fig. S5.**

[beelzebufo.combined.nohyperossifiedcharacters.tnt]
Sensitivity analysis 6: combined dataset. No character 6, no *Thaumastosaurus*, no *Cratia*, no hyperossified characters:

This is the primary combined data analysis. Characters associated with hyperossification have been excluded. Results: MPTs=2,670, L=35,800; CI=0.279, RI=0.410. **Fig. S6.**

[Beelzebufo.combined.noBaurubatrachus.tnt]
Sensitivity analysis 7: combined dataset. No character 6, no *Thaumastosaurus*, no *Cratia*, no *Baurubatrachus*:

This is the primary combined data analysis with *Baurubatrachus* excluded. Results: MPTs=24, L=36,014; CI=0.279, RI=0.410. **Fig. S7.**

[Beelzebufo.combined.noWawelia.tnt]
Sensitivity analysis 8: combined dataset. No character 6, no *Thaumastosaurus*, no *Cratia*, no *Wawelia*:

This is the primary combined data analysis with *Wawelia* excluded. Results: MPTs=4, L=36014; CI=0.279, RI=0.410. **Fig. S8**.

[Beelzebufo.combined.noWawelia_noBaurubatrchus.tnt]
Sensitivity analysis 9: combined dataset. No character 6, no *Thaumastosaurus*, no *Cratia*, no *Wawelia*, no *Baurubatrachus*:

This is the primary combined data analysis with *Wawelia* and *Baurubatrachus* excluded. Results: MPTs=4, L=36,013; CI=0.279, RI=0.410. **Fig. S9.**

Section E. Osteological specimens examined for morphological analysis and comparison.

Male (M) or female (F) recorded where data available.

Natural History Museum, London (names in brackets represent old names on specimen labels/catalogue; current names taken from Frost [Section F, reference 1]).

Pelobatoidea: **Pelobatidae**: *Pelobates cultripes* 1920.1.20.682; *Pelobates cultripes* unnumbered; *Pelobates fuscus* 1920.1.20.680; *Pelobates fuscus* 97.5.8.15 (F); *Pelobates fuscus* 1915.9.15.6; *Pelobates syriacus* 1971.1367; **Scaphiopodidae**: *Scaphiopus holbrooki* (*solitarius*) 233; *Spea (Scaphiopus) bombifrons* 1964.2063.

Hyloides: Bufonidae: *Rhinella* (*Bufo*) *marina* 62.1.9.13; *Rhinella* (*Bufo*) *marina* 63.2.21.10; *Phrynoidis aspera* (*Bufo ?asper*) 62.3.20.4 (F); *Amietophrynus* (*Bufo*) *superciliaris*

1906.5.28.14.9 (F); *Amietophrynus (Bufo*) *mauretanicus* 1920.1.20.676; **Calyptocephalellidae**: *Calyptocephalella gayi* 1920.1.20.3930a (F); *Calyptocephalella gayi* 1920.1.20.3930b (M); *Calyptocephalella gayi* 110a; *Calyptocephalella gayi* 58.8.23.6; **Ceratophryidae**: *Ceratophrys aurita (dorsata*) 1933.11.21.1; *Ceratophrys ornata* 83.4.14.15; **Hylidae**: *Litoria* (*Cyclorana*) *australis* 64.7.22.25; *Litoria platycephala* (*Cyclorana platycephalus*) 1908.5.28.65 (F); *Osteopilis crucialis (Trachycephalus lichenatus*) 12.12.1.1; **Limnodynastidae**: *Helioporus albopunctatus* 1907.3.13.4 (M); *Limnodynastes dorsalis* 96.1.30.8 (M); *Limnodynastes tasmaniensis* 1901.9.13.9 (F); *Neobatrachus (Heleioporus) pictus* RR1937.7.29.10 (M); *Platyplectrum (Limnodynastes*) *spenceri* 97.10.27.69 (M); **Odontophryniidae**: *Odontophrynus americanus* 85.6.26.41; *Proceratophrys (Ceratophrys*) *boiei* 1901.7.29.23; **Rhinophrynidae**: *Rhinophrynus dorsalis* 1983.925; **Telmatobiidae**: *Telmatobius* (*Batrachophrynus*) *brachydactylus* 74.2.3.4; *Telmatobius jelskii* 60.6.16.119.

Ranoides: Ceratobatrachidae: *Ceratobatrachus guentheri* 95.12.28.14; **Dicroglossidae**: *Hoplobatrachus tigrinus* unnumbered; **Mantellidae**: *Boophis ('Rhacophorus') madagascariensis* 92.1.21.24 (M); **Microhylidae**: *Glyphoglossus molossus* 1965.1060.61; *Glyphoglossus molossus* 1965.1059 (M); *Kaloula pulchra* 62.1.9.10; *Kaloula pulchra* BM 1983; *Rhombophryne testudo* 86.2.25.33 (F); *Scaphiophryne* (*Pseudohemisus*) *madagascariensis* 95.10.29.89; *Uperodon systoma* 46.11.22.59; **Ptychadenidae**: *Hildebrandtia ornata budgetti* 1968.950 (F); **Pyxicephalidae**: *Pyxicephalus adspersus* 63.2.21.7; *Pyxicephalus adspersus* 12259 (M).

University of Cambridge Museum of Zoology

Ceratophrys ornata R.1530; *Lepidobatrachus asper* R.1556; *Osteopilus crucialis* Unnumbered; *Osteopilus brunnaus* Unnumbered.

UCL Grant Museum of Zoology

Ceratophrys sp. W186

Los Angeles County Museum

Ceratophrys aurita (catalogued as *C. varia*) LACM 163430 (F).

Section F. References cited in Sections A–E.

1. Frost DR (2013) Amphibian Species of the World: an Online Reference. Version 5.6 (9 January 2013). Electronic Database accessible at http://research.amnh.org/herpetology/amphibia/index.html. American Museum of Natural History, New York, USA. Accessed August 2013

2. Fabrezi M (2006) Morphological evolution of the Ceratophryinae (Anura, Neobatrachia). J Zool Syst Evol Res 44: 153–166.

3. Evans SE, Jones MEH, Krause DW (2008) A giant frog with South American affinities from the Late Cretaceous of Madagascar. Proc Natl Acad Sci USA 105: 2951–2956.

4. Báez AM, Moura GJB, Gómez RO (2009) Anurans from the Lower Cretaceous Crato Formation of northeastern Brazil: implications for the early divergence of neobatrachians. Cretaceous Res 30: 829–846.

Figures for Supporting Information.

Figure S1. Sensitivity analysis 1: morphological dataset. No character 6, no *Thaumastosaurus*, no *Cratia*, no hyperossified characters.

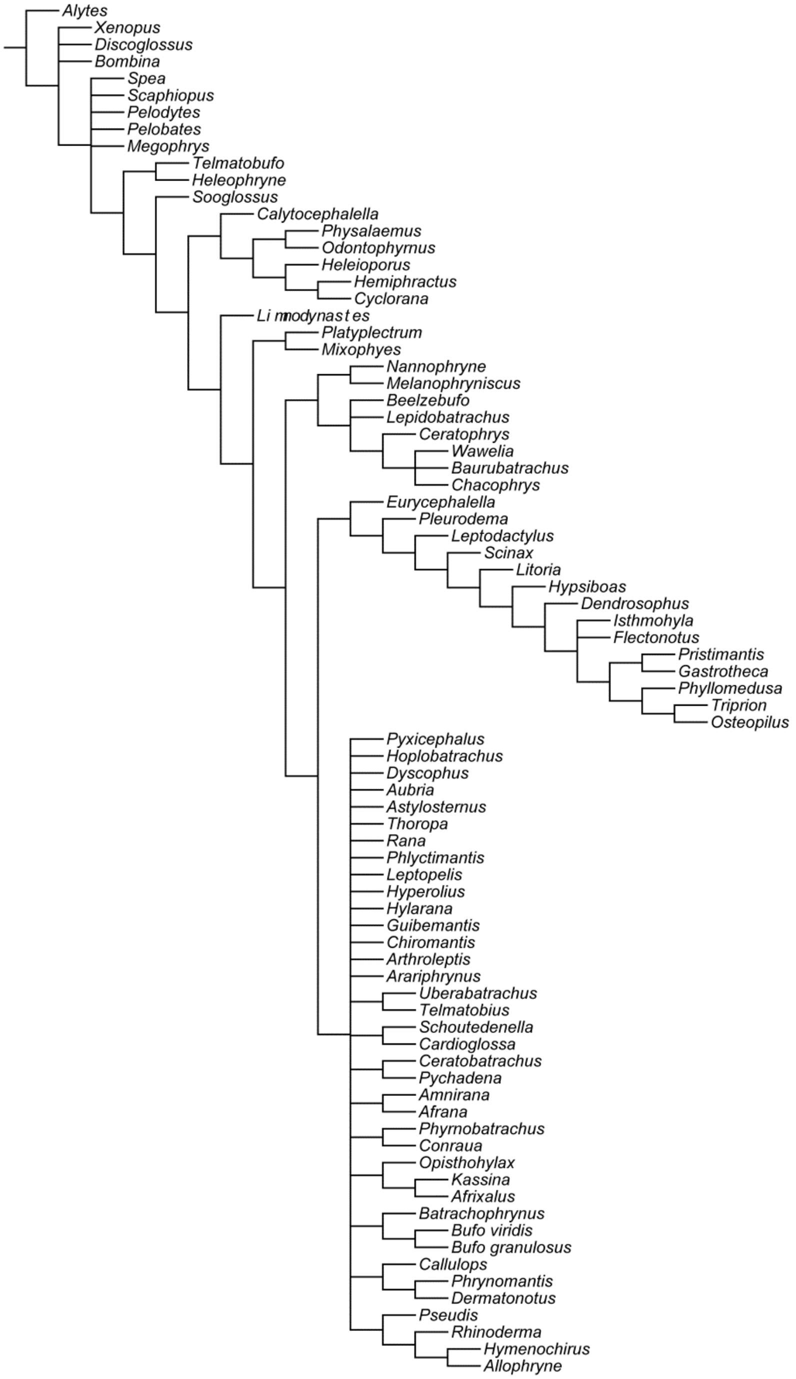

Figure S2. Sensitivity analysis 2: morphological dataset. No character 6, no *Thaumastosaurus*, no *Cratia*, only hyperossified characters.

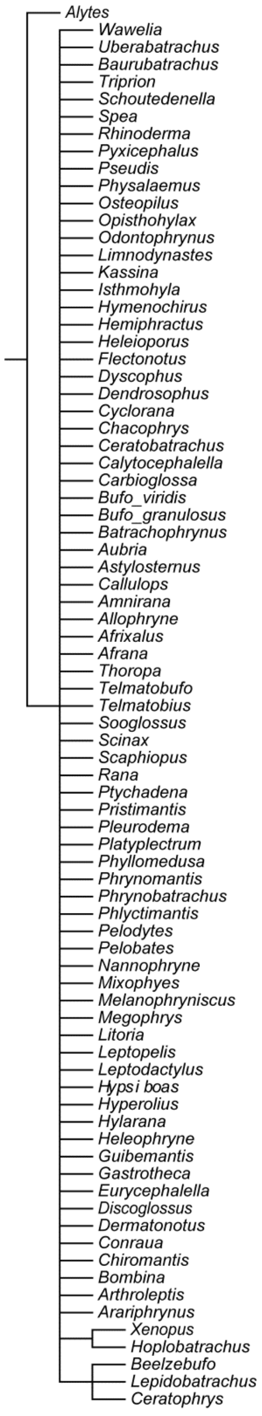

Figure S3. Sensitivity analysis 3: morphological dataset. No character 6, no *Thaumastosaurus*, no *Cratia*, no *Wawelia*.

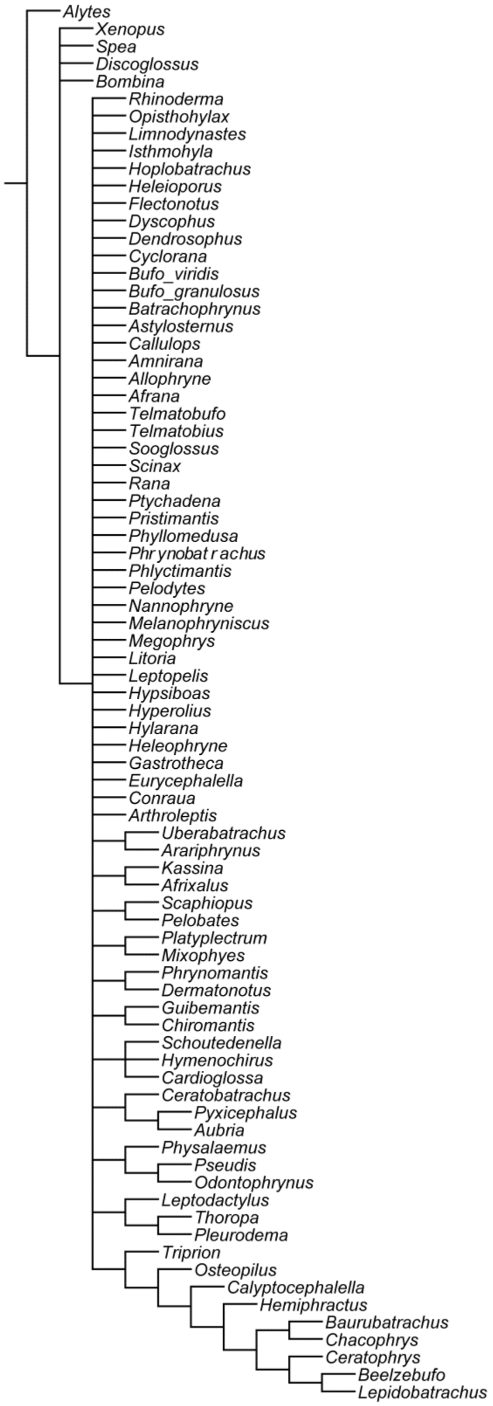

Figure S4. Sensitivity analysis 4: morphological dataset. No character 6, no *Thaumastosaurus*, no *Cratia*, no *Baurubatrachus*.

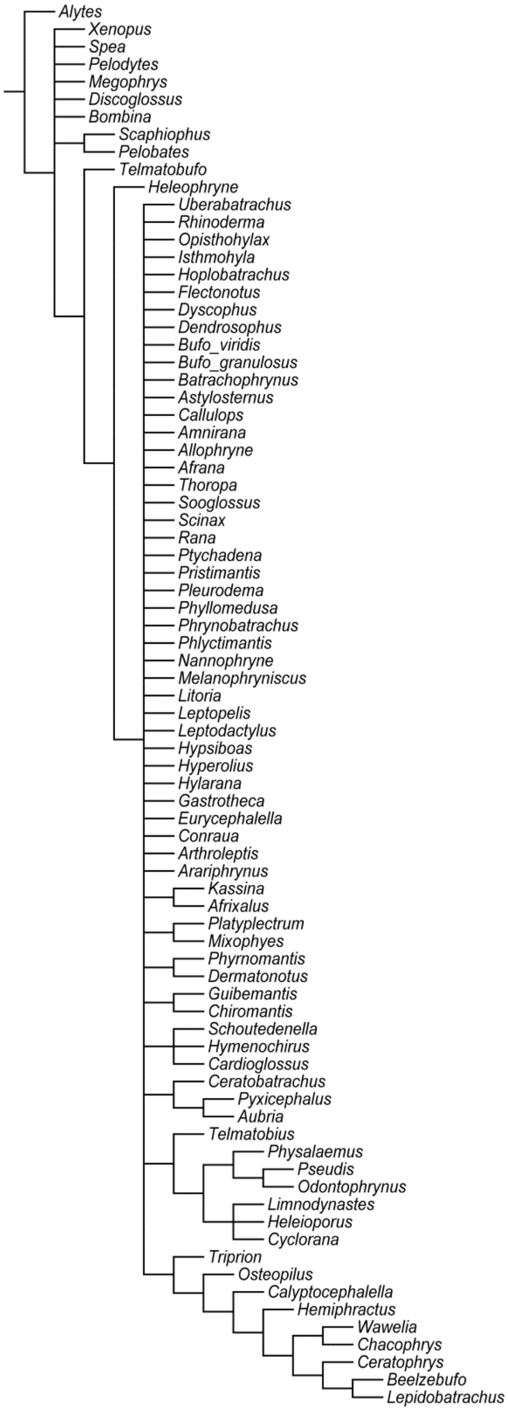

Figure S5. Sensitivity analysis 5: morphological dataset. No character 6, no *Thaumastosaurus*, no *Cratia*, no *Wawelia*, no *Baurubatrachus*.

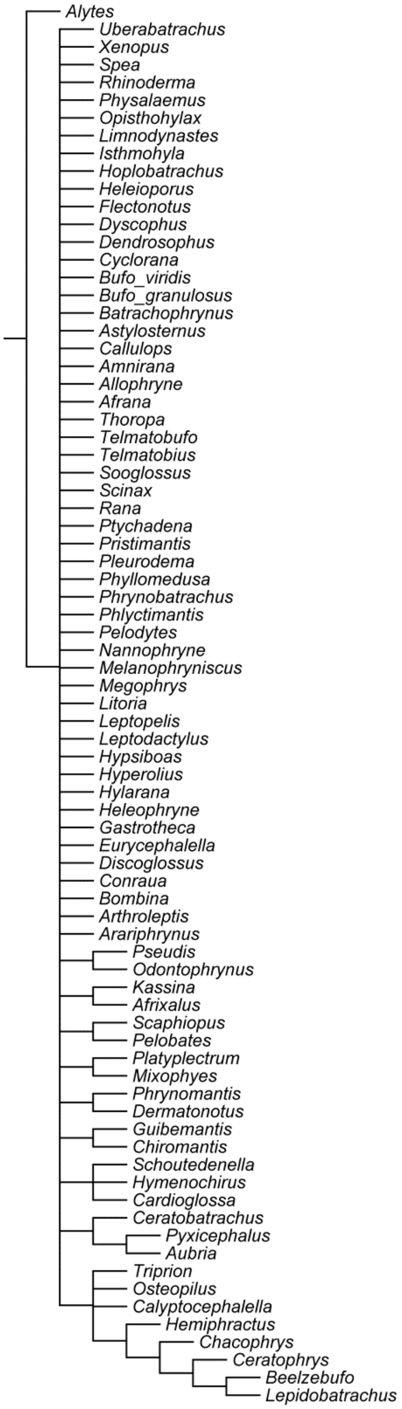

Figure S6. Sensitivity analysis 6: combined dataset. No character 6, no *Thaumastosaurus*, no *Cratia*, no hyperossified characters.

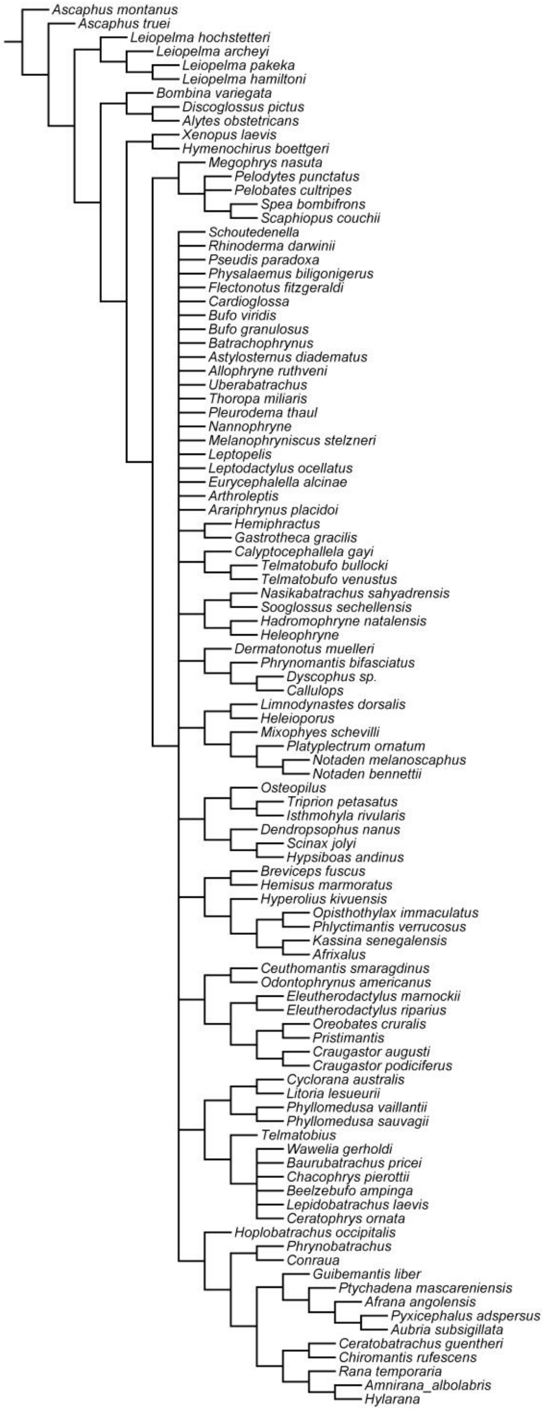

Figure S7. Sensitivity analysis 7: combined dataset. No character 6, no *Thaumastosaurus*, no *Cratia*, no *Baurubatrachus*.

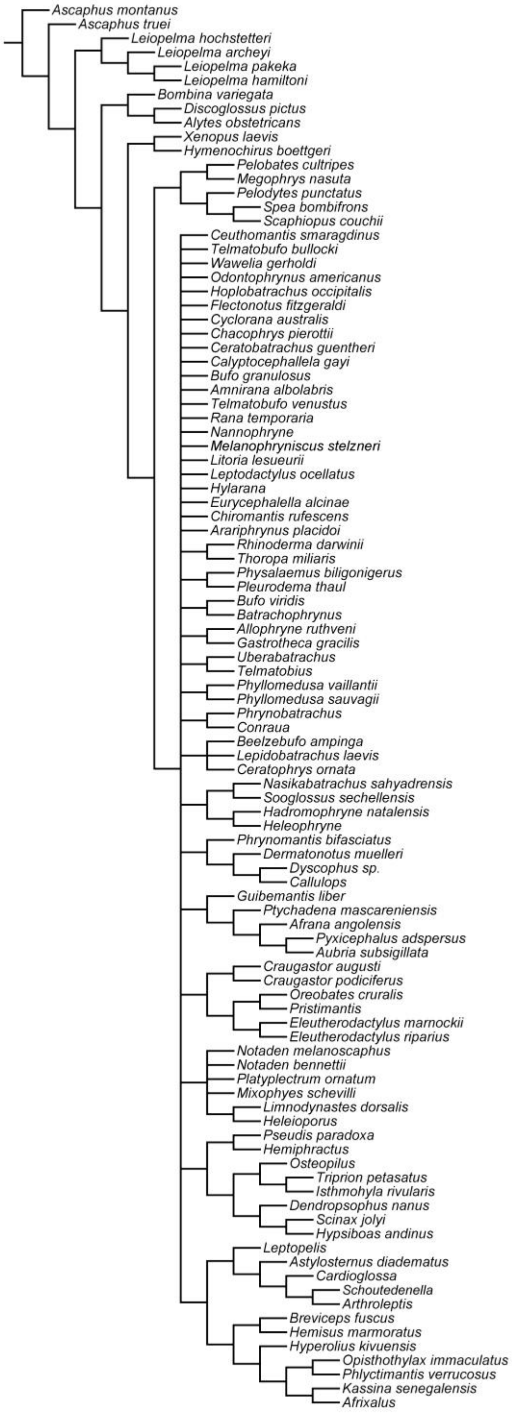

Figure S8. Sensitivity analysis 8: combined dataset. No character 6, no *Thaumastosaurus*, no *Cratia*, no *Wawelia*.

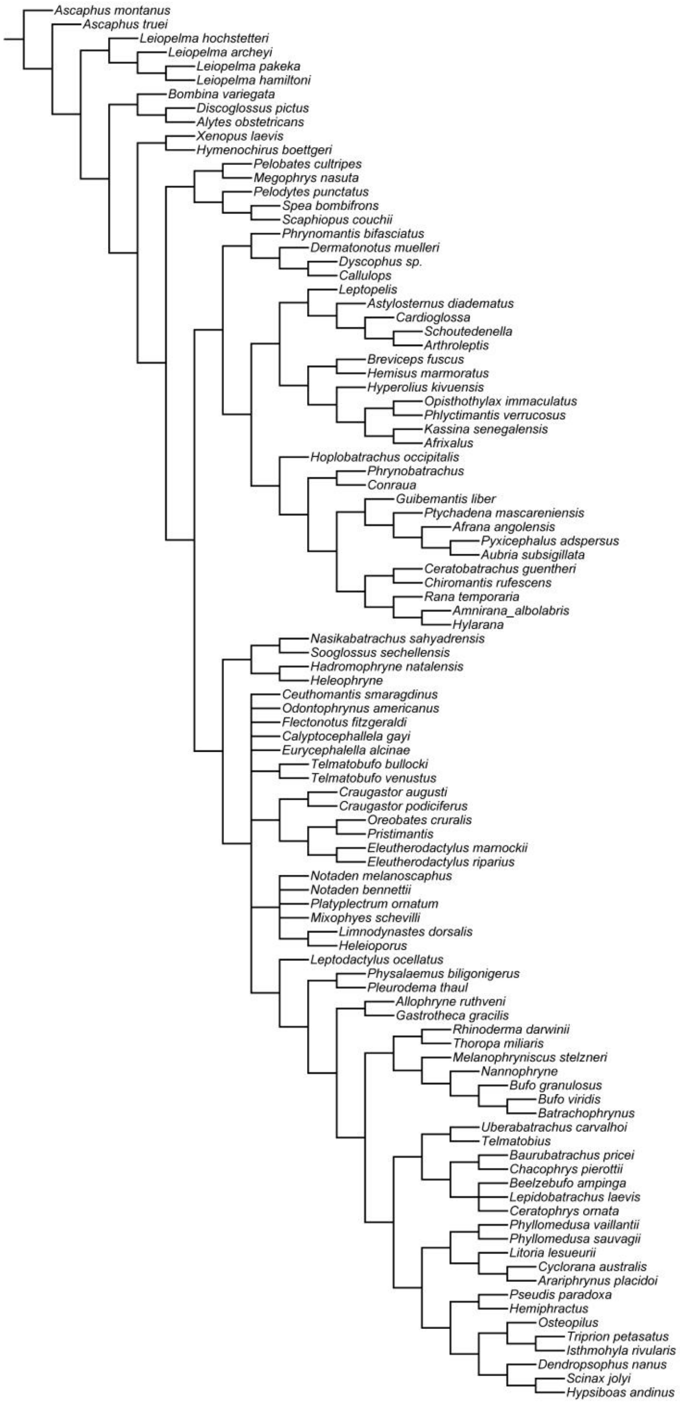

Figure S9. Sensitivity analysis 9: combined dataset. No character 6, no *Thaumastosaurus*, no *Cratia*, no *Wawelia*, no *Baurubatrachus*.

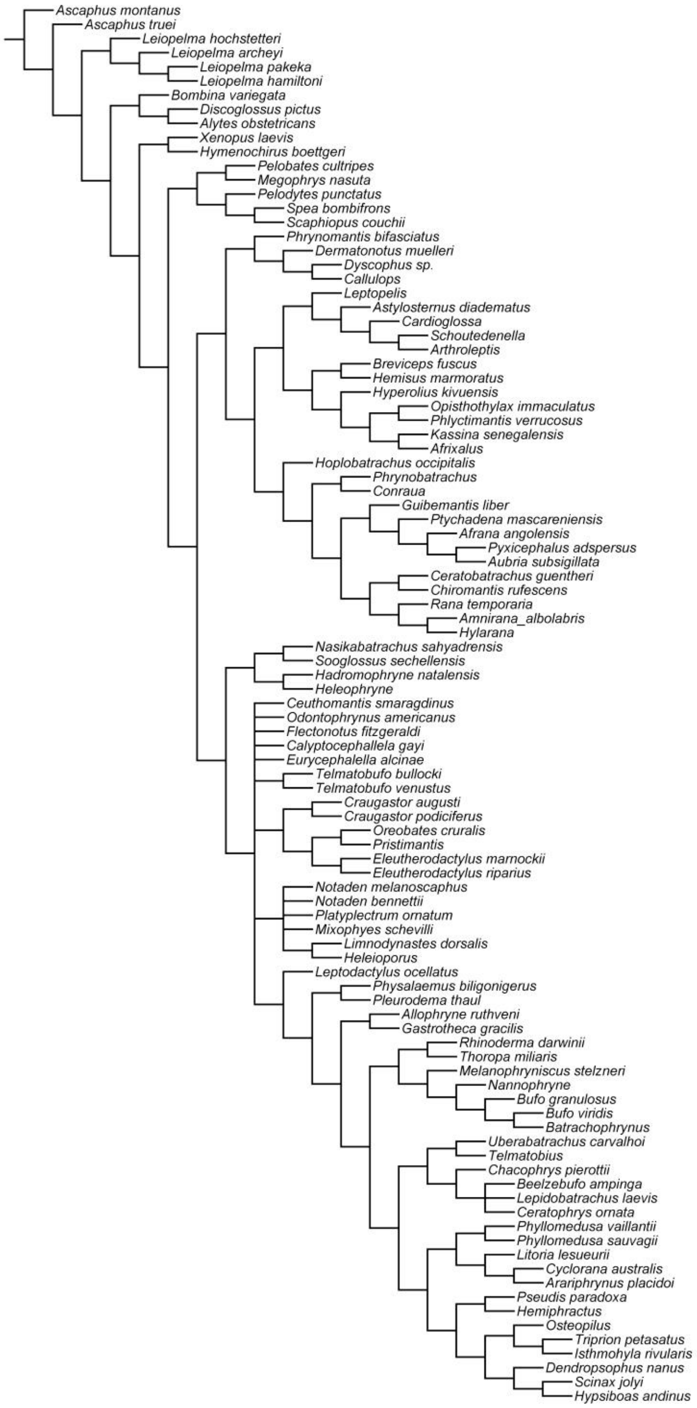

Tables.

Table S1. Changes in specimen numbers and identifications from Evans et al. (2008).

Original PNAS #(s)	Revised #	Original identification	Revised identification
FMNH PR 1959		maxillary process, right squamosal	partial right quadratojugal
FMNH PR 2497		?midline roofing element	cranial or vertebral spine table fragment
FMNH PR 2500		nasal (side indet.)	partial posterior process of right quadratojugal
FMNH PR 2501–2503	PR 2501	nasal fragments (side indet.)	partial right quadratojugal
FMNH PR 2508		fragment of ?frontoparietal	cranial fragment from antorbital margin (nasal or frontoparietal)
FMNH PR 2509		midline cranial element	conjoined frontoparietal fragment
FMNH PR 2512	FMNH PR 2512	left frontoparietal	left squamosal with facet for frontoparietal in parieto-squamosal bridge
FMNH PR 2513	FMNH PR 2512	right nasal	left quadratojugal, posterior-most portion of posterior process
FMNH PR 2514	FMNH PR 2512	nasal (side indet.)	cranial fragment
FMNH PR 2515	FMNH PR 2512	left squamosal	left anterior quadratojugal with part of squamosal suture
FMNH PR 2516	FMNH PR 2512	right ?frontoparietal	fragment of median suture region, ?frontoparietal
FMNH PR 2517	FMNH PR 2512	right frontoparietal	cranial fragment
FMNH PR 2518	FMNH PR 2512	left maxilla	facial process of left maxilla including portion of suture with nasal
FMNH PR 2519	FMNH PR 2512	left palatoquadrate + quadratojugal	left quadrate sutured to fragment of left quadratojugal
FMNH PR 2520	FMNH PR 2512	indet. cranial part	left quadratojugal, dorsal fragment of posterior process
FMNH PR 2522	FMNH PR 2512	part, right nasal	cranial or vertebral spine table fragment
FMNH PR 2523	FMNH PR 2512	part, dorsal margin of ?maxilla	vertebral spine table
FMNH PR 2524	FMNH PR 2512	indet. cranial fragment	cranial fragment
FMNH PR 2525	FMNH PR 2512	indet. cranial fragment	cranial fragment
FMNH PR 2526	FMNH PR 2512	part, fused cranial elements	cranial fragment
FMNH PR 2529	FMNH PR 2512	part, ?dorsal shield	fragment of bony plate (?osteoderm)
FMNH PR 2530	FMNH PR 2512	part, cranial element	cranial fragment
FMNH PR 2531	FMNH PR 2512	part, cranial element	cranial fragment
FMNH PR 2536		?squamosal, otic ramus	right fused squamosal-quadratojugal flange
FMNH PR 2537		indet. cranial fragment	squamosal fragment
UA 9614		maxillary process, right nasal	posteroventral process of right squamosal
UA 9615		left frontoparietal	cranial fragment from antorbital margin (nasal or frontoparietal)
UA 9617		part of ?squamsoal (side indet.)	posterior-most tip of left quadratojugal
UA 9618		?quadratojugal	fragment of left quadratojugal
UA 9619		?part of dorsal shield	vertebral spine table
UA 9623		left nasal	fragment of otic and ventral processes of squamosal
UA 9624		right nasal	posterior process of left quadratojugal
UA 9625		maxillary process, right nasal	posteroventral process of right squamosal
UA 9626		?nasal	cranial fragment
UA 9627		indet. cranial element	partial vertebral spine table
UA 9628		tibiofibula (side indet.)	right tibiofibula
UA 9629/9630	9629	left nasal	four fragments of left squamosal
UA 9631		left nasal	fragment of right squamosal suture with frontoparietal

UA 9632		?nasal fragment	cranial or vertebral spine table fragment
UA 9633		right frontoparietal	?partial frontoparietal or nasal
UA 9637		left nasal	cranial or vertebral spine table fragment
UA 9638		left nasal	otic plate of right squamosal
UA 9639		maxillary process, left squamosal	midportion of left quadratojugal
UA 9674		anterior ramus, right nasal	posterior process of left quadratojugal
UA 9676		part of maxilla	fragment of right maxilla bearing maxillary nerve canal
UA 9677		left frontoparietal	fragment of frontoparietal or squamosal
UA 9678		squamosal, ?possible otic process	partial vertebral spine table
UA 9679		possible juvenile quadratojugal	cranial fragment
UA 9680	FMNH PR 2512	juvenile squamosal, ?tip of otic process	vertebral spine table
UA 9681		?part of right ilium	*Madtsoia madagascariensis* proximal rib

Changes to museum catalogue numbers and identifications as published in: Evans SE, Jones MEH, Krause DW (2008). A giant frog with South American affinities from the Late Cretaceous of Madagascar. Proc Natl Acad Sci USA 105: 2951–2956. Institutional Abbreviations: FMNH, The Field Museum, Chicago, Illinois, U. S. A.; UA, Université d'Antananarivo, Antananarivo, Madagascar.

Table S2. *Beelzebufo* specimens used in the skeleton and skull reconstructions.

Specimen #	Identification	Transformations	Model position(s)
FMNH PR 2512 cranium	partial posterior cranium	mirrored portions of posterior cranium, pars facialis of left maxilla	posterior cranium and portions of pars facialis of right and left maxillae
FMNH PR 2512 Vertebra A	presacral vertebra	none	presacral vertebra 4
FMNH PR 2512 Vertebra B	partial spinous process of presacral vertebra	mirrored along midline	spinous process of presacral vertebra 5
FMNH PR 2512 urostyle	partial urostyle	none	proximal right cotyle of urostyle
FMNH PR 2507	pars facialis of right maxilla	mirrored	pars facialis of left and right maxillae
FMNH PR 2510	anterior portion of left maxilla	mirrored; cloned and mirrored portions of tooth row	left and right maxillae; portions of tooth row of left and right maxillae and premaxillae
UA 9615	partial right nasal or left frontoparietal	mirrored	posterior portions of left and right nasals
UA 9621	anterior portion of right quadratojugal	mirrored	anterior portion of left and right quadratojugals
UA 9951	anterior portion of left nasal	mirrored	anterior portion of left and right nasals
UA 9600	fused atlas and second presacral vertebrae	reduced isometrically (0.955296) to scale of FMNH PR 2512	centra of atlas and presecral vertebra 2
UA 9947	presacral vertebra	increased isometrically (1.06305)	presacral vertebra 3
FMNH PR 2300	sacral vertebra	mirrored along midline	sacral vertebra
UA 9636	urostyle	none	urostyle
UA 9628	right tibiofibula	none	right tibiofibula
UA 9957	right tibiale-fibulare	none	right tibiale-fibulare

Table S3. Axial column element lengths of representative anuran taxa.

Taxon	Catalogue #	Atlas (mm)	PS2 (mm)	PS3 (mm)	PS4 (mm)	PS5 (mm)	PS6 (mm)	PS7 (mm)	PS8 (mm)	Sacral (mm)	Total presacral series (mm)	Urostyle (mm)
Amietophrynus mauretanicus	BMNH 1920.1.20.676	3.4	2.5	2.8	2.8	2.8	3.2	3	3.2	1.9	25.6	22.1
Amietophrynus superciliaris	BMNH 1906.5.28.14.9	6.9	5	7.3	6.95	6.9	6.2	6.2	6.5	3.35	55.3	46.5
Boophis madagascariensis	BMNH 92.1.21.24	2.6	2.1	2.4	2.5	2.5	2.5	2.5	2.8	1.6	21.5	20.7
Calyptocephalella gayi	BMNH 110	7	5	5.8	6.1	6.1	5	4.5	5.3	4	48.8	49.2
Calyptocephalella gayi	BMNH 1920.1.20.3930F	9	6.2	6.3	7.4	7.4	6.6	6.7	6.7	4.8	61.1	52
Calyptocephalella gayi	BMNH 1920.1.20.3930M	5.9	4.8	4.8	4.8	5.2	4.6	4.4	5	3	42.5	39.4
Ceratobatrachus guentheri	BMNH 95.12.28.14	2.5	1.8	2.2	2.4	2.4	2	2.2	2	1.4	18.9	22.6
Ceratophrys aurita	LACM 163430	16	7.8	9.6	11	11	11	8.8	8.6	6.7	90.5	42.3
Glyphoglossus molossus	BMNH 1965.1060.61	2.8	2	2.3	2.9	2.9	2.9	2.9	3.7	1	23.4	
Glyphoglossus molossus	BMNH 1965.1059	2.9	2.2	2.4	2.6	2.9	2.9	3.4	3.4	1.5	24.2	18
Helioporus allopunctatus	BMNH 1907.3.13.9	4 [1]		3.2	2.6	2.8	2.8	2.8	2.8	1.8	22.8	19
Hildebrandtia ornata	BMNH 1968.950	4.2 [1]		2.3	2.3	2.4	2.4	2.4		2	18	18.9
Hoplobatrachus tigrinus	BMNH Uncat.	6.2	4.2	5	6	5.6	5.6	5.8	5.5	3.35	47.3	42.4
Kaloula pulchra pulchra	BMNH 62.1.9.10	2.6	2.3	2.3	2.9	3.3	3.2	3.4	3.2	2.3	25.5	
Kaloula pulchra pulchra	BMNH 1983	2.9	2.5	2.5	3	3.4	3.3	3.4	3.4	2.2	35.6	19.8
Limnodynastes dorsalis	BMNH 96.1.30.8	2.7 [1]		1.8	1.9	1.9	1.5	1.7	1.7	1.4	14.6	15.9
Limnodynastes tasmaniensis	BMNH 1901.9.13.9	2 [1]		1.4	1.2	1.4	1.5	1.5	1.5	1	11.5	10.5
Litoria australis	BMNH 64.7.22.25	3.7	3	3.4	3.4	4	3.6	2.4	2	2.7	28.2	25.4
Litoria playcephalus	BMNH 1908.5.28.65	2.8	2.5	2.6	2.8	2.8	2.8	2.8	2.5	1.6	23.2	
Neobatrachus pictus	BMNH 1937.7.29.10	3.3		2.2	1.8	1.9	1.9	1.9	1.9	1.2	16.1	12.4
Pelobates cultripes	BMNH 1920.1.20.682	3.6	2.5	2.9	3.2	3.2	3.4	3.5	3.4	2.3	28	15.7
Pelobates fuscus	BMNH 1920.1.20.680	2.8	2.1	2.3	2.4	2.9	2.5	2.5	2.8	1.7	22	11.9
Pelobates fuscus	BMNH 1915.9.15.6	2.9	1.9	2.3	2.4	2.55	2.6	2.7	2.3	2	21.6	12.3
Phrynoidis asper	BMNH 62.3.20.4	5.5	4.4	5	5.4	5.6	5.4	6	4.4	3.5	45.1	37.2
Platyplectrum spenceri	BMNH 97.10.27.69	2.6 [1]		1.7	1.4	1.4	1.4	1.4	1.5	0.8	12.2	10
Rhinella marina	BMNH 62.1.9.13	5.6	4.2	4.9	5.2	4.6	4.7	5.1	4.7	3.2	42.1	32
Rhombophryne testudo	BMNH 86.2.25.33	2.1	1.5	1.6	1.6	1.6	1.6	1.6	1.9	1.4	14.9	16.6
Scaphiophryne madagascariensis	BMNH 95.10.29.89	1.2	1.2	1.5	1.8	1.7	2	2	2	1.2	14.6	
Scaphiopus holbrooki	BMNH 233	3.5	1.5	2.3	3	3.6	3.3	3.8	3.5	2.8	27.3	13.6
Uperodon systema	BMNH 46.11.22.59	3.2	2.1	2.4	2.8	3	3.6	3.4	3.2	2	25.7	17

[1] Atlas and second presacral vertebrae are fused without obvious suture. Length given under atlas includes length of second presacral.
Empty cells indicate missing, obscured, or damaged elements that could not be measured

Table S4. Measurements and proportions of tibiofibula and tibiale-fibulare of representative anuran taxa.

Taxon	Catalogue #/source	Length of tibiofibula (mm)	Length of fibulare (mm)	Tibiofibula/ fibulare	Tibiale-fibulare proximal width (mm)	Tibiale-fibulare distal width (mm)	Tibiale-fibulare proximal/ distal width	Tibiale-fibulare length/ distal width	Length of centrum of presacral 4 (mm)	Length of fibulare/ length of PS4 centrum	Length of tibiofibula/ length of PS4 centrum
Beelzebufo (tibia as preserved)	UA 9628 & UA 9957[1]	51.3	24.6		11.1	14.2	0.78		7.7		
Beelzebufo (tibia ends estimated)	UA 9628 & UA 9957[1]	56–62	28–30	1.86–2.21	11.1	14.2	0.78	2.00–2.11	7.7	3.64–3.90	7.27–8.05
Amietophrynus mauretanicus	BMNH 1920.1.20.676	24.2	13.7	1.77	5.3	5.3	1.00	2.58	2.8	4.89	8.64
Amietophrynus superciliaris	BMNH 1906.5.28.14.9F	48.4	27	1.79	9.3	9.3	1.00	2.90	7	3.86	6.91
Boophis madagascariensis	BMNH 92.1.21.24	39	20.5	1.90	3.6	3.3	1.09	6.21	2.5	8.20	15.60
Calyptocephalella gayi	BMNH 111a	45	25.6	1.76	8.9	11.2	0.79	2.29	6.1	4.20	7.38
Calyptocephalella gayi	BMNH 1920.1.20.3930F	50.5	28.8	1.75	11.7	14	0.84	2.06	7.4	3.89	6.82
Calyptocephalella gayi	BMNH 1920.1.20.3930M	40	22.9	1.75	9.3	10.4	0.89	2.20	4.8	4.77	8.33
Ceratobatrachus guentheri	BMNH 95.12.28.14	28.9	14	2.06	4.3	3.9	1.10	3.59	3.4	4.12	8.50
Ceratophrys aurita	LACM 163430	54.6	33.2	1.64	14.8	14.4	1.03	2.31	6.4	5.19	8.53
Glyphoglossus molossus	BMNH 1965.1059M	21.5	8.2	2.62	7	6.5	1.08	1.26	2.6	3.15	8.27
Glyphoglossus molossus	BMNH 1965.1060.61	21.6	9.4	2.30	7.2	7.2	1.00	1.31	2.9	3.24	7.45
Heleioporus allopunctatus	BMNH 1907.3.13.4M	22.8	11.9	1.92	6	7.2	0.83	1.65	2.6	4.58	8.77
Hildebrandtia ornata	BMNH 1968.950F	25	12.4	2.02	4	4.5	0.89	2.76	2.4	5.17	10.42
Hoplobatrachus tigrinus	BMNH Uncat.	67.5	33.7	2.00	11	12	0.92	2.81	6	5.62	11.25
Kaloula pulchra pulchra	BMNH 62.1.9.10	18.8	10.4	1.81	4.4	5.4	0.81	1.93	2.9	3.59	6.48
Kaloula pulchra pulchra	BMNH 1983	18.6	10.1	1.84	4.4	4.6	0.96	2.20	3	3.37	6.20
Limnodynastes dorsalis	BMNH 96.1.30.8M	20.5	10.4	1.97	3.8	4.2	0.90	2.48	1.9	5.47	10.79
Limnodynastes tasmaniensis	BMNH 1901.9.13.9	13.6	7	1.94	2.7	3.1	0.87	2.26	1.2	5.83	11.33
Litoria australis	BMNH 64.7.22.25	34.2	17.6	1.94	6	5.4	1.11	3.26	3.4	5.18	10.06
Litoria platycephala	BMNH 1908.5.28.65F	22.7	11.45	1.98	4.7	5.1	0.92	2.25	2.8	4.09	8.11
Neobatrachus pictus	BMNH 1937.7.29.10M	17.2	9.7	1.77	4.8	5.3	0.91	1.83	1.8	5.39	9.56
Osteopilus crucialis	UCZM R.Uncat.	49	27.7	1.77	5.4	5	1.08	5.54	4.7	5.89	10.43
Pelobates cultripes	BMNH 1920.1.20.682	26.3	13.4	1.96	6.3	8	0.79	1.68	3.2	4.19	8.22
Pelobates cultripes	BMNH Uncat.	24.6	12.7	1.94	6	8.4	0.71	1.51			
Pelobates fuscus	BMNH 1920.1.20.680	18.8	9.2	2.04	4.6	6	0.77	1.53	2.4	3.83	7.83
Pelobates fuscus	BMNH 97.5.8.15	18.1	9.3	1.95	4.5	6.1	0.74	1.52	2.4	3.88	7.54
Pelobates fuscus	BMNH 1915.9.15.6	18.2	9.8	1.86	4.6	5.3	0.87	1.85	2.4	4.08	7.58
Phrynoidis asper	BMNH 62.3.20.4	43.5	23.4	1.86	9	9.2	0.98	2.54	5.4	4.33	8.06
Platyplectrum spenceri	BMNH 97.10.27.69M	14.3	7.1	2.01	2.4	3	0.80	2.37	1.4	5.07	10.21
Pyxicephalus adspersus	BMNH 63.2.21.7	50.4	22.1	2.28	12.2	13	0.94	1.70	6	3.68	8.40
Pyxicephalus adspersus	BMNH 12259M	21.4	10.5	2.04	4.9	5	0.98	2.10	2.6	4.04	8.23
Rhinella marina	BMNH 62.1.9.13	46.3	25	1.85	8.2	9.2	0.89	2.72	5.2	4.81	8.90

Rhinella marina	BMNH 63.2.12.10	46.5	26.6	1.75	9	9.6	0.94	2.77	5.9	4.51	7.88
Rhinophrynus dorsalis	BMNH 1983.925	13.8	5.5	2.51	6.3	6.7	0.94	0.82	1.5	3.67	9.20
Rhombophryne testudo	BMNH 86.2.25.33F	14.1	7.2	1.96	3.7	4	0.93	1.80	1.6	4.50	8.81
Scaphiophryne madagascariensis	BMNH 95.10.29.89	13.8	7.1	1.94	3	2.6	1.15	2.73	1.8	3.94	7.67
Spea bombifrons	BMNH 1964.2063	15	6.5	2.31	3.7	4.3	0.86	1.51			
Rhombophryne testudo	BMNH 86.2.25.33F	14.1	7.2	1.96	3.7	4	0.93	1.80	1.6	4.50	8.81
Uperodon systoma	BMNH 46.11.22.59	18.9	9.6	1.97	4.5	5	0.90	1.92	2.8	3.43	6.75
Buergeria oxycephalus	Digimorph[2]	*104*	*43*	2.42	*9.5*	*9.5*	1.00	4.53	*7*	6.14	14.86
Ceuthomantis smaraginus	Digimorph[2]	*50*	*32.8*	1.52	*5.7*	*5*	1.14	6.56	*3*	10.93	16.67
Chrixalus idioticus	Digimorph[2]	*74.3*	*39.3*	1.89	*8.6*	*8.6*	1.00	4.57	*6.4*	6.14	11.61
Chiromantis rufescens	Digimorph[2]	*92.3*	*47*	1.96	*10*	*8*	1.25	5.88	*7.4*	6.35	12.47
Gastrophryne usta	Digimorph[2]	*49.6*	*25.5*	1.95	*9.4*	*10*	0.94	2.55	*4.2*	6.07	11.81
Hamptophryne boliviana	Digimorph[2]	*48.2*	*24.2*	1.99	*7.2*	*8.2*	0.88	2.95	*3.6*	6.72	13.39
Incilius periglenes	Digimorph[2]	*39*	*25*	1.56	*7*	*8*	0.88	3.13	*4.3*	5.81	9.07
Ischnocema guentheri	Digimorph[2]	*62.9*	*30*	2.10	*6.4*	*5.8*	1.10	5.17	*3.3*	9.09	19.06
Nyctixalus pictus	Digimorph[2]	*103*	*53*	1.94	*9*	*9*	1.00	5.89	*7.7*	6.88	13.38
Philautus femoralis	Digimorph[2]	*92*	*56*	1.64	*9*	*9.2*	0.98	6.09	*7*	8.00	13.14
Polypedates leucomystax	Digimorph[2]	*99*	*41*	2.41	*11*	*8.7*	1.26	4.71	*7.7*	5.32	12.86
Rhacophorus reinardtii	Digimorph[2]	*94*	*40*	2.35	*9*	*9.4*	0.96	4.26	*7.3*	5.48	12.88
Theloderma stellatum	Digimorph[2]	*87*	*42*	2.07	*9*	*9.4*	0.96	4.47	*7.3*	5.75	11.92

[1] Elements are not from same individual and therefore proportions are only indicative; applies also to PS4 (FMNH PR 2512).
[2] Unscaled measurements taken from the Digimorph website, University of Texas (www.digimorph.org). Italicized measurements have no units and are for proportional comparisons only.

Empty cells indicate skeletal element either not preserved or damaged. Length of fibulare/distal width differentiates saltators (high values) from specialised burrowers (low values). Estimated proportion for *Beelzebufo* resembles living anurans that walk and sometimes burrow. Saltators also have relatively longer tibiofibulae and ankle bones.

Table S5. Skull measurements and proportions of representative anuran taxa.

Taxon	Catalogue number	Skull midline length (mm)	Skull bi-quadrate width (mm)	Trans-condylar width (mm)	Skull midline length/ bi-quadrate width	Bi-quadrate width/ transcondylar width
Beelzebufo ampinga	Reconstruction of FMNH PR 2512	83.4	106.3	17.1	0.78	6.22
Amietophrynus mauretanicus	BMNH1920.1.20.676	17	22.4	6	0.76	3.73
Amietophrynus mauretanicus	BMNH 62.1.9.13	29	35	9	0.83	3.89
Amietophrynus superciliaris	BMNH 1906.5.28.14.9	30.1	49	8.7	0.61	5.63
Boophis madagascariensis	BMNH 95.10.29.89	20.7	21.8	5	0.95	4.36
Calyptocephalella gayi	BMNH 110	39.5	54.6	11.4	0.72	4.79
Calyptocephalella gayi	BMNH 1920.1.20.3930F	47.7	69.5	13.3	0.69	5.23
Ceratobatrachus guentheri	BMNH 95.12.28.14	26.5	30.8	5.2	0.86	5.92
Ceratophrys aurita	BMNH 1933.11.21.1	67.8	107.5		0.63	
Ceratophrys aurita	LACM 163430	60.2	97.0	18.6	0.62	5.23
Ceratophrys ornata	BMNH 83.4.14.15		44	6.6		6.67
Ceratophrys ornata	UCZM R1530	36.5	49.6	8.6	0.74	5.77
Ceratophrys sp.	UCL W186	45.3	70.1	11	0.65	6.37
Glyphoglossus molossus	BMNH 1965.1060.61	13.4	12	5.8	1.12	2.07
Heleioporus allopunctatus	BMNH 1907.3.13.4	22.6	29.4	7.4	0.77	3.97
Hildebrandtia ornata	BMNH 1968.950	19.6	20.6	5	0.95	4.12
Hoplobatrachus tigerinus	BMNH Uncat.	42	45.6	10.5	0.92	4.34
Kaloula pulchra pulchra	BMNH 62.1.9.10	13.5	19.7	5.8	0.69	3.40
Kaloula pulchra pulchra	BMNH 1983	14.9	20.3	5.8	0.73	3.50
Lepidobatrachus asper	UCZM R1556	22.7	38	5.6	0.60	6.79
Limnodynastes dorsalis	BMNH 96.1.30.8	17	22.4	5.1	0.76	4.39
Limnodynastes tasmaniensis	BMNH 1901.9.13.9	12	12	3.5	1.00	3.43
Litoria australis	BMNH 64.7.22.25	29.5	41	6.5	0.72	6.31
Litoria platycephala	BMNH 1908.5.28.65	16.3	26	5.3	0.63	4.91
Neobatrachus pictus	BMNH 1937.7.29.10	15.3	18.1	4.6	0.85	3.93
Osteopilus crucialis	BMNH 12.12.11	30.5	40	8.3	0.76	4.82
Pelobates cultripes	BMNH 1920.1.20.682	24.2	29.6	7.55	0.82	3.92
Pelobates fuscus	BMNH 1920.1.20.680	16.7	23	6.8	0.73	3.38
Pelobates fuscus	BMNH 1915.9.15.6	16.3	21.2	5.8	0.77	3.66
Phrynoidis asper	BMNH 62.3.20.4	30.4	43.4	8.4	0.70	5.17
Platyplectrum spenceri	BMNH 97.10.27.69	10.7	9.8	3.6	1.09	2.72
Pyxicephalus adspersus	BMNH 63.2.21.7	46	64.4	12.8	0.71	5.03
Pyxicephalus adspersus	BMNH 12259	24.3	26.6	5.3	0.91	5.02
Rhinella marina	BMNH 63.2	32	48.6	8.6	0.66	5.65

Rhinophrynus dorsalis	BMNH 1983.925	13	13.8	4.3	0.94	3.21
Rhombophryne testudo	BMNH 86.2.25.33	10.1	14.6	4.4	0.69	3.32
Scaphiophryne madagascariensis	BMNH 95.10.29.89	11	15.2	4	0.72	3.80
Scaphiopus holbrooki	BMNH 233	21.4	27.7	7.2	0.77	3.85
Uperodon systoma	BMNH 46.11.22.59	14.1	18	5.9	0.78	3.05

Empty cells indicate relevant dimension could not be measured due to missing or damaged skeletal components in specimen.

Table S6. Molecular divergence dates for Hyloidea

Author(s)	Date	MTDNA or nuclearDNA	Neobatrachia (Ma)	Phthanobatrachia (Ma)	Notogaeanura (Ma)	Nobleobatrachia (Ma)	Inclusive Ceratophryidae sensu Frost et al. 2006 (Ma)	Ceratophryidae sensu Pyron and Wiens 2011 (Ma)	*Ceratophrys-Lepidobatrachus* split (Ma)	Notes
Vences et al.	2003	Both		~170		~130 [1]				
Zhang et al.	2005	MTDNA		173 (152–195)		97 (81–115)[2]				
San Mauro et al.	2005	nuclearDNA	161.7 (128–199)	149.7 (117–186)	138 (108–171.6)	65 (51.9–82.8)				
van der Meijden et al.	2005	Both		133.6 (99.2–176.7)						
Roelants et al.	2007	Both	166.7 (149.3–185.7)	161.3 (143.3–179.9)	145.6 (129–163.9)	63 (52.6–77.1)		53.2 (43.6–66.1)	18.8 (12.7–26.1)	Bayesian Relaxed Clock Model (Thorne and Kishino 2002)
Roelants et al.	2007	Both	150.3 (141.3–171.9)	142 (130.1–161.9)	130 (119.3-151.2)	58 (49.4–70.8)		48.7 (41.1–59.2)	18.4 (9.8–27.2)	Penalized Likelihood Model
Roelants et al.	2007	Both	160.9 (143.9-179.3)	155.5 (139.1–173.8)	142 (124.3–160.5)	63.2 (52.7–75.9)	48.8 (40.8–60.5)		15.2 (10–21.3)	Constrained to match Frost et al. (2006)
Wiens	2007	nuclearDNA	107.635			92.31				Based on Anura-Caudata split at 250 Ma
Wiens	2007	nuclearDNA	124.65			106.43				Based on Anura-Caudata split at 300 Ma
Wiens	2007	nuclearDNA	154.85			132.84				Based on Anura-Caudata split at 350 Ma
van der Meijden et al.	2007	nuclearDNA		151 (116–197)						
Igawa et al.	2008	MTDNA		170 (140-202)		97 (65–133)[2]				
Heinicke et al.	2009	Both				73.9 (53–99.7)				
Wiens	2011	nuclearDNA	~155	~150	~135	~75		65.5 [3]	~20	Taken from Fig. 1: Penalized Likelihood
Wiens	2011	nuclearDNA	~210	~200	~175			~95	~40	Taken from Fig. 2: Bayesian analysis using BEAST (Drummond and Rambaut 2007)
Roelants et al.	2011	Both	170.5 (156.8–184.9)	162.5 (149.3–175.8)	155.4 (141–169.6)	68.2 (59.6–78)		~65.4 (56.6–75)[4]	24.1 (18.1–31.1)	Bayesian analysis using MDT (Thorne and Kishino 2002)

Roelants et al.	2011	Both	166.4 (146.3–187.4)	157.5 (138.5–177.8)	145.6 (126.1–166.5)	90.0 (76.3-106.6)		88.2 (73.7–103.7)[4]	19.3 (8.7–32.2)	Bayesian analysis using BEAST (Drummond and Rambaut 2007)
Ruane et al.	2011	Both				80.1 (74.5–85.2)			64 (61.7–66.3)	With *Beelzebufo* as crown-ceratophryid
Ruane et al.	2011	Both				71.4 (67.2–75.5)			13.6 (8.9–19.2)	With *Beelzebufo* as stem-ceratophryid
Ruane et al.	2011	Both				58.1 (52.2–64.5)			12 (7.6–16.7)	Without *Beelzebufo*
Irisarri et al.	2012	Both	~160 (187–135)	~150 (175–125)	~130	~80 (110–41)	~55 *Telmatobius*[5]			Taken from Fig. 2
Zhang et al.	2013	MTDNA	132 (114–153)	126.8 (109–147)	115.6 (99–135)	96.1 (82–113)		64.2 (53–77)		Bayesian analysis using MDT (Thorne and Kishino 2002)
Zhang et al.	2013	MTDNA	194.2 (162–229)	184.6 (154–219)	155.1 (124–194)	124.9 (99–157)		62.1 (43–81)		Bayesian analysis using BEAST (Drummond and Rambaut 2007)

[1]This is a minimum age as the authors used *Bufo* and *Leptodactylus* and this represents a more deeply nested node, Chthonobatrachia (Frost et al., 2006).

[2]This is a minimum age as the authors used only *Bufo* and *Hyla* and thus this represents a more deeply nested node, Athesphatanura (Frost et al. , 2006).

[3]Node calibrated on *Beelzebufo*

[4]Date estimate taken from node 66 roughly level with base of ceratophryid stem

[5]The authors used *Telmatobius* alone to represent Ceratophryidae sensu Frost et al. (2006).

References

Drummond AJ, Rambaut A (2007) BEAST: Bayesian evolutionary analysis by sampling trees. BMC Evol Biol 7: 214.

Frost DR, Grant T, Faivovich J, Bain RH, Haas A, et al. (2006) The amphibian tree of life. Bull Am Mus Nat Hist 297: 1–370.

Heinicke MP, Duellman WE, Trueb L, Means DB, MacCulloch RD, et al. (2009) New frog family (Anura: Terrana) from South America and an expanded direct-developed clade revealed by molecular phylogeny. Zootaxa 2211: 1–35.

Igawa T, Kurabayashi A, Usuki C, Fujii T, Sumida M (2008) Complete mitochondrial genomes of three neobatrachian anurans: a case study of divergence time estimation using different data and calibration settings. Gene 407: 116–129.

Irisarri I, San Mauro D, Abascal F, Ohler A, Vences M, et al. (2012) The origin of modern frogs (Neobatrachia) was accompanied by acceleration in mitochondrial and nuclear substitution rates. BMC Genomics 13: 626.

Pyron RA, Wiens, JJ (2011). A large-scale phylogeny of Amphibia including over 2800 species, and a revised classification of extant frogs, salamanders, and caecilians. Mol Phylogenet Evol 61: 543–583.

Roelants K, Gower DJ, Wilkinson M, Loader SP, Biju SD, et al. (2007) Global patterns of diversification in the history of modern amphibians. Proc Natl Acad Sci USA 104: 887–892.

Roelants K, Haas A, Bossuyt F (2011) Anuran radiations and the evolution of tadpole morphospace. Proc Natl Acad Sci USA 108: 8731-8736.

Ruane S, Pyron RA, Burbrink FT (2011) Phylogenetic relationships of the Cretaceous frog *Beelzebufo* from Madagascar and the placement of fossil constraints based on temporal and phylogenetic evidence. J Evol Biol 24: 274–285.

San Mauro D, Vences M, Alcobendas M, Zardoya R, Meyer A (2005) Initial diversification of living amphibians predated the breakup of Pangaea. Am Nat 165: 590–599.

Thorne JL, Kishino H (2002) Divergence time and evolutionary rate estimation with multilocus data. Syst Biol 51: 689–702.

van der Meijden A, Vences M, Hoegg S, Meyer A (2005) A previously unrecognised radiation of ranid frogs in Southern Africa revealed by nuclear and mitochondrial DNA sequences. Mol Phylogenet Evol 37: 674-685.

van der Meijden A, Vences M, Hoegg S, Boistel R, Channing A, et al. (2007) Nuclear gene phylogeny of narrow-mouthed toads (Family: Microhylidae) and a discussion of competing hypotheses concerning their biogeographical origins. Mol Phylogenet Evol 44: 1017–1030.

Vences M, Vieites DR, Glaw F, Brinkmann H, Kosuch J, et al. (2003) Multiple overseas dispersal in amphibians. Proc Roy Soc Lond B Bio 270: 2435–2442.

Wiens JJ (2007) Global patterns of diversification and species richness in amphibians. Am Nat 170: S86–S106.

Wiens JJ (2011) Re-evolution of lost mandibular teeth in frogs after more than 200 million years, and re-evaluating Dollo's Law. Evolution 65: 1283–1296.

Zhang P, Zhou H, Chen YQ, Liu YF, Qu LH (2005) Mitogenomic perspectives on the origin and phylogeny of living amphibians. Syst Biol 54: 391−400.

Zhang P, Liang D, Mao R-L, Hillis DM, Wake DB, et al. (2013) Efficient sequencing of anuran mtDNSs and a mitogenomic exploration of the phylogeny and evolution of frogs. Mol Biol Evol online. doi: 10.1093/molbev/mst091.
